# Simultaneous TALEN-mediated knockout of chrysanthemum *DMC1* genes confers male and female sterility

**DOI:** 10.1038/s41598-020-72356-1

**Published:** 2020-09-30

**Authors:** Harue Shinoyama, Hiroaki Ichikawa, Ayako Nishizawa-Yokoi, Mikhail Skaptsov, Seiichi Toki

**Affiliations:** 1 Fukui Agricultural Experiment Station, Fukui, 918-8215 Japan; 2 Institute of Agrobiological Sciences, National Agriculture and Food Research Organization (NARO), Tsukuba, 305-8604 Japan; 3 Precursory Research for Embryonic Science and Technology (PRESTO), Japan Science and Technology Agency (JST), Saitama, 332-0012 Japan; 4 South Siberian Botanical Garden, Altai State University, Barnaul, Russia 656049; 5 Graduate School of Nanobioscience, Yokohama City University, Yokohama, 236-0027 Japan; 6 Kihara Institute for Biological Research, Yokohama City University, Yokohama, 244-0813 Japan; 7 Present Address: Department of Bioscience, Fukui Prefectural University, Awara, 910-4103 Japan

**Keywords:** Biological techniques, Biotechnology, Plant sciences

## Abstract

Genome editing has become one of the key technologies for plant breeding. However, in polyploid species such as chrysanthemum, knockout of all loci of multiple genes is needed to eliminate functional redundancies. We identified six cDNAs for the *CmDMC1* genes involved in meiotic homologous recombination in chrysanthemum. Since all six cDNAs harbored a homologous core region, simultaneous knockout via TALEN-mediated genome editing should be possible. We isolated the *CmDMC1* loci corresponding to the six cDNAs and constructed a TALEN-expression vector bearing a *CmDMC1* target site containing the homologous core region. After transforming two chrysanthemum cultivars with the TALEN-expression vector, seven lines exhibited disruption of all six *CmDMC1* loci at the target site as well as stable male and female sterility at 10–30 °C. This strategy to produce completely sterile plants could be widely applicable to prevent the risk of transgene flow from transgenic plants to their wild relatives.

## Introduction

Chrysanthemum (*Chrysanthemum morifolium* Ramat.) is one of the best-known cultivated ornamental flowers worldwide. Contemporary chrysanthemum cultivars are hexaploids with loss or gain of several chromosomes^1^ and display self-incompatibility^2^. Many agronomical traits have been introduced via conventional cross- and mutation breeding. However, these technologies are potentially limited by the gene resources available and the pathways modifiable by crossing and/or mutation.

Genetic transformation technologies are useful for introducing agronomical traits that cannot be achieved by conventional mutation breeding. Almost 50 ornamental flowers have been transformed to express modified traits^3^. In many countries, growing genetically modified (GM) plants is tightly regulated under the guidelines and directives of the Cartagena Protocol on Biosafety^4,5^. In particular, ornamentals require assessment for outcrossing to wild relatives prior to practical use.

Transgenic carnations (*Dianthus caryophyllus* L.) and roses (*Rosa* x *hybrida*) producing blue flowers are now marketed under the terms of the Cartagena Protocol. Carnation flowers generate little mature pollen, and no hybrids between carnations and their wild relatives have been reported so far^6^. Transgenic bluish roses are genetic chimeras whose transgenes are not transmitted to pollen^7^. In chrysanthemum, many useful traits have been introduced by transformation, including disease resistance^8,9^, resistance against insects and fungal disease^10^ and modified flower color^11^. However, transgenic chrysanthemums are not yet sanctioned for open field cultivation because of their cross-compatibility with wild relatives. F_1_ plants between non-GM commercial chrysanthemums and their wild relatives are known to be distributed widely in several habitats of the wild species^12^.

To inhibit transgene flow, Aida et al.^13^ reported periclinal L1 chimeric plants in chrysanthemum, as transgenes in the L1 layer are rarely transmitted to progeny. While this is very useful for modification of plant surface characteristics such as flower color, in order to use plants genetically modified to confer insect- or disease-resistance, induction of both male and female sterility by suppressing or disrupting genes involved in gametogenesis is required.

The protein DMC1 plays a critical role in meiotic homologous recombination. Knocking out or mutating of *DMC1* causes serious defects in meiotic DNA recombination, asynapsis and sterility in yeast^14^, mouse^15^, *Arabidopsis*^16^ and rice^17^. Shinoyama et al.^18^ introduced an RNA interference (RNAi) construct of a *DMC1* gene (designated *CmDMC1a* in this study) in chrysanthemum. Regenerated *CmDMC1a*-RNAi plants exhibited severe male sterility and a significant reduction in female fertility. However, due to their hexaploid nature and self-incompatible trait^2^, transgenic chrysanthemum plants showing both male and female sterility are needed to completely inhibit transgene flow. This study thus aimed to produce chrysanthemum plants with simultaneous knockout of all six endogenous *CmDMC1* loci using transcription activator-like effector nucleases (TALEN)-mediated genome editing technology.

To simultaneously disrupt all *DMC1* genes, we exploited the extraordinary level of conservation among DMC1 protein and *DMC1* cDNA sequences^19^, identifying suitable target sequences common to all six chrysanthemum *DMC1* genes. The resulting plants exhibited both male and female sterility, suggesting that knockout of all *DMC1* alleles could be used to induce stable male and female sterility in chrysanthemum and other highly polyploid plants, and thus prevent transgene flow from GM plants to their wild relatives.

## Results

### Isolation of cDNAs and partial sequences of *DMC1* genes from chrysanthemum

**Figure 1:**
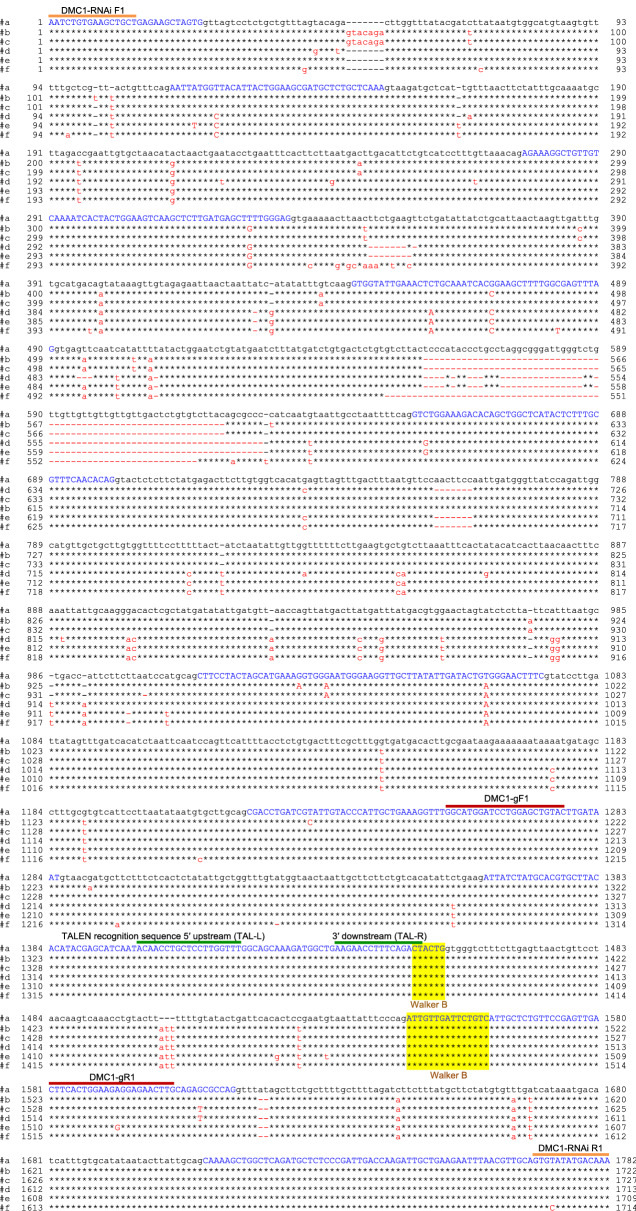
Partial genomic DNA sequences of the six *CmDMC1* genes of *Chrysanthemum morifolium* cultivar ‘Shuho-no-chikara’. Genomic DNAs corresponding to nucleotide positions 234–815 in the six cDNAs (Supplementary Fig. S1) are aligned with the DNASIS version 3.7 (Hitachi Software Engineering). Uppercase letters in blue indicate exon, and lower-case letters indicate intron. Asterisks (*) indicate the same nucleotides as in *CmDMC1a*; red letters indicate different nucleotides compared to *CmDMC1a*; red bar (-) indicates nucleotide deletion. TAL-L and TAL-R indicate the TALEN pair targeting *CmDMC1* genes. A primer pair, DMC1-RNAi F1 and DMC1-RNAi R1, was used to amplify partial DNA fragments of individual *CmDMC1* genes that correspond to cDNA nucleotide positions 234–815 of ‘Shuho-no-chikara’ (Supplementary Fig. S1). A primer pair, DMC1-gF1 and DMC1-gR1, was designed for the detection of mutations around the recognition sequences of the TALENs.

Based on the cDNA sequence of *CmDMC1a* (submitted to DDBJ under the accession number: LC575211)^18^, five additional *CmDMC1* cDNAs (*CmDMC1b*–*CmDMC1f*, the accession numbers: LC575212, LC575213, LC575214, LC575215 and LC575216, respectively) were isolated from chrysanthemum cultivar ‘Shuho-no-chikara’ (Supplementary Fig. S1). Nucleotide sequence similarities (homology) of each cDNA for *CmDMC1b* to *CmDMC1f* compared to *CmDMC1a* were 99.2%, 99.5%, 99.1%, 99.0% and 99.3%, respectively. Amino acid sequence identities between CmDMC1b to CmDMC1f and CmDMC1a were 99.1%, 99.1%, 98.3%, 98.0% and 99.1%, respectively (Supplementary Fig. S2). These six cDNA clones were also isolated from the other nine cultivars (Supplementary Table S1). No novel cDNA clones were found in any of the nine cultivars, suggesting that there are six active *DMC1* loci in chrysanthemum. Respective genomic DNAs corresponding to nucleotide positions 234–815 in the six cDNAs were isolated from ‘Shuho-no-chikara’. These sequences include the homologous core region used for the *CmDMC1a*-RNAi study^18^. Numbers of nucleotides varied among the six loci: 1782 bp (*CmDMC1a*), 1722 bp (*CmDMC1b*), 1727 bp (Cm*DMC1c*), 1713 bp (*CmDMC1d*), 1709 bp (*CmDMC1e*), and 1714 bp (*CmDMC1f*) (Fig. 1), possibly due to differences in the number of nucleotides in intron(s) between exons among *CmDMC1* genes. Since an exon sequence at nucleotide positions 1363 to 1452 bp in *CmDMC1a* (Fig. 1) is located in the conversed core region and identical to corresponding sequences in the other five *CmDMC1* genes, the target sequence for the TAL effector repeat array was designed in this region for simultaneous disruption of all *CmDMC1* alleles using the system TAL Effector Nucleotide Targeter 2.0 system developed by Cornell University (https://tale-nt.cac.cornell.edu/node/add/talen-old) (Fig. 1, Supplementary Table S2). This target sequence includes the multimer site (BRC) interface located upstream of the Walker B motif^20^.

### Production of *CmDMC1* knockout plants using TALEN-mediated targeted mutagenesis

**Figure 2:**
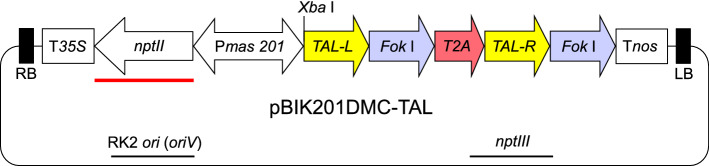
Structure of binary vector pBIK201DMC-TAL for disruption of six *CmDMC1* genes in chrysanthemum. RB, right border; LB, left border; P*mas201*, bidirectional promoter cassette from *mannopine synthase 1′* and *2′* (*mas1′-2′*) genes; T*35S*, Cauliflower mosaic virus *35S* terminator; T*nos*, *nopaline synthase* terminator; *nptII*, *neomycin phosphotransferase II* gene for the selection of transgenic plants; TAL-L and TAL-R, the TALEN pair targeting *CmDMC1* genes; *Fok* I, gene encoding a restriction enzyme. *T2A*, encoding a peptide for self-cleavage and ribosome skipping^69^. Red bar indicates the *nptII-*specific probe (about 800 bp) for Southern blotting in Supplementary Fig. S3.

Infection of chrysanthemum leaf discs with *Agrobacterium tumefaciens* strain EHA105^21^ harboring a binary vector pBIK201DMC-TAL containing the TALEN pair targeting *CmDMC1* loci (Fig. 2), and selection and regeneration of transgenic plants were performed as shown in Supplementary Table S3. From the two cultivars ‘Shuho-no-chikara’ and ‘Yamate-shiro’, 23 and 126 plantlets, respectively, were regenerated from calli resistant to 20 mg l^−1^ G418, giving regeneration rates of 3.2% and 17.5% from 719 and 720 initial leaf segments (Supplementary Table S4).

The presence and number of T-DNA copies in individual regenerated plants (defined as the T1 generation) were confirmed by Southern blot analysis of *Xba*I-digested genomic DNA probed with a fragment of the *neomycin phosphotransferase II* (*nptII*)*.* Theoretically, a unique fragment over 1.5 kbp (Fig. 2) could be detected when a single copy of T-DNA is integrated into the chrysanthemum genome. Single to multiple bands hybridizing to the *nptII* probe were observed in the regenerated plantlets. Among them, the results for four lines (SH#12, SHa#13, YS#16 and YSa#12) are shown in Supplementary Fig. S3. No hybridization signals were detected in non-transgenic chrysanthemum controls.

For the detection of mutations in individual *CmDMC1* genes (*CmDMC1a*–*f*), a fragment of about 300 bp containing the TALEN recognition sequences was amplified by PCR using the primer pair DMC1-gF1 and DMC1-gR1 (see Fig. 1), and genomic DNA from a *CmDMC1*-TALEN line as a template. Each DNA amplicon was cloned and sequenced to identify individual *CmDMC1* genes. Sequences of the clones carrying particular DNA amplicons were analyzed to identify and classify mutations in individual *CmDMC1* genes. Typically, three mutation patterns were detected in each TALEN-targeted locus. The first was a mutation in a single allele of a specific *CmDMC1* gene (monoallelic mutation). The rest were mutations in both alleles of a *CmDMC1* gene (biallelic mutations) that consisted of two types: an identical mutation in both alleles, and different mutations in the two alleles of a single *CmDMC1* gene.

DNA sequence analysis of six *CmDMC1* genes in the 23 *CmDMC1*-TALEN lines of ‘Shuho-no-chikara’ revealed that five lines showed biallelic mutations in all six loci (genotype e.g. *aabbccddeeff*, where each lowercase letter indicates a mutated allele for each *CmDMC1* locus). The remaining lines showed biallelic mutations in five loci, and a monoallelic mutation in one locus (genotype e.g. *aaBbccddeeff*, where the uppercase *B* indicates the wild-type allele and *Bb* denotes a monoallelic mutation in *CmDMC1b*) (Supplementary Table S4). Similarly, among 126 *CmDMC1*-TALEN lines of ‘Yamate-shiro’, two lines showed mutations in all six target loci (biallelic mutations) (Supplementary Table S4). For each cultivar, we selected seven lines that possessed biallelic mutations in all six *CmDMC1* genes, or in five genes with one wild-type sequence to elucidate the effect of each gene. Among a total of 84 *CmDMC1* genes (168 alleles) from 14 TALEN lines, 72 genes (144 alleles) were mutated. Deletion mutations were detected most frequently, and insertion mutations including a combination of insertion and deletion were detected only rarely (Table 1, Supplementary Table S5 and Supplementary Fig. S4). Biallelic mutation patterns with identical mutations in both alleles of a particular *CmDMC1* gene (e.g. *CmDMC1a, b, e* and* f* in SH#12) were detected in 37 genes (74 alleles). Biallelic and different mutation patterns in the two alleles of a *CmDMC1* gene (e.g. *CmDMC1c* and* d* in SH#12) were found in 35 genes (70 alleles) —a frequency similar to that of the identical mutation patterns (Supplementary Table S5). Frameshift mutations were caused by INDELs (insertion or deletion) of a number of nucleotides that is not divisible by three; in this case, stop codons appeared just downstream of the mutated site (Supplementary Table S6). On the other hand, INDELs involving multiples of 3 nucleotides caused insertion/deletion of a few amino acids (e.g. *CmDMC1d* in SHa#13 and SHf#20, and *CmDMC1e* in SHb#14 and YSc#27). Mutation patterns at each locus were examined in two different tissues, leaf and root, and found to be identical in these two tissues in every transgenic line analyzed (Table 1). These results suggested that the tissues (leaves and roots) in each regenerated transgenic plant were differentiated from a small callus possibly derived from a single or a few transgenic cells, and that each respective *CmDMC1* locus is represented by a specific mutation pattern following TALEN-mediated genome editing. To investigate whether additional mutation(s) occurred outside the *CmDMC1*-TALEN recognition sites, approximately 1.8 kbp fragments containing the 300 bp regions in the *CmDMC1* genes of the 14 TALEN lines were PCR-amplified using total DNAs from leaves or roots of respective lines as templates and the primer pair DMC1-RNAi F1 and DMC1-RNAi R1 (Supplementary Fig. S4), cloned and sequenced. Comparing the 1.8 kbp sequences for *CmDMC1a* in each TALEN-line with that of wild-type revealed that no additional mutations were generated outside the TALEN target regions (Supplementary Table S5 and Supplementary Fig. S4).

**Table 1:** Mutation patterns of *CmDMC1* alleles in leaves and roots.

Line^a^	No. of mutated *CmDMC1* alleles	Organ	Mutations detected in each *CmDMC1* gene^b,c^											
			*CmDMC1a*		*CmDMC1b*		*CmDMC1c*		*CmDMC1d*		*CmDMC1e*		*CmDMC1f*	
SH#12	12	Leaf	m	25-d1	m	19-d1	m	25-d2	m	26-d1	m	22-d1	m	20-d1
			m	25-d1	m	19-d1	m	40-i1	m	40-i1	m	22-d1	m	20-d1
		Root	m	25-d1	m	19-d1	m	25-d2	m	26-d1	m	22-d1	m	20-d1
			m	25-d1	m	19-d1	m	40-i1	m	40-i1	m	22-d1	m	20-d1
SH#a13	10	Leaf	n	–	m	27-d2	m	25-d2	m	26-d3	m	23-d2	m	20-d1
			n	–	m	27-d2	m	25-s1, 26-d2	m	28-d1	m	23-d2	m	40-i1
		Root	n	–	m	27-d2	m	25-d2	m	26-d3	m	23-d2	m	20-d1
			n	–	m	27-d2	m	25-s1, 26-d2	m	28-d1	m	23-d2	m	40-i1
SH#b14	10	Leaf	m	25-d2	n	–	m	25-d1	m	26-d1	m	22-d2	m	20-d2
			m	25-d2	n	–	m	25-d2	m	26-d1	m	26-d3	m	19-d2
		Root	m	25-d2	n	–	m	25-d1	m	26-d1	m	22-d2	m	20-d2
			m	25-d2	n	–	m	25-d2	m	26-d1	m	26-d3	m	19-d2
SH#c15	10	Leaf	m	25-d1	m	19-d2	n	–	m	26-d2	m	22-d1	m	20-d2
			m	40-i1	m	20-d2	n	–	m	40-i1	m	21-d2	m	20-d2
		Root	m	25-d1	m	19-d2	n	–	m	26-d2	m	22-d1	m	20-d2
			m	40-i1	m	20-d2	n	–	m	40-i1	m	21-d2	m	20-d2
SH#d16	10	Leaf	m	24-d1	m	26-d2	m	26-d2	n	–	m	22-d1	m	22-d2
			m	24-d1	m	26-d2, 28-s1	m	26-d2	n	–	m	22-d2	m	23-d2
		Root	m	24-d1	m	26-d2	m	26-d2	n	–	m	22-d1	m	22-d2
			m	24-d1	m	26-d2, 28-s1	m	26-d2	n	–	m	22-d2	m	23-d2
SH#e19	10	Leaf	m	27-d2	m	26-d2, 28-s1	m	25-d1	m	24-d1	n	–	m	22-d1
			m	27-d2	m	26-d2, 28-s1	m	24-d2	m	24-d1	n	–	m	22-d1
		Root	m	27-d2	m	26-d2, 28-s1	m	25-d1	m	24-d1	n	–	m	22-d1
			m	27-d2	m	26-d2, 28-s1	m	24-d2	m	24-d1	n	–	m	22-d1
SH#f20	10	Leaf	m	25-d1	m	19-d1	m	26-d1	m	26-d2	m	23-d1	n	–
			m	25-d1	m	19-d1	m	40-i1	m	26-d3	m	23-d1	n	–
		Root	m	25-d1	m	19-d1	m	26-d1	m	26-d2	m	23-d1	n	–
			m	25-d1	m	19-d1	m	40-i1	m	26-d3	m	23-d1	n	–
YS#16	12	Leaf	m	24-d1	m	19-d2	m	22-d2	m	25-d2	m	22-d1	m	22-d2
			m	24-d1	m	19-d2	m	22-d2	m	25-d2	m	22-d2	m	22-d2
		Root	m	24-d1	m	19-d2	m	22-d2	m	25-d2	m	22-d1	m	22-d2
			m	24-d1	m	19-d2	m	22-d2	m	25-d2	m	22-d2	m	22-d2
YS#a12	10	Leaf	n	–	m	16-d2, 18-s1	m	22-d2	m	25-d2, 27-s1	m	22-d1	m	23-d1
			n	–	m	16-d2, 18-s1	m	22-d2	m	24-d4	m	22-d1	m	23-d2
		Root	n	–	m	16-d2, 18-s1	m	22-d2	m	25-d2, 27-s1	m	22-d1	m	23-d1
			n	–	m	16-d2, 18-s1	m	22-d2	m	24-d4	m	22-d1	m	23-d2
YS#b13	10	Leaf	m	27-d1	n	–	m	22-d1	m	23-d1	m	21-d1	m	22-d2
			m	27-d2	n	–	m	22-d1	m	23-d1	m	21-d2	m	22-d2
		Root	m	27-d1	n	–	m	22-d1	m	23-d1	m	21-d1	m	22-d2
			m	27-d2	n	–	m	22-d1	m	23-d1	m	21-d2	m	22-d2
YS#c27	10	Leaf	m	23-d1	m	22-d1	n	–	m	23-d1	m	22-d2	m	23-d1
			m	23-d1	m	22-d2	n	–	m	40-i1	m	26-d3	m	22-d2
		Root	m	23-d1	m	22-d1	n	–	m	23-d1	m	22-d2	m	23-d1
			m	23-d1	m	22-d2	n	–	m	40-i1	m	26-d3	m	22-d2
YS#d28	10	Leaf	m	22-d1	m	23-d2	m	22-d2	n	–	m	24-d1	m	22-d1
			m	22-d2	m	22-d2, 24-s1	m	22-d2	n	–	m	24-d2	m	22-d1
		Root	m	22-d1	m	23-d2	m	22-d2	n	–	m	24-d1	m	22-d1
			m	22-d2	m	22-d2, 24-s1	m	22-d2	n	–	m	24-d2	m	22-d1
YS#e30	10	Leaf	m	27-d1	m	19-d2	m	22-d1	m	23-d2	n	–	m	24-d1
			m	40-i1	m	19-d2	m	22-d2	m	23-d2	n	–	m	24-d1
		Root	m	27-d1	m	19-d2	m	22-d1	m	23-d2	n	–	m	24-d1
			m	40-i1	m	19-d2	m	22-d2	m	23-d2	n	–	m	24-d1
YS#f32	10	Leaf	m	22-d1	m	19-d1	m	23-d1	m	22-d2	m	24-d1	n	–
			m	22-d1	m	19-d2	m	23-d2	m	22-d4	m	24-d1	n	–
		Root	m	22-d1	m	19-d1	m	23-d1	m	22-d2	m	24-d1	n	–
			m	22-d1	m	19-d2	m	23-d2	m	22-d4	m	24-d1	n	–

^a^SH: *CmDMC1*-edited lines from the cultivar ’Shuho-no-chikara’; YS: *CmDMC1*-edited lines from ‘Yamate-shiro’.

^b^The letters 'm' and 'n' indicate mutation and non-mutation, respectively, in the spacer region between the two TALEN binding sites.

^c^A number indicates the nucleotide position of TALEN-binding sites.

d# indicates the number of nucleotides deleted, i1 and s1 indicate a nucleotide insertion and substitution, respectively.

**Figure 3:**
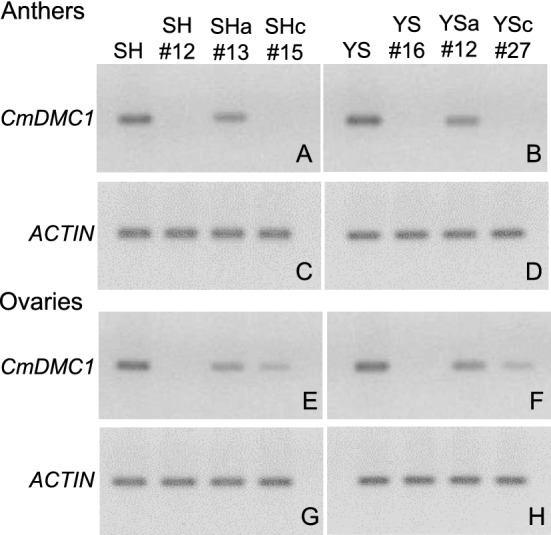
Expression levels of *CmDMC1* genes in *CmDMC1*-TALEN chrysanthemum plants. To detect endogenous *CmDMC1* transcripts, total RNA was isolated from anthers and ovaries at early meiotic division stage before tetrad formation of *CmDMC1*-TALEN and non-transgenic control plants grown at 20 °C. Twenty μg of total RNA was applied to each lane. The RNA blots were probed with a 1032-bp fragment of the *CmDMC1a* cDNA as in Supplementary Fig. S1, and 1134-bp of the *ACTIN* cDNA of *Ch. morifolium*. SH and YS: RNA from non-transgenic controls ‘Shuho-no-chikara’ and ‘Yamate-shiro’, respectively. SH#12, SHa#13 and SHc#15 in panels A, C, E and G: TALEN lines from ‘Shuho-no-chikara’, and YS#16, YSa#12 and YSc#27 in panels B, F, D and H: those from ‘Yamate-shiro’. Panels A, B, C and D show RNA blots of anthers and panels E, F, G and H show those of ovaries. Their full-length blots are presented in Supplementary Fig. 5.

Transgenic chrysanthemums carrying the TALENs construct for *CmDMC1* and non-transgenic chrysanthemums were grown at 20 °C and analyzed for *CmDMC1* transcripts in anthers and ovaries by northern blotting. The expected size of the *CmDMC1* mRNA was ca. 1.3 kbp. A strong hybridization signal was detected in the non-transgenic controls, while no signals were detected in anthers and ovaries of the transgenic lines carrying mutations in all 12 alleles (SH#12 and YS#16 in Fig. 3 and Supplementary Fig. S5). Lines SHc#15 and YSc#27 bore biallelic mutations in five *CmDMC1* genes including *CmDMC1a* and *CmDMC1b*, and showed no signals in anthers, but weak signals in ovaries. Lines SHa#13 and YSa#12 did not possess mutation except in *CmDMC1a*, and exhibited moderate signals in both anthers and ovaries (Fig. 3, Supplementary Fig. S5).

Growth characteristics of the transgenic lines SH#12 and YS#16 were compared to non-transgenic controls in a bio-safety containment greenhouse (hereinafter referred to as greenhouse). No obvious differences in growth and morphology were detected (Supplementary Table S7, Supplementary Fig. S6).

### Analysis of male sterility in *CmDMC1*-TALEN chrysanthemum plants

**Figure 4:**
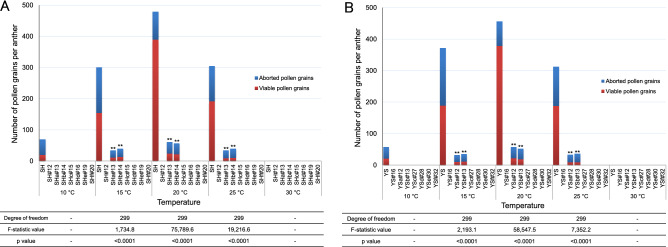
Evaluation of male sterility in *CmDMC1*-edited plants and non-transgenic controls by Alexander staining of pollen grains. (**A**) *CmDMC1*-edited plants of ‘Shuho-no-chikara’ and (**B**) *CmDMC1*-edited plants of ‘Yamate-shiro’. Red and blue bars indicate viable and aborted pollen grains, respectively. ** significant at 1% by ANOVA.

**Figure 5:**
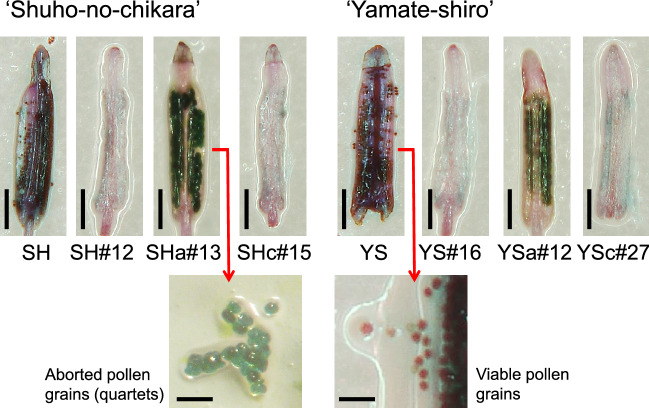
Assessment of pollen viability in *CmDMC1*-edited chrysanthemum plants. One day before flowering, anthers were stained according to Alexander^22^. Red- and green-colored pollen grains were judged to be viable and aborted, respectively. Scale bars indicate 0.2 mm (anthers) and 0.1 mm (magnified images of pollen at the center). SH and YS indicate non-transgenic controls ‘Shuho-no-chikara’ and ‘Yamate-shiro’. SH#12, SHa#13 and SHc#15 indicate *CmDMC1-*TALEN lines from ‘Shuho-no-chikara’, and YS#16, YSa#12 and YSc#27 from ‘Yamate-shiro’.

Tubular flowers were collected from the *CmDMC1*-TALEN lines and non-transgenic controls 1 day before flowering (Supplementary Fig. S7A, B) and stained by Alexander staining solution^22^ for analysis of male sterility (Figs. 4 and 5, Supplementary Table S8, Supplementary Fig. S8). No pollen grains were observed in anthers of the non-transgenic controls grown at 30 °C, whereas viable pollen grains were observed in anthers of non-transgenic controls at 10–25 °C. Rates of viable pollen grains in anthers of the *CmDMC1*-TALEN lines were evaluated according to Alexander’s method^22^. Rates of viable pollen grains in the controls at 20 °C were over 80%, but the rates declined to about 60% at 25 °C, 50% at 15 °C and 30–40% at 10 °C (Supplementary Table S8). No viable pollen grains were observed in the two *CmDMC1*-TALEN lines SH#12 and YS#16 with mutation in all the *CmDMC1* genes and without *CmDMC1* transcript at all temperature ranges tested. Two *CmDMC1*-TALEN lines, SHc#15 and YSc#27 bearing biallelic mutation in five genes including *CmDMC1a* and *CmDMC1b* (Table 1 and Supplementary Table S5), displayed no pollen grains at all temperature ranges tested (Fig. 4, Supplementary Table S8, Supplementary Fig. S8). In contrast, *CmDMC1*-TALEN lines SHa#13 and YSa#12, carrying biallelic mutations in five of the six genes with the exception of *CmDMC1a* (Table 1), produced viable pollen grains when grown at 15–25 °C (Figs. 4 and 5, Supplementary Fig. S8). Viable pollen produced by the non-transgenic control was somewhat sporadic. However, aborted pollen grains were observed more frequently than viable pollen grains in *CmDMC1*-TALEN lines such as SHa#13 and YSa#12. Aborted pollen grains seemed to be at the tetrad stage and were stuck to each other, so they looked larger than viable pollen. The rates of viable pollen in TALEN-mutated lines were significantly lower than those in non-transgenic controls according to ANOVA test.

### Analysis of female sterility in *CmDMC1*-TALEN chrysanthemum plants

**Figure 6:**
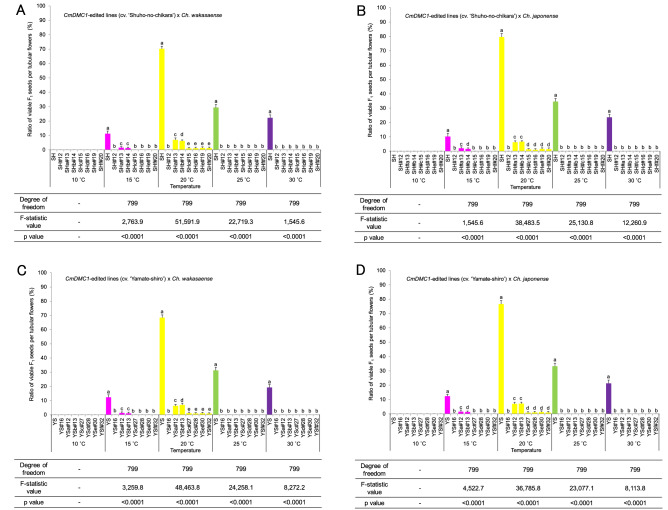
Evaluation of female sterility by crossing between transgenic lines and their wild relatives. Crossing between *CmDMC1*-TALEN plants of ‘Shuho-no-chikara’ and *Ch. wakasaense* (**A**) or *Ch. japonense* (**B**). Crossing between *CmDMC1*-edited plants of ‘Yamate-shiro’ and *Ch. wakasaense* (**C**) or *Ch. japonense* (**D**). Each value followed by the same letter is not significantly different at 5% level by Tukey–Kramer’s HSD.

Viable pollen grains from the wild relatives grown at 20 °C were collected and pollinated to stigmas of *CmDMC1*-TALEN lines and non-transgenic controls. In addition, these TALEN lines and non-transgenic controls were self-pollinated. Two types of achenes (F_1_ seeds) were observed 2 months after crossing and self-pollination (Supplementary Fig. S9). We evaluated the F_1_ seeds with germination ability as viable (A, C, G and I in Supplementary Fig. S9) and the ones without germination ability as aborted (B, D, E, F, H, J, K and L in Supplementary Fig. S9). Seed coats of the viable F_1_ seeds were hard and relatively bulgy, whereas the aborted F_1_ seeds were very small and fragile. The ratios of viable F_1_ seeds were 68.4–79.6% for non-transgenic controls that were grown, pollinated and matured at 20 °C, but these ratios declined to ca. 30% at 25 °C, 20% at 30 °C and 10% at 15 °C (Fig. 6, Supplementary Table S9). These results indicated that the ovules of non-transgenic controls are fertile. On the other hand, the two *CmDMC1*-TALEN lines SH#12 and YS#16 with biallelic mutations in all alleles gave no viable F_1_ seeds at any of the temperature ranges used for growth and pollination (Fig. 6, Supplementary Table S9), indicating that ovules of SH#12 and YS#16 are sterile. The ratios of viable F_1_ seeds were ca. 1%, and low levels of transcripts were detected by northern blot analysis of RNA from ovaries of *CmDMC1*-TALEN lines grown and pollinated at 20 °C with biallelic mutations in five *CmDMC1* genes including *CmDMC1a* and *CmDMC1b* (data for SHc#15 and YSc#27 in Fig. 3 and Supplementary Fig. S5). The four *CmDMC1*-TALEN lines SHa#13, SHb#14, YSa#12 and YSb#13 with biallelic mutations in five *CmDMC1* genes and without mutation in one of the two genes, *CmDMC1a* or *CmDMC1b* (Table 1), exhibited a ratio of viable F_1_ seeds of 1–2% when grown, pollinated and matured at 15 °C and about 6–7% at 20 °C (Supplementary Table S9), and intermediate levels of *CmDMC1* transcripts were detected by northern blot analysis (Fig. 3, Supplementary Fig. S5). The ratios of viable F_1_ seeds in the *CmDMC1*-TALEN lines were significantly lower than those in non-transgenic controls, according to Tukey–Kramer’s HSD test (Fig. 6, Supplementary Table S9).

Thus, our results indicate that the biallelic knockout by TALEN of six *CmDMC1* loci confers to reduce both female and male fertility on chrysanthemums over a wide range of flowering temperatures, including the optimum growth temperature for chrysanthemums, i.e., between 17 and 22°C^23^.

## Discussion

The introduction of targeted DNA double-strand breaks (DSBs) via sequence-specific nucleases (SSNs), such as meganucleases^24^, zinc finger nucleases (ZFNs)^25^, transcription activator-like effector nucleases (TALENs)^26^, and the bacterial clustered regularly interspaced short palindromic repeat (CRISPR)/CRISPR-associated protein 9 (Cas9) system^27^, results in deletions, insertions, and substitutions around the nuclease cleavage sites in the target genes. Recently, SSNs have become useful tools for genome engineering in plants, and these “genome editing” technologies have been established in many plants including *Arabidopsis*^28^, rice^29,30^, wheat^31^, soybean^32^, barley^33^, maize^34^, potato^35,36^, sugarcane^37^, etc.

Although the number of papers reporting successful genome editing in plants is increasing rapidly, there are still few reports mutating multi-alleles in highly polyploid plants using TALENs. For example, bread wheat (*Triticum aestivum*) is a hexaploid with the genomic constitution AABBDD (2*n* = 6x = 42) in which each constituent subgenome originated from a different ancestral species. Allopolyploidization leads to the generation of duplicated homoeologous genes (homoeologs) and, consequently, the hexaploid wheat genome contains triplicated homoeologs derived from the three ancestral diploid species. Wang et al.^31^ succeeded in mutating all three sets of homoeoalleles for *MILDEW-RESISTANCE LOCUS* (*MLO*) to produce wheat resistant to powdery mildew. Cultivated potato (*Solanum tuberosum*) is a highly heterozygous autotetraploid (AAAA, 2*n* = 4x = 48). Disruption of the gene for sterol side chain reductase (*SSR2*) in potatoes resulted in low SSR2 activity and a reduction in the levels of two toxic steroidal glycoalkaloids, α-solanine and α-chaconine^35,36^. Modern sugarcane varieties are complex interspecific hybrids (*Saccharum* spp.) and highly heterozygous allopolyploid ranging from decaploid to tridecaploid (2*n* = 10 × to 13x = 100–130). To reduce lignin content and improve biofuel yield, Jung and Altpeter^37^ induced mutations by TALENs in the gene for *caffeic acid O-methyltransferase* (*COMT*), involved in lignin biosynthesis.

Chrysanthemums are hexaploids (2*n* = 6x = 54) with a loss or gain of several chromosomes^1^, originated by crossing and duplicating between *Ch. zawadskii* var. *latilobum* (Maxim.) Kitamura (2*n* = 2x = 18) and *Ch. indicum* var. *procumbense* (Lour.) Kitamura (2*n* = 4x = 36)^38^. Very recently, Kishi-Kaboshi et al.^39^ succeeded in knocking out multiple transgenes previously integrated in transgenic chrysanthemums using the CRISPR/Cas9 system. They used transgenic chrysanthemum lines harboring more than four or five copies of a gene for yellow-green fluorescent protein from *Chiridius poppei* (*CpYGFP*) as materials for gene disruption, and edited all the *CpYGFP*-transgene alleles simultaneously. In this study, we isolated six *CmDMC1* loci and intended to mutagenize the BRC multimer interface just upstream of the Walker B motif^20^, since Walker A and/or B motifs are functionally essential ATP-binding sites^40^. Disruption of these motifs by frameshift mutations could destroy DMC1 function, resulting in abnormal meiosis and, consequently, a male and female sterility phenotype. Interestingly, changing even one amino acid at these motifs caused ablation of ATP binding and inactivation of human DMC1^41,42^. Similarly, Dresser et al.^43^ and Masson and West^44^ reported that a *Saccharomyces cerevisiae* DMC1 mutant carrying a single amino acid substitution in the ATP-binding site confers a null mutation.

Previous studies have indicated that the sizes of the INDELs mediated by TALENs are mostly less than 300 bp including the TALEN target sites^28–37^. We designed a pair of primers generating amplicons of about 300 bp including the TALEN target sites for *CmDMC1* genes to identify deletions in the sites in addition to various insertions and substitutions. Using TALEN-mediated targeted mutagenesis of six *CmDMC1* loci, three patterns of mutation were induced. The frequency of monoallelic mutations was lower than that of biallelic mutations. Moreover, biallelic mutations carrying the same mutation in both alleles of a *CmDMC1* gene were detected with approximately the same frequency as biallelic mutations carrying different mutations in two alleles of the gene. In calli of rice cultivars, most of the TALEN-induced deletion mutations at *Waxy* locus were less than 100 bp, and deletions larger than 300 bp occurred rarely^30^. The largest deletion observed in *SSR2*-TALEN lines of tetraploid potato was 283 bp^36^. Accordingly, sequences of larger regions (~ 1.8 kbp) surrounding the TALEN target sites for *CmDMC1* loci were analyzed in the TALEN-induced mutant lines of chrysanthemum. The sequencing results detected no novel mutations outside the target site of *CmDMC1a* locus in each TALEN lines, indicating only small INDELs and substitutions could occur in our system (Table 1, Supplementary Table S5 and Supplementary Fig. S4).

In our TALEN-mediated editing of *CmDMC1*, both in-frame mutations conferring insertion or deletion of amino acid(s) and frameshift mutations were detected. Looking for putative *CmDMC1* products, we noted that the frameshift mutants could generate premature termination codons (PTCs) just downstream of the mutated sites (Supplementary Table S6). Northern blot analysis of *CmDMC1* transcripts detected markedly decreased or no hybridization signals in four out of six *CmDMC1*-TALEN lines: SH#12, SHc#15, YS#16 and YSc#27 (Fig. 3 and Supplementary Fig. S5) bearing frameshift mutations in at least the *CmDMC1a* and *CmDMC1b* loci. All the frameshift mutations found in those lines potentially generate aberrant mRNAs carrying PTCs that would be recognized and rapidly degraded by nonsense-mediated mRNA decay (NMD). NMD, which is conserved among eukaryotes, is a mechanism of quantity and quality control of mRNAs that prevents accumulation of detrimental truncated proteins^45,46^. Regulation of NMD activity plays a crucial role in plant growth and responses to the environment^47,48^.

In contrast, the allele harboring a deletion of three nucleotides potentially generates deletion of a single amino acid in the respective DMC1 proteins encoded by a single allele of *CmDMC1d* (lines SHa#13 and SHf#20) or *CmDMC1e* (SHb#14 and YSc#27). In these four lines, each CmDMC1d or CmDMC1e mutant protein carries a deletion of Lys at position 215 (K215) located in the BRC multimer interface domain, which was proposed to be involved in monomer–monomer interaction^20^. This Lys residue—in equivalent positions in DMC1 proteins—is shared among mammals and higher plants^49^ that bear both *BRCA* and *DMC1* genes. Deletion of such a positively charged amino acid at the interface domain might affect DMC1–DMC1 and/or DMC1–BRCA2 interactions^50–53^.

The male and female sterility of the 14 *CmDMC1*-TALEN lines bearing biallelic mutations in five or all six *CmDMC1* genes differed significantly from non-transgenic controls. In fact, rates of male and female sterility seem to depend on the mutated *CmDMC1* locus. Biallelic mutations of all *CmDMC1* genes or both *CmDMC1a* and *CmDMC1b* genes caused complete male sterility at 10–30 °C. However, when one of the two genes was not mutated, mature pollen grains were formed at 15–25 °C (Supplementary Table S8, Supplementary Fig. S8).

When *CmDMC1a* or *CmDMC1b* was not mutated in *CmDMC1*-TALEN lines as the female parents, viable F_1_ seeds with the ability to germinate were formed at 15 and 20 °C. When any one of the *CmDMC1c*, *CmDMC1d*, *CmDMC1e* or *CmDMC1f* genes was not mutated in the female parents, ratios of viable F_1_ seeds were much lower only at 20 °C (Fig. 6, Supplementary Table S9). These results showed that female fertility is influenced strongly by *CmDMC1a* and/or *CmDMC1b*, and moderately by *CmDMC1c*, *CmDMC1d*, *CmDMC1e* and/or *CmDMC1f*, and that loss-of-function mutations in all the *CmDMC1* loci are required for the loss of crossing ability in female reproductive organs that is independent of ambient temperature. As a future study, microscopic observation of egg cells from the *CmDMC1*-TALEN lines would be necessary to verify the effects of *CmDMC1* mutagenesis by genome editing.

The genes for TALENs and the *nptII* selection marker were driven by a bidirectional promoter from the *mannopine synthase-2′* and *-1′* (*mas2′-1′*) genes of an *A. tumefaciens* strain as pathogen of the Compositae family. Mannopine synthase is composed of two enzyme conjugates encoded by the *mas1′* gene and a reductase encoded by the *mas2′* gene^54^. These two genes are located on the T-DNAs of certain octopine-type Ti and Ri plasmids^55,56^, and the *mas2′* and *mas1′* promoters are oriented in a head-to-head manner on a 483-bp fragment of pTiAch5^55^. The *mas2′-1′* promoter conferred high level of expression to the modified *cry1Ab* gene from *Bacillus thuringiensis*, the *nptII* gene and the *CmDMC1a*-RNAi construct in leaves, stems, roots and pollen, and weaker expression levels in ovaries^18^. The difference in the degree of sterility between male (pollen) and female (ovaries) in *CmDMC1a*-RNAi chrysanthemums might be due to the differential activity of the promoter controlling RNAi expression in the organs. The *CmDMC1*-TALEN lines were all mutated at the target sequence, and 5 of 23 regenerated plants in ‘Shuho-no-chikara’ and 2 of 126 regenerated plants in ’Yamate-shiro’ were mutated in all six loci (Supplementary Table S4). According to DNA sequence analysis of the six *CmDMC1* loci, these seven lines showed one or two mutation patterns without wild-type sequences at each *CmDMC1* locus (ex. SH#12 and YS#16 in Table 1, Supplementary Tables S5 and S6). The frequency of biallelic mutations in the transgenic plants was much higher than that reported in other polyploidy plants mutagenized by TALENs^31,35–37^. This seemed to be due to the use of a *mas2′-1′* promoter, whose expression is relatively high at the site of *Agrobacterium* infection in leaves and in calluses of chrysanthemum. Thus, a stable male and female sterility phenotype could be introduced into chrysanthemum by the proper selection of a TALENs target sequence that is highly conserved among *CmDMC1* multi-genes, and by the use of a *mas2′-1′* promoter that can express TALENs strongly in chrysanthemum.

In the genetic transformation of chrysanthemum, transgenic plants with chimeric nature were reported. Early studies, that used direct plant regeneration from leaf or stem segments, often showed chimeric nature in the transgenic plants^57,58^. Transgenic plants generated from somatic embryos, possibly with the single transgenic-cell origin, displayed non-chimerism^59^. However, number of cultivars suitable for somatic embryogenesis is limited^60^, and the regeneration system through somatic embryogenesis would not be widely applicable for chrysanthemum transformation. We circumvented the chimerism using a callus induction and regeneration system^61^ (Supplementary Table S3). By applying relatively strict antibiotic selection under 20 mg l^−1^ G418 for 2.5 to 3 months during callus formation. Non-transformed calli (escapes) were effectively eliminated and only transformed calli survived on the medium. The formation of chimeras may also occur during the process of producing genome-edited plants^62,63^. We obtained *CmDMC1*-TALEN chrysanthemum plants according to the above-mentioned system, and analyzed partial DNA sequences of the six *CmDMC1* genes for over 13 individual clones bearing genomic PCR products surrounding TALEN recognition sites (about 300 bp and 1.8 kbp) amplified from total DNAs of leaves or roots of the TALEN lines. The results indicated that no more than two mutation patterns were detected for each *CmDMC1* locus (Supplementary Table S5) and that no other mutations were detected outside the target sites for each *CmDMC1a* locus (Supplementary Fig. S4). Though these results support the non-chimeric nature of our *CmDMC1*-TALEN lines, more detailed studies would be needed for further confirmation such as the elucidation of the relationship between mutation patterns in the six *CmDMC1* genes (genotypes) and sterility phenotypes in the clonal plants.

Since chrysanthemum exhibits self-incompatibility, it is hard to obtain null segregants by self-fertilizaion. Recently, DNA-free genome editing methods have been developed in potato^64^ and bread wheat^65^. It is expected that DNA-free methods will be more advantageous for environmental safety assessments than DNA-based methods introducing transgene(s) into the genome.

In this study, we determined six *CmDMC1* cDNA sequences and six corresponding partial genomic DNA fragments from 10 chrysanthemum cultivars. However, because some chrysanthemum cultivars have unstable and variable chromosome numbers that form a hexaploid complex with aneuploidy (2*n* = 6x = 54 ± 7–10)^1^, a search for other *CmDMC1* loci from other cultivars or Compositae family will be needed, as well as an analysis of allelic configurations, including these six loci, to assess the efficiency of mutagenesis by genome editing in the prevention of transgene flow.

The strategy reported here should be useful in preventing transgene flow via pollen. A stable sterility phenotype will be a key technology for the practical use of chrysanthemums transgenic for characteristics such as pest/disease resistance and flower color modifications under the terms of the Cartagena Protocol on Biosafety.

## Methods

### Plant materials and culture conditions

Ten chrysanthemum (*Ch. morifolium* Ramat.) cultivars (double flower type: ‘Shuho-no-chikara’, ‘Seiun’, ‘Shinba No. 2’ and ‘Summer Yellow’; single flower type: ‘Yamate-shiro’, ‘Kosuzu’, ‘Utage’, ‘Monroe’, ‘Kofuku-no-tori’ and ‘Kin-fusha’) were used. Shoot tips of plants grown in a greenhouse were surface-sterilized in 70% ethanol and then in a 1% sodium hypochlorite solution for 15 min. They were rinsed three times with sterile distilled water. Shoot tip explants were cultured in vitro (meristem culture) on Murashige and Skoog (MS) basal medium^66^ containing 3% sucrose and 0.3% gellan gum (FUJUFILM Wako Pure Chemical Corporation, Osaka, Japan). The medium was adjusted to pH 5.8 before autoclaving at 121 °C for 15 min. The explants were cultured at 25 °C under a 16-h photoperiod using cool white fluorescent lamps or at 25 °C in darkness. The lamps provided a photosynthetic photon flux density (PPFD between 400 and 700 nm) of 60 μmol m^−2^ s^−1^.

### Isolation of *CmDMC1* loci from chrysanthemum cultivars

The 10 chrysanthemum cultivars described above were used. In a previous study^18^, we cloned a partial cDNA encoding a DMC1 protein from chrysanthemum cultivar ‘Shuho-no-chikara’. Based on this sequence, we isolated cDNAs encoding DMC1 homologs from ‘Shuho-no-chikara’ and the other nine cultivars using a SMART RACE cDNA Amplification Kit (Clontech Laboratories, Mountain View, CA, USA) and cDNA libraries of individual cultivars (Supplementary Fig. S1). Partial DNA fragments of individual *CmDMC1* genes that correspond to cDNA nucleotide positions 234–815 of ‘Shuho-no-chikara’ (Supplementary Fig. S1) were amplified from genomic DNAs (Fig. 1) using the primer pair DMC1-RNAi F1 (5′-aatctgtgaagctgct-3′) and DMC1-RNAi R1 (5′-tttgtcatatacac-3′) (Fig. 1). Multiple sequence alignment of the conserved core regions of *CmDMC1* genes was performed using DNASIS software version 3.7 (Hitachi Software Engineering, Tokyo, Japan). The target sequence for TAL effector repeat array (TALEN recognition sequence 5′ upstream: TAL-L; TALEN recognition sequence 3′ downstream: TAL-R) was designed using tools from the TAL Effector Nucleotide Targeter 2.0^67^ for multigene disruption (Fig. 1). Sequences of 48 clones per cDNA and genomic DNA (over 7 clones per cDNA and genomic DNA sequence) were analyzed for isolation of *CmDMC1* loci.

### Plasmid construction

The TAL effector repeat array was synthesized using a Golden Gate TALEN and TAL Effector Kit 2.0 (addgene, Cambridge, MA, USA). The TALEN pair targeting the *CmDMC1* homologs was cloned into the binary vector pBIK201G^10^ by substituting the *β*-d-*glucuronidase A* (*gusA*) gene cassette. The TALEN-pair segment and the *nptII* gene encoding neomycin phosphotransferase II as selectable marker were placed under the control of bi-directional promoters from the *mannopine synthase 1*′ and *2*′ (*mas1*′*-2*′) genes. The resulting vector, designated ‘pBIK201DMC-TAL’ (Fig. 2), was introduced into *A. tumefaciens* strain EHA105^21^, kindly provided by Dr. L. S. Melchers.

### Plant transformation

Two popular cultivars, ‘Shuho-no-shikara’ and ‘Yamate-shiro,’ which demonstrated high transformation rates^61^, were used. Preparation of *Agrobacterium* culture, cocultivation, selection of G418-resistant calluses, and plant regeneration were performed as described in Supplementary Table S3 and Shinoyama et al.^61^.

### Southern blot hybridization

Total DNA was extracted from 100 mg of fresh young leaves of *CmDMC1*-TALEN lines and non-transgenic control plants according to Shinoyama et al.^61^. A 25-μg aliquot of DNA digested with *Xba*I was subjected to electrophoresis and blotted onto a Hybond N^+^ nylon membrane (GE Healthcare, Tokyo, Japan). Southern blot hybridization^68^ was carried out using a digoxigenin (DIG)-labeled *nptII* fragment (about 800 bp) as a probe (Fig. 2) and a DIG DNA Labeling and Detection Kit (Roche Diagnostics, Mannheim, Germany) according to the supplier’s instructions.

### Sequencing of genomic DNA from transgenic lines

Total DNAs were prepared from leaves and roots of *CmDMC1*-TALEN lines as described in Shinoyama et al.^61^. At first, fragments of about 300 bp, including the TALEN recognition sequences (TAL-L and TAL-R; Fig. 1) for *CmDMC1* genes, were amplified by PCR using a total DNA preparation as a template, ExTaq DNA polymerase (Takara-Bio, Shiga, Japan) and primer pair DMC1-gF1 (5′-ggcatggatcctggagctgtac-3′) and DMC1-gR1 (5′-ctgcaagttctcctcttccagtg-3′) (Fig. 1). Fragments of about 1.8 kbp including the 300 bp sequence were amplified by PCR using total DNA preparations as templates and primer pair DMC1-RNAi F1 (5′-aatctgtgaagctgct-3′) and DMC1-RNAi R1 (5′-tttgtcatatacac-3′) (Fig. 1). The amplicon DNA was ligated to a T-vector (pMD20, Takara-Bio) and transformed into *Escherichia coli* JM109. Sequences of the individual inserts were read by M13 primers M4 or RV using an ABI PRISM 3100 Genetic Analyzer with POP-4 polymer and an 80-cm capillary array (Thermo Fisher Scientific, Waltham, MA, USA). Sequences of over 13 clones per locus were analyzed for each *CmDMC1*-TALEN line.

### Northern blot hybridization

Total RNA was extracted from 150 mg fresh weight of anthers and ovaries of *CmDMC1*-TALEN lines and a non-transgenic control grown at 20 °C. Each sample was homogenized in liquid nitrogen using a ceramic mortar and pestle. Total RNA was prepared from the homogenate using an RNeasy Plant Mini Kit (Qiagen, Hilden, Germany). A 20-μg aliquot of RNA was fractionated by formaldehyde gel electrophoresis through 0.8% agarose containing ethidium bromide in MOPS buffer (20 mM MOPS, 5 mM sodium acetate, 1 mM EDTA, pH 7.0). Equal loading was confirmed by examining the gel with ultraviolet light. The gel was then blotted onto a nylon membrane (Nylon Membranes, positively charged; Roche Diagnostics). Prehybridization, hybridization and detection conditions were as described above for Southern blot hybridization^68^ using a *CmDMC1a* cDNA fragment (1032 bp, Supplementary Fig. S1) and an *ACTIN* gene fragment (1134 bp, accession number: LC576411) from *Ch. morifolium* as probes.

### Analysis of growth characteristics of *CmDMC1*-TALEN and non-transgenic chrysanthemum plants

*CmDMC1*-TALEN lines bearing biallelic mutations in all (six) or five *CmDMC1* genes and a non-transgenic control were acclimatized in a greenhouse at 20 °C and propagated by using stem cuttings in vermiculite. After rooting, these plants (height ~ 5 cm) were exposed to 10 °C for 40 days for vernalization under a 16-h photoperiod and cool white fluorescent lamps at a PPFD (400–700 nm) of 200 μmol m^–2^ s^–1^ in a temperature gradient incubator (TG-100-A, Nippon Medical & Chemical Instruments, Osaka, Japan). They were then cultivated in the greenhouse at 20 °C under natural daylength. After flowering, factors related to growth characteristics such as stem length and number of leaves were measured. Ten plants per line were used for the observation and measurements.

### Evaluation of male sterility in *CmDMC1*-TALEN and non-transgenic chrysanthemum plants

Male sterility was judged by pollen viability using a stain technology developed by Alexander^22^. *CmDMC1*-TALEN lines bearing biallelic mutations in all (six) or five *CmDMC1* genes and a non-transgenic control were cultivated in the greenhouse at 20 °C under natural daylength. At the early meiotic division stage before tetrad formation (when diameters of flower buds were about 6 mm), the plants were transferred to the temperature gradient incubator and flowered at temperatures ranging from 10 to 30 °C with an 8-h photoperiod. Tubular florets were separated from the head flower just before flowering (Supplementary Fig. S7A) and immersed in staining solution. The tubular florets were incubated in Alexander solution^22^ at 50 °C overnight. Anthers were cut from the tubular florets using a scalpel and observed with a digital microscope (RH-2000, HiROX, Tokyo, Japan). Numbers of viable pollen grains (stained red with Alexander solution) and aborted pollen grains (stained green) were then counted. The percentage of pollen viability was calculated as the rate of viable grains to total pollen grains. A total of 100 head flowers each and about 10 receptive tubular florets from each head flower were used for staining of the *CmDMC1*-TALEN and non-transgenic control plants of ‘Shuho-no-chikara’ and ‘Yamate-shiro’. All pollen grains from each anther (a tubular floret has five anthers) were counted.

### Evaluation of female sterility in *CmDMC1*-TALEN and non-transgenic chrysanthemums plants

*CmDMC1*-TALEN and non-transgenic plants were crossed with two wild relatives, *Ch. wakasaense* and *Ch. japonense*, kindly supplied by Dr. K. Taniguchi, Hiroshima University with National BioResource Project (NBRP) Chrysanthemum (https://shigen.nig.ac.jp/chrysanthemum/). The cultivar ‘Yamate-shiro’ is a quantitative short-day plant and flowers in August, whereas ‘Shuho-no-chikara’ and its wild relatives are qualitative short-day plants and flower October to November under natural daylength in Japan. To adjust the timing of flowering, *CmDMC1*-TALEN and non-transgenic plants and the wild relatives were exposed in June to appropriate low-temperature treatment at 10 °C for 40 days under a 16-h photoperiod and cool white fluorescent lamps (200 μmol m^−2^ s^−1^) in the temperature gradient incubator. *CmDMC1*-TALEN and non-transgenic plants of ’Shuho-no-chikara’ and ‘Yamate-shiro’ were then cultivated in the greenhouse at 20 °C under natural daylength. When flower buds of the *CmDMC1*-TALEN and non-transgenic control plants grew to a diameter of 6 mm at the early meiotic division stage, they were transferred to the temperature gradient incubator at temperatures ranging between 10 and 30 °C with an 8-h photoperiod. The pollen parents (wild relatives) were grown in the greenhouse at 20 °C, and pollen grains were collected from the dehiscent anthers of tubular florets using a small brush and placed into a Petri dish (60 mm in diameter). For the seed parents (*CmDMC1*-TALEN lines and non-transgenic controls), immature tubular florets were removed from head flowers with protruding stigmas; 100 flowers each and about 10 receptive stigmas each (the top of a stigma is Y-shaped as shown in Supplementary Fig. S7C) from a head flower were used for the *CmDMC1*-TALEN and non-transgenic control plants of ‘Shuho-no-chikara’ and Yamate-shiro’. The collected pollen grains were placed immediately on stigmas using a small brush, and each flower was covered with a paper bag. The flowers were then grown in an incubator (temperature range: 10–30 °C). After 2 months, all achenes (F_1_ hybrid seeds) were collected from the seed parents, sown on vermiculite and then incubated in the greenhouse at 20 °C. After 2 weeks, numbers of germinated and non-germinated seeds were counted, and assessed as viable and aborted, respectively.

### Statistical comparison of *CmDMC1*-TALEN and non-transgenic chrysanthemums plants

For analysis of male sterility, the average percentages of viable and non-viable pollen in each head flower were arcsine-transformed prior to analysis by ANOVA. For analysis of female sterility, the percentages of F_1_ seed production in each head flower were arcsine-transformed prior to analysis by Tukey–Kramer’s HSD.

## Supplementary information

Supplementary Information.

## Data Availability

All data generated or analyzed during this study are available from the corresponding author on reasonable request.
