# Supplementary material for: Simultaneous TALEN-mediated knockout of chrysanthemum DMC1 genes confers male and female sterility
Source: Sci Rep. 2020 Sep 30;10:16165. doi: 10.1038/s41598-020-72356-1 (PMC7527520; doi:10.1038/s41598-020-72356-1)
Supplement: Supplementary file 1 — Supplementary Information. [file 41598_2020_72356_MOESM1_ESM.pdf]

Simultaneous TALEN-mediated knockout of chrysanthemum *DMC1* genes confers male and female sterility

Harue Shinoyama[1]*, Hiroaki Ichikawa[2], Ayako Nishizawa-Yokoi[2,3], Mikhail Skaptsov[4] & Seiichi Toki[2,5,6]

[1]Fukui Agricultural Experiment Station, Fukui 918-8215, Japan

[2]Institute of Agrobiological Sciences, National Agriculture and Food research Organization (NARO), Tsukuba 305-8604, Japan

[3]Precursory Research for Embryonic Science and Technology (PRESTO), Japan Science and Technology Agency (JST), Saitama 332-0012, Japan

[4]South Siberian Botanical Garden, Altai State University, Barnaul 656049, Russia

[5]Graduate School of Nanobioscience, Yokohama City University, Yokohama 236-0027, Japan

[6]Kihara Institute for Biological Research, Yokohama City University, Yokohama 244-0813, Japan

*Corresponding author

Current affiliation: Department of Bioscience, Fukui Prefectural University, Awara, 910-4103, Japan.

Current E mail address: halshino@fpu.ac.jp

Supplementary Table S1. Presence of six *CmDMC1* cDNAs in 10 chrysanthemum cultivars.

cDNA	Cultivar with doubled flower types				Cultivar with single flower types					
	Shuho-no-chikara	Seiun	Shinba No. 2	Summer Yellow	Yamate-shiro	Kosuzu	Utage	Monroe	Kofuku-no-tori	Kin-fusha
CmDMC1a	+	+	+	+	+	+	+	+	+	+
CmDMC1b	+	+	+	+	+	+	+	+	+	+
CmDMC1c	+	+	+	+	+	+	+	+	+	+
CmDMC1d	+	+	+	+	+	+	+	+	+	+
CmDMC1e	+	+	+	+	+	+	+	+	+	+
CmDMC1f	+	+	+	+	+	+	+	+	+	+

Supplementary Table S2. DNA sequences of TALENs target site for *CmDMC1* .

TALEN recognition sequence 5' upstream (1-19)	spacer sequence (20-36)	TALEN recognition sequence 3' downstream (37-52)
ACAACCTGCTCCTTGGTTT	ggcagcaaagatggctg	AAGAACCTTTCAGACT

TALEN recognition sequences and a spacer sequence are shown in upper and lower cases, respectively.

Numbers in parentheses indicate nucleotide positions of TALENs target site for mutation analysis.

Supplementary Table S3. The procedure for chrysanthemum transformation.

Day	Procedure
-3	Pre-culture *Agrobacterium* on solid AB medium containing Kanamycin (50 mg l^{-1}).<br>Draw 8 cm line by platinum loop on petri-dish (φ = 10 cm) and incubated 3 days at 28 °C.
0	Scrape 8-cm line of *Agrobacterium* and culture in liquid YEP medium without antibiotics for 5 hour.<br>Immerse leaf discs into MS liquid medium containing *Agrobacterium* ($O.D._{660}$ = 0.1 to 0.2) for 30 min.<br>Cocultivate leaf discs with *Agrobacterium* on cocultivation medium for 3 days.
3	Transfer leaf discs to bacteria elimination medium.
10	Transfer leaf discs to selection medium I (selection of putatively transformed cells).
24	Transfer leaf discs to fresh selection medium I.
38	Transfer leaf discs to fresh selection medium I<br>(Induction of G418-resistant callus induction on the of leaf segments).
52	Transfer leaf discs to selection medium II.
66	Transfer leaf discs to fresh selection medium II.
80	Transfer leaf discs to plantlet regeneration medium.
101<br>122	Transfer leaf discs to fresh plantlet regeneration medium.<br>Transfer leaf discs to fresh plantlet regeneration medium (shoot regeneration).<br>Collect elongating shoots (first collection) and transfer to rooting medium.
143	Transfer leaf discs to fresh plantlet regeneration medium.<br>Collect elongating shoots (second collection) and transfer to rooting medium.
143 - 180	Transfer rooted plantlets to closed greenhouse.
200 onwards	Plants available for evaluation and analysis.

This table is a partial modification of Shinoyama et al.[61].

Ingredients for media

Cocultivation medium: MS + 1.0 mg l^{-1} NAA, 0.5 mg l^{-1} BA, 1.0 g l^{-1} Casamino acids, 3% Sucrose (Suc), 0.3% Gellan Gum (Gel)

Bacteria elimination medium: MS + 1.0 mg l^{-1} NAA, 0.5 mg l^{-1} BA, 3% Suc, 0.3% Gel, 250 mg l^{-1} Cefotaxime sodium salt (Cef)

Selection medium I: MS + 1.0 mg l^{-1} NAA, 0.5 mg l^{-1} BA, 3% Suc, 0.3% Gel, 250 mg l^{-1} Cef, 20 mg l^{-1} G418

Selection medium II: MS + 1.0 mg l^{-1} NAA, 0.5 mg l^{-1} BA, 3% Suc, 0.3% Gel, 100 mg l^{-1} Cef, 20 mg l^{-1} G418

Plantlet regeneration medium: MS + 0.5 mg l^{-1} BA, 0.2 mg l^{-1} GA_3, 3% Suc, 0.4% Gel, 100 mg l^{-1} Cef

Rooting medium: MS + 3% Suc, 0.4% Gel, 100 mg l^{-1} Cef

Supplementary Table S4. Transformation frequencies and mutation patterns in respective *CmDMC1* loci of chrysanthemum cultivars.

Cultivar	No. of infected leaf discs (L)	No. of regenerants	No. of transformants (T)	Transformation frequency (T/L) x 100 (%)	No. of mutated loci													
					6		5		4		3		2		1		0	
					A	B	A	B	A	B	A	B	A	B	A	B	A	B
Shuho-no-chikara	719	23	23	3.2	5	1	7	0	3	0	2	0	2	1	2	0	0	0
Yamate-shiro	720	126	126	17.5	2	0	12	1	17	1	20	2	35	2	32	2	0	0

A: a biallelic mutation was detected in each locus. B: a monoallelic mutation was detected in each locus.

Supplementary Table S5. Patterns of mutations in *CmDMC1* genes via TALENs.

Line<br>*CmDMC1* genotype	Gene	Nucleotide sequence	Mutated nucleotide position and its mutation type
Shuho-no-chikara (Control: SH)<br>*AABBCCDDEEFF*		ACAACCTGCTCCTTGGTTTggcagcaaagatggctgAAGAACCTTTCAGACT	
SH#12<br>*aabbccddeeff*	*CmDMC1a*	ACAACCTGCTCCTTGGTTTggcag-aaagatggctgAAGAACCTTTCAGACT	25-d1
		ACAACCTGCTCCTTGGTTTggcag-aaagatggctgAAGAACCTTTCAGACT	25-d1
	CmDMC1b	ACAACCTGCTCCTTGGTT-ggcagcaaagatggctgAAGAACCTTTCAGACT	19-d1
		ACAACCTGCTCCTTGGTT-ggcagcaaagatggctgAAGAACCTTTCAGACT	19-d1
	CmDMC1c	ACAACCTGCTCCTTGGTTTggcag--aagatggctgAAGAACCTTTCAGACT	25-d2
		ACAACCTGCTCCTTGGTTTggcagcaaagatggctgAAGGAACCTTTCAGACT	40-i1
	CmDMC1d	ACAACCTGCTCCTTGGTTTggcagc-aagatggctgAAGAACCTTTCAGACT	26-d1
		ACAACCTGCTCCTTGGTTTggcagcaaagatggctgAAGGAACCTTTCAGACT	40-i1
	CmDMC1e	ACAACCTGCTCCTTGGTTTgg-agcaaagatggctgAAGAACCTTTCAGACT	22-d1
		ACAACCTGCTCCTTGGTTTgg-agcaaagatggctgAAGAACCTTTCAGACT	22-d1
	CmDMC1f	ACAACCTGCTCCTTGGTTT-gcagcaaagatggctgAAGAACCTTTCAGACT	20-d1
		ACAACCTGCTCCTTGGTTT-gcagcaaagatggctgAAGAACCTTTCAGACT	20-d1
SHa#13<br>*AAbbccddeeff*	*CmDMC1a*	ACAACCTGCTCCTTGGTTTggcagcaaagatggctgAAGAACCTTTCAGACT	no mutation
		ACAACCTGCTCCTTGGTTTggcagcaaagatggctgAAGAACCTTTCAGACT	no mutation
	CmDMC1b	ACAACCTGCTCCTTGGTTTggcagca--gatggctgAAGAACCTTTCAGACT	27-d2
		ACAACCTGCTCCTTGGTTTggcagca--gatggctgAAGAACCTTTCAGACT	27-d2
	CmDMC1c	ACAACCTGCTCCTTGGTTTggcag--aagatggctgAAGAACCTTTCAGACT	25-d2
		ACAACCTGCTCCTTGGTTTggcagt--agatggctgAAGAACCTTTCAGACT	25-s1, 26-d2
	CmDMC1d	ACAACCTGCTCCTTGGTTTggcagc---gatggctgAAGAACCTTTCAGACT	26-d3
		ACAACCTGCTCCTTGGTTTggcagcaa-gatggctgAAGAACCTTTCAGACT	28-d1
	CmDMC1e	ACAACCTGCTCCTTGGTTTggc--caaagatggctgAAGAACCTTTCAGACT	23-d2
		ACAACCTGCTCCTTGGTTTggc--caaagatggctgAAGAACCTTTCAGACT	23-d2
	CmDMC1f	ACAACCTGCTCCTTGGTTT-gcagcaaagatggctgAAGAACCTTTCAGACT	20-d1
		ACAACCTGCTCCTTGGTTTggcagcaaagatggctgAAGGAACCTTTCAGACT	40-i1
SHb#14<br>*aaBBccddeeff*	*CmDMC1a*	ACAACCTGCTCCTTGGTTTggcag--aagatggctgAAGAACCTTTCAGACT	25-d2
		ACAACCTGCTCCTTGGTTTggcag--aagatggctgAAGAACCTTTCAGACT	25-d2
	CmDMC1b	ACAACCTGCTCCTTGGTTTggcagcaaagatggctgAAGAACCTTTCAGACT	no mutation
		ACAACCTGCTCCTTGGTTTggcagcaaagatggctgAAGAACCTTTCAGACT	no mutation
	CmDMC1c	ACAACCTGCTCCTTGGTTTggcag-aaagatggctgAAGAACCTTTCAGACT	25-d1
		ACAACCTGCTCCTTGGTTTggcag--aagatggctgAAGAACCTTTCAGACT	25-d2
	CmDMC1d	ACAACCTGCTCCTTGGTTTggcagc-aagatggctgAAGAACCTTTCAGACT	26-d1
		ACAACCTGCTCCTTGGTTTggcagc-aagatggctgAAGAACCTTTCAGACT	26-d1
	CmDMC1e	ACAACCTGCTCCTTGGTTTgg--gcaaagatggctgAAGAACCTTTCAGACT	22-d2
		ACAACCTGCTCCTTGGTTTggcagc---gatggctgAAGAACCTTTCAGACT	26-d3
	CmDMC1f	ACAACCTGCTCCTTGGTTT--cagcaaagatggctgAAGAACCTTTCAGACT	20-d2
		ACAACCTGCTCCTTGGTT--gcagcaaagatggctgAAGAACCTTTCAGACT	19-d2
SHc#15<br>*aabbCCddeeff*	*CmDMC1a*	ACAACCTGCTCCTTGGTTTggcag-aaagatggctgAAGAACCTTTCAGACT	25-d1
		ACAACCTGCTCCTTGGTTTggcagcaaagatggctgAAGGAACCTTTCAGACT	40-i1
	CmDMC1b	ACAACCTGCTCCTTGGTT--gcagcaaagatggctgAAGAACCTTTCAGACT	19-d2
		ACAACCTGCTCCTTGGTTT--cagcaaagatggctgAAGAACCTTTCAGACT	20-d2
	CmDMC1c	ACAACCTGCTCCTTGGTTTggcagcaaagatggctgAAGAACCTTTCAGACT	no mutation
		ACAACCTGCTCCTTGGTTTggcagcaaagatggctgAAGAACCTTTCAGACT	no mutation
	CmDMC1d	ACAACCTGCTCCTTGGTTTggcagc--agatggctgAAGAACCTTTCAGACT	26-d2
		ACAACCTGCTCCTTGGTTTggcagcaaagatggctgAAGGAACCTTTCAGACT	40-i1
	CmDMC1e	ACAACCTGCTCCTTGGTTTgg-agcaaagatggctgAAGAACCTTTCAGACT	22-d1
		ACAACCTGCTCCTTGGTTTg--agcaaagatggctgAAGAACCTTTCAGACT	21-d2
	CmDMC1f	ACAACCTGCTCCTTGGTTT--cagcaaagatggctgAAGAACCTTTCAGACT	20-d2
		ACAACCTGCTCCTTGGTTT--cagcaaagatggctgAAGAACCTTTCAGACT	20-d2
SHd#16<br>*aabbccDDeeff*	*CmDMC1a*	ACAACCTGCTCCTTGGTTTggca-caaagatggctgAAGAACCTTTCAGACT	24-d1
		ACAACCTGCTCCTTGGTTTggca-caaagatggctgAAGAACCTTTCAGACT	24-d1
	CmDMC1b	ACAACCTGCTCCTTGGTTTggcagc--agatggctgAAGAACCTTTCAGACT	26-d2
		ACAACCTGCTCCTTGGTTTggcagc--cgatggctgAAGAACCTTTCAGACT	26-d2, 28-s1
	CmDMC1c	ACAACCTGCTCCTTGGTTTggcagc--agatggctgAAGAACCTTTCAGACT	26-d2
		ACAACCTGCTCCTTGGTTTggcagc--agatggctgAAGAACCTTTCAGACT	26-d2
	CmDMC1d	ACAACCTGCTCCTTGGTTTggcagcaaagatggctgAAGAACCTTTCAGACT	no mutation
		ACAACCTGCTCCTTGGTTTggcagcaaagatggctgAAGAACCTTTCAGACT	no mutation
	CmDMC1e	ACAACCTGCTCCTTGGTTTgg-agcaaagatggctgAAGAACCTTTCAGACT	22-d1
		ACAACCTGCTCCTTGGTTTgg--gcaaagatggctgAAGAACCTTTCAGACT	22-d2
	CmDMC1f	ACAACCTGCTCCTTGGTTTgg--gcaaagatggctgAAGAACCTTTCAGACT	22-d2
		ACAACCTGCTCCTTGGTTTggc--caaagatggctgAAGAACCTTTCAGACT	23-d2

Supplementary Table S5 (Continued)

Line	Gene	Sequence	Mutation
SHe#19 *aabbccddEEff*	*CmDMC1a*	ACAACCTGCTCCTTGGTTTggcagca--gatggctgAAGAACCTTTCAGACT	27-d2
		ACAACCTGCTCCTTGGTTTggcagca--gatggctgAAGAACCTTTCAGACT	27-d2
	CmDMC1b	ACAACCTGCTCCTTGGTTTggcagc--cgatggctgAAGAACCTTTCAGACT	26-d2, 28-s1
		ACAACCTGCTCCTTGGTTTggcagc--cgatggctgAAGAACCTTTCAGACT	26-d2, 28-s1
	CmDMC1c	ACAACCTGCTCCTTGGTTTggcag-aaagatggctgAAGAACCTTTCAGACT	25-d1
		ACAACCTGCTCCTTGGTTTggca--aaagatggctgAAGAACCTTTCAGACT	24-d2
	CmDMC1d	ACAACCTGCTCCTTGGTTTggca-caaagatggctgAAGAACCTTTCAGACT	24-d1
		ACAACCTGCTCCTTGGTTTggca-caaagatggctgAAGAACCTTTCAGACT	24-d1
	CmDMC1e	ACAACCTGCTCCTTGGTTTggcagcaaagatggctgAAGAACCTTTCAGACT	no mutation
		ACAACCTGCTCCTTGGTTTggcagcaaagatggctgAAGAACCTTTCAGACT	no mutation
	CmDMC1f	ACAACCTGCTCCTTGGTTTgg-agcaaagatggctgAAGAACCTTTCAGACT	22-d1
		ACAACCTGCTCCTTGGTTTgg-agcaaagatggctgAAGAACCTTTCAGACT	22-d1
SHf#20 *aabbccddeeFF*	*CmDMC1a*	ACAACCTGCTCCTTGGTTTggcag-aaagatggctgAAGAACCTTTCAGACT	25-d1
		ACAACCTGCTCCTTGGTTTggcag-aaagatggctgAAGAACCTTTCAGACT	25-d1
	CmDMC1b	ACAACCTGCTCCTTGGTT-ggcagcaaagatggctgAAGAACCTTTCAGACT	19-d1
		ACAACCTGCTCCTTGGTT-ggcagcaaagatggctgAAGAACCTTTCAGACT	19-d1
	CmDMC1c	ACAACCTGCTCCTTGGTTTggcagc-aagatggctgAAGAACCTTTCAGACT	26-d1
		ACAACCTGCTCCTTGGTTTggcagcaaagatggctgAAGGAACCTTTCAGACT	40-i1
	CmDMC1d	ACAACCTGCTCCTTGGTTTggcagc--agatggctgAAGAACCTTTCAGACT	26-d2
		ACAACCTGCTCCTTGGTTTggcagc---gatggctgAAGAACCTTTCAGACT	26-d3
	CmDMC1e	ACAACCTGCTCCTTGGTTTggc-gcaaagatggctgAAGAACCTTTCAGACT	23-d1
		ACAACCTGCTCCTTGGTTTggc-gcaaagatggctgAAGAACCTTTCAGACT	23-d1
	CmDMC1f	ACAACCTGCTCCTTGGTTTggcagcaaagatggctgAAGAACCTTTCAGACT	no mutation
		ACAACCTGCTCCTTGGTTTggcagcaaagatggctgAAGAACCTTTCAGACT	no mutation
Yamate-shiro (Control: YS) *AABBCCDDEEFF*		ACAACCTGCTCCTTGGTTTggcagcaaagatggctgAAGAACCTTTCAGACT	
YS#16 *aabbccddeeff*	*CmDMC1a*	ACAACCTGCTCCTTGGTTTggca-caaagatggctgAAGAACCTTTCAGACT	24-d1
		ACAACCTGCTCCTTGGTTTggca-caaagatggctgAAGAACCTTTCAGACT	24-d1
	CmDMC1b	ACAACCTGCTCCTTGGTT--gcagcaaagatggctgAAGAACCTTTCAGACT	19-d2
		ACAACCTGCTCCTTGGTT--gcagcaaagatggctgAAGAACCTTTCAGACT	19-d2
	CmDMC1c	ACAACCTGCTCCTTGGTTTgg--gcaaagatggctgAAGAACCTTTCAGACT	22-d2
		ACAACCTGCTCCTTGGTTTgg--gcaaagatggctgAAGAACCTTTCAGACT	22-d2
	CmDMC1d	ACAACCTGCTCCTTGGTTTggcag--aagatggctgAAGAACCTTTCAGACT	25-d2
		ACAACCTGCTCCTTGGTTTggcag--aagatggctgAAGAACCTTTCAGACT	25-d2
	CmDMC1e	ACAACCTGCTCCTTGGTTTgg-agcaaagatggctgAAGAACCTTTCAGACT	22-d1
		ACAACCTGCTCCTTGGTTTgg--gcaaagatggctgAAGAACCTTTCAGACT	22-d2
	CmDMC1f	ACAACCTGCTCCTTGGTTTgg--gcaaagatggctgAAGAACCTTTCAGACT	22-d2
		ACAACCTGCTCCTTGGTTTgg--gcaaagatggctgAAGAACCTTTCAGACT	22-d2
YSa#12 *AAbbccddeeff*	*CmDMC1a*	ACAACCTGCTCCTTGGTTTggcagcaaagatggctgAAGAACCTTTCAGACT	no mutation
		ACAACCTGCTCCTTGGTTTggcagcaaagatggctgAAGAACCTTTCAGACT	no mutation
	CmDMC1b	ACAACCTGCTCCTTG--CTggcagcaaagatggctgAAGAACCTTTCAGACT	16-d2, 18-s1
		ACAACCTGCTCCTTG--CTggcagcaaagatggctgAAGAACCTTTCAGACT	16-d2, 18-s1
	CmDMC1c	ACAACCTGCTCCTTGGTTTgg--gcaaagatggctgAAGAACCTTTCAGACT	22-d2
		ACAACCTGCTCCTTGGTTTgg--gcaaagatggctgAAGAACCTTTCAGACT	22-d2
	CmDMC1d	ACAACCTGCTCCTTGGTTTggcag--gagatggctgAAGAACCTTTCAGACT	25-d2, 27-s1
		ACAACCTGCTCCTTGGTTTggca----agatggctgAAGAACCTTTCAGACT	24-d4
	CmDMC1e	ACAACCTGCTCCTTGGTTTgg-agcaaagatggctgAAGAACCTTTCAGACT	22-d1
		ACAACCTGCTCCTTGGTTTgg-agcaaagatggctgAAGAACCTTTCAGACT	22-d1
	CmDMC1f	ACAACCTGCTCCTTGGTTTggc-gcaaagatggctgAAGAACCTTTCAGACT	23-d1
		ACAACCTGCTCCTTGGTTTggc--caaagatggctgAAGAACCTTTCAGACT	23-d2
YSb#13 *aaBBccddeeff*	*CmDMC1a*	ACAACCTGCTCCTTGGTTTggcagca-agatggctgAAGAACCTTTCAGACT	27-d1
		ACAACCTGCTCCTTGGTTTggcagca--gatggctgAAGAACCTTTCAGACT	27-d2
	CmDMC1b	ACAACCTGCTCCTTGGTTTggcagcaaagatggctgAAGAACCTTTCAGACT	no mutation
		ACAACCTGCTCCTTGGTTTggcagcaaagatggctgAAGAACCTTTCAGACT	no mutation
	CmDMC1c	ACAACCTGCTCCTTGGTTTgg-agcaaagatggctgAAGAACCTTTCAGACT	22-d1
		ACAACCTGCTCCTTGGTTTgg-agcaaagatggctgAAGAACCTTTCAGACT	22-d1
	CmDMC1d	ACAACCTGCTCCTTGGTTTggc-gcaaagatggctgAAGAACCTTTCAGACT	23-d1
		ACAACCTGCTCCTTGGTTTggc-gcaaagatggctgAAGAACCTTTCAGACT	23-d1
	CmDMC1e	ACAACCTGCTCCTTGGTTTg-cagcaaagatggctgAAGAACCTTTCAGACT	21-d1
		ACAACCTGCTCCTTGGTTTg--agcaaagatggctgAAGAACCTTTCAGACT	21-d2
	CmDMC1f	ACAACCTGCTCCTTGGTTTgg--gcaaagatggctgAAGAACCTTTCAGACT	22-d2
		ACAACCTGCTCCTTGGTTTgg--gcaaagatggctgAAGAACCTTTCAGACT	22-d2

Supplementary Table S5 (Continued)

YSc#27 *aabbCCddeeff*	*CmDMC1a*	ACAACCTGCTCCTTGGTTTggc-gcaaagatggctgAAGAACCTTTCAGACT	23-d1
		ACAACCTGCTCCTTGGTTTggc-gcaaagatggctgAAGAACCTTTCAGACT	23-d1
	CmDMC1b	ACAACCTGCTCCTTGGTTTgg-agcaaagatggctgAAGAACCTTTCAGACT	22-d1
		ACAACCTGCTCCTTGGTTTgg--gcaaagatggctgAAGAACCTTTCAGACT	22-d2
	CmDMC1c	ACAACCTGCTCCTTGGTTTggcagcaaagatggctgAAGAACCTTTCAGACT	no mutation
		ACAACCTGCTCCTTGGTTTggcagcaaagatggctgAAGAACCTTTCAGACT	no mutation
	CmDMC1d	ACAACCTGCTCCTTGGTTTggc-gcaaagatggctgAAGAACCTTTCAGACT	23-d1
		ACAACCTGCTCCTTGGTTTggcagcaaagatggctgAAG**G**AACCTTTCAGACT	40-i1
	CmDMC1e	ACAACCTGCTCCTTGGTTTgg--gcaaagatggctgAAGAACCTTTCAGACT	22-d2
		ACAACCTGCTCCTTGGTTTggcagc---gatggctgAAGAACCTTTCAGACT	26-d3
	CmDMC1f	ACAACCTGCTCCTTGGTTTggc-gcaaagatggctgAAGAACCTTTCAGACT	23-d1
		ACAACCTGCTCCTTGGTTTgg--gcaaagatggctgAAGAACCTTTCAGACT	22-d2
YSd#28 *aabbccDDeeff*	*CmDMC1a*	ACAACCTGCTCCTTGGTTTgg-agcaaagatggctgAAGAACCTTTCAGACT	22-d1
		ACAACCTGCTCCTTGGTTTgg--gcaaagatggctgAAGAACCTTTCAGACT	22-d2
	CmDMC1b	ACAACCTGCTCCTTGGTTTggc--caaagatggctgAAGAACCTTTCAGACT	23-d2
		ACAACCTGCTCCTTGGTTTgg--**a**caaagatggctgAAGAACCTTTCAGACT	22-d2, 24-s1
	CmDMC1c	ACAACCTGCTCCTTGGTTTgg--gcaaagatggctgAAGAACCTTTCAGACT	22-d2
		ACAACCTGCTCCTTGGTTTgg--gcaaagatggctgAAGAACCTTTCAGACT	22-d2
	CmDMC1d	ACAACCTGCTCCTTGGTTTggcagcaaagatggctgAAGAACCTTTCAGACT	no mutation
		ACAACCTGCTCCTTGGTTTggcagcaaagatggctgAAGAACCTTTCAGACT	no mutation
	CmDMC1e	ACAACCTGCTCCTTGGTTTggca-caaagatggctgAAGAACCTTTCAGACT	24-d1
		ACAACCTGCTCCTTGGTTTggca--aaagatggctgAAGAACCTTTCAGACT	24-d2
	CmDMC1f	ACAACCTGCTCCTTGGTTTgg-agcaaagatggctgAAGAACCTTTCAGACT	22-d1
		ACAACCTGCTCCTTGGTTTgg-agcaaagatggctgAAGAACCTTTCAGACT	22-d1
YSe#30 *aabbccddEEff*	*CmDMC1a*	ACAACCTGCTCCTTGGTTTggcagca-agatggctgAAGAACCTTTCAGACT	27-d1
		ACAACCTGCTCCTTGGTTTggcagcaaagatggctgAAG**G**AACCTTTCAGACT	40-i1
	CmDMC1b	ACAACCTGCTCCTTGGTT--gcagcaaagatggctgAAGAACCTTTCAGACT	19-d2
		ACAACCTGCTCCTTGGTT--gcagcaaagatggctgAAGAACCTTTCAGACT	19-d2
	CmDMC1c	ACAACCTGCTCCTTGGTTTgg-agcaaagatggctgAAGAACCTTTCAGACT	22-d1
		ACAACCTGCTCCTTGGTTTgg--gcaaagatggctgAAGAACCTTTCAGACT	22-d2
	CmDMC1d	ACAACCTGCTCCTTGGTTTggc--caaagatggctgAAGAACCTTTCAGACT	23-d2
		ACAACCTGCTCCTTGGTTTggc--caaagatggctgAAGAACCTTTCAGACT	23-d2
	CmDMC1e	ACAACCTGCTCCTTGGTTTggcagcaaagatggctgAAGAACCTTTCAGACT	no mutation
		ACAACCTGCTCCTTGGTTTggcagcaaagatggctgAAGAACCTTTCAGACT	no mutation
	CmDMC1f	ACAACCTGCTCCTTGGTTTggca-caaagatggctgAAGAACCTTTCAGACT	24-d1
		ACAACCTGCTCCTTGGTTTggca-caaagatggctgAAGAACCTTTCAGACT	24-d1
YSf#32 *aabbccddeeFF*	*CmDMC1a*	ACAACCTGCTCCTTGGTTTgg-agcaaagatggctgAAGAACCTTTCAGACT	22-d1
		ACAACCTGCTCCTTGGTTTgg-agcaaagatggctgAAGAACCTTTCAGACT	22-d1
	CmDMC1b	ACAACCTGCTCCTTGGTT-ggcagcaaagatggctgAAGAACCTTTCAGACT	19-d1
		ACAACCTGCTCCTTGGTT--gcagcaaagatggctgAAGAACCTTTCAGACT	19-d2
	CmDMC1c	ACAACCTGCTCCTTGGTTTggc-gcaaagatggctgAAGAACCTTTCAGACT	23-d1
		ACAACCTGCTCCTTGGTTTggc--caaagatggctgAAGAACCTTTCAGACT	23-d2
	CmDMC1d	ACAACCTGCTCCTTGGTTTgg--gcaaagatggctgAAGAACCTTTCAGACT	22-d2
		ACAACCTGCTCCTTGGTTTgg----aaagatggctgAAGAACCTTTCAGACT	22-d4
	CmDMC1e	ACAACCTGCTCCTTGGTTTggca-caaagatggctgAAGAACCTTTCAGACT	24-d1
		ACAACCTGCTCCTTGGTTTggca-caaagatggctgAAGAACCTTTCAGACT	24-d1
	CmDMC1f	ACAACCTGCTCCTTGGTTTggcagcaaagatggctgAAGAACCTTTCAGACT	no mutation
		ACAACCTGCTCCTTGGTTTggcagcaaagatggctgAAGAACCTTTCAGACT	no mutation

Each spacer region between the two TALEN binding sites is shown with lowercase letters.
Red-color hyphens represent deleted nucleotides. Blue-color and green-color bold letters represent inserted nucleotides and substituted nucleotides, respectively. For each *CmDMC1* locus (two alleles), mutation types shown with orange-color background indicate biallelic and different mutation patterns in the corresponding alleles.
d#, number of nucleotide(s) deleted; i1, a nucleotide insertion; s1, a nucleotide substitution.

Supplementary Table S6. Mutation patterns of *CmDMC1* including TALENs target site.

Line	Gene	Amino acid sequences including TALENs target site
Shuho-no-chikara (Control: SH)	*CmDMC1a*	201>TYEHQYNLLLGLAAKMAEEPFRLLIVDSVIALFRVDFTGR>240
SH#12	*CmDMC1a-1*	201>TYEHQYNLLLGLAERWLKNLSDY*>223
	CmDMC1a-2	201>TYEHQYNLLLGLAERWLKNLSDY*>223
SHa#13	*CmDMC1a-1*	201>TYEHQYNLLLGLAAKMAEEPFRLLIVDSVIALFRVDFTGR>240
	CmDMC1a-2	201>TYEHQYNLLLGLAAKMAEEPFRLLIVDSVIALFRVDFTGR>240
SHb#14	*CmDMC1a-1*	201>TYEHQYNLLLGLAEDG*>216
	CmDMC1a-2	201>TYEHQYNLLLGLAEDG*>216
SHc#15	*CmDMC1a-1*	201>TYEHQYNLLLGLAERWLKNLSDY*>223
	CmDMC1a-2	201>TYEHQYNLLLGLAAKMAEGTFQTTDC*>226
SHd#16	*CmDMC1a-1*	201>TYEHQYNLLLGLAQRWLKNLSDY*>223
	CmDMC1a-2	201>TYEHQYNLLLGLAQRWLKNLSDY*>223
SHe#19	*CmDMC1a-1*	201>TYEHQYNLLLGLAADG*>216
	CmDMC1a-2	201>TYEHQYNLLLGLAADG*>216
SHf#20	*CmDMC1a-1*	201>TYEHQYNLLLGLAERWLKNLSDY*>223
	CmDMC1a-2	201>TYEHQYNLLLGLAERWLKNLSDY*>223
Shuho-no-chikara (Control: SH)	*CmDMC1b*	201>TYEHQYNLLLGLAAKMAEEPFRLLIVDSVIALFRVDFTGR>240
SH#12	*CmDMC1b-1*	201>TYEHQYNLLLGWQQRWLKNLSDY*>223
	CmDMC1b-2	201>TYEHQYNLLLGWQQRWLKNLSDY*>223
SHa#13	*CmDMC1b-1*	201>TYEHQYNLLLGLAADG*>216
	CmDMC1b-2	201>TYEHQYNLLLGLAADG*>216
SHb#14	*CmDMC1b-1*	201>TYEHQYNLLLGLAAKMAEEPFRLLIVDSVIALFRVDFTGR>240
	CmDMC1b-2	201>TYEHQYNLLLGLAAKMAEEPFRLLIVDSVIALFRVDFTGR>240
SHc#15	*CmDMC1b-1*	201>TYEHQYNLLLGCSKDG*>216
	CmDMC1b-2	201>TYEHQYNLLLGFSKDG*>216
SHd#16	*CmDMC1b-1*	201>TYEHQYNLLLGLAADG*>216
	CmDMC1b-2	201>TYEHQYNLLLGLAADG*>216
SHe#19	*CmDMC1b-1*	201>TYEHQYNLLLGLAADG*>216
	CmDMC1b-2	201>TYEHQYNLLLGLAADG*>216
SHf#20	*CmDMC1b-1*	201>TYEHQYNLLLGWQQRWLKNLSDY*>223
	CmDMC1b-2	201>TYEHQYNLLLGWQQRWLKNLSDY*>223
Shuho-no-chikara (Control: SH)	*CmDMC1c*	201>TYEHQYNLLLGLAAKMAEEPFRLLIVDSVIALFRVDFTGR>240
SH#12	*CmDMC1c-1*	201>TYEHQYNLLLGLAEDG*>216
	CmDMC1c-2	201>TYEHQYNLLLGLAAKMAEGTFQTTDC*>226
SHa#13	*CmDMC1c-1*	201>TYEHQYNLLLGLAEDG*>216
	CmDMC1c-2	201>TYEHQYNLLLGLAVDG*>216
SHb#14	*CmDMC1c-1*	201>TYEHQYNLLLGLAERWLKNLSDY*>223
	CmDMC1c-2	201>TYEHQYNLLLGLAEDG*>216
SHc#15	*CmDMC1c-1*	201>TYEHQYNLLLGLAAKMAEEPFRLLIVDSVIALFRVDFTGR>240
	CmDMC1c-2	201>TYEHQYNLLLGLAAKMAEEPFRLLIVDSVIALFRVDFTGR>240
SHd#16	*CmDMC1c-1*	201>TYEHQYNLLLGLAADG*>216
	CmDMC1c-2	201>TYEHQYNLLLGLAADG*>216
SHe#19	*CmDMC1c-1*	201>TYEHQYNLLLGLAERWLKNLSDY*>223
	CmDMC1c-2	201>TYEHQYNLLLGLAKDG*>216
SHf#20	*CmDMC1c-1*	201>TYEHQYNLLLGLAARWLKNLSDY*>223
	CmDMC1c-2	201>TYEHQYNLLLGLAAKMAEGTFQTTDC*>226
Shuho-no-chikara (Control: SH)	*CmDMC1d*	201>TYEHQYNLLLGLAAKMAEEPFRLLIVDSVIALFRVDFTGR>240
SH#12	*CmDMC1d-1*	201>TYEHQYNLLLGLAARWLKNLSDY*>223
	CmDMC1d-2	201>TYEHQYNLLLGLAAKMAEGTFQTTDC*>226
SHa#13	*CmDMC1d-1*	201>TYEHQYNLLLGLAA-MAEEPFRLLIVDSVIALFRVDFTGR>239
	CmDMC1d-2	201>TYEHQYNLLLGLAARWLKNLSDY*>223
SHb#14	*CmDMC1d-1*	201>TYEHQYNLLLGLAARWLKNLSDY*>223
	CmDMC1d-2	201>TYEHQYNLLLGLAARWLKNLSDY*>223
SHc#15	*CmDMC1d-1*	201>TYEHQYNLLLGLAADG*>216
	CmDMC1d-2	201>TYEHQYNLLLGLAAKMAEGTFQTTDG*>226
SHd#16	*CmDMC1d-1*	201>TYEHQYNLLLGLAAKMAEEPFRLLIVDSVIALFRVDFTGR>240
	CmDMC1d-2	201>TYEHQYNLLLGLAAKMAEEPFRLLIVDSVIALFRVDFTGR>240
SHe#19	*CmDMC1d-1*	201>TYEHQYNLLLGLAQRWLKNLSDY*>223
	CmDMC1d-2	201>TYEHQYNLLLGLAQRWLKNLSDY*>223
SHf#20	*CmDMC1d-1*	201>TYEHQYNLLLGLAADG*>216
	CmDMC1d-2	201>TYEHQYNLLLGLAA-MAEEPFRLLIVDSVIALFRVDFTGR>239
Shuho-no-chikara (Control: SH)	*CmDMC1e*	201>TYEHQYNLLLGLAAKMAEEPFRLLIVDSVIALFRVDFTGR>240
SH#12	*CmDMC1e-1*	201>TYEHQYNLLLGLEQRWLKNLSDY*>223
	CmDMC1e-2	201>TYEHQYNLLLGLEQRWLKNLSDY*>223
SHa#13	*CmDMC1e-1*	201>TYEHQYNLLLGLAKDG*>216
	CmDMC1e-2	201>TYEHQYNLLLGLAKDG*>216
SHb#14	*CmDMC1e-1*	201>TYEHQYNLLLGLGKDG*>216
	CmDMC1e-2	201>TYEHQYNLLLGLAA-MAEEPFRLLIVDSVIALFRVDFTGR>239
SHc#15	*CmDMC1e-1*	201>TYEHQYNLLLGLEQRWLKNLSDY*>223
	CmDMC1e-2	201>TYEHQYNLLLGLSKDG*>216
SHd#16	*CmDMC1e-1*	201>TYEHQYNLLLGLEQRWLKNLSDY*>223
	CmDMC1e-2	201>TYEHQYNLLLGLGKDG*>216
SHe#19	*CmDMC1e-1*	201>TYEHQYNLLLGLAAKMAEEPFRLLIVDSVIALFRVDFTGR>240
	CmDMC1e-2	201>TYEHQYNLLLGLAAKMAEEPFRLLIVDSVIALFRVDFTGR>240
SHf#20	*CmDMC1e-1*	201>TYEHQYNLLLGLAQRWLKNLSDY*>223
	CmDMC1e-2	201>TYEHQYNLLLGLAQRWLKNLSDY*>223
Shuho-no-chikara (Control: SH)	*CmDMC1f*	201>TYEHQYNLLLGLAAKMAEEPFRLLIVDSVIALFRVDFTGR>240
SH#12	*CmDMC1f-1*	201>TYEHQYNLLLGLQQRWLKNLSDY*>223
	CmDMC1f-2	201>TYEHQYNLLLGLQQRWLKNLSDY*>223
SHa#13	*CmDMC1f-1*	201>TYEHQYNLLLGLQQRWLKNLSDY*>223
	CmDMC1f-2	201>TYEHQYNLLLGLAAKMAEGTFQTTDC*>226
SHb#14	*CmDMC1f-1*	201>TYEHQYNLLLGFSKDG*>216
	CmDMC1f-2	201>TYEHQYNLLLGCSKDG*>216
SHc#15	*CmDMC1f-1*	201>TYEHQYNLLLGFSKDG*>216
	CmDMC1f-2	201>TYEHQYNLLLGFSKDG*>216
SHd#16	*CmDMC1f-1*	201>TYEHQYNLLLGLGKDG*>216
	CmDMC1f-2	201>TYEHQYNLLLGLAKDG*>216
SHe#19	*CmDMC1f-1*	201>TYEHQYNLLLGLEQRWLKNLSDY*>223
	CmDMC1f-2	201>TYEHQYNLLLGLEQRWLKNLSDY*>223
SHf#20	*CmDMC1f-1*	201>TYEHQYNLLLGLAAKMAEEPFRLLIVDSVIALFRVDFTGR>240
	CmDMC1f-2	201>TYEHQYNLLLGLAAKMAEEPFRLLIVDSVIALFRVDFTGR>240

Supplementary Table S6 (continued)

Yamate-shiro (Control: YS)	*CmDMC1a*	201>TYEHQYNLLLGLAAKMAEEPFRLLIVDSVIALFRVDFTGR>240
YS#16	*CmDMC1a-1*	201>TYEHQYNLLLGLAQRWLKNLSDY*>223
	CmDMC1a-2	201>TYEHQYNLLLGLAQRWLKNLSDY*>223
YSa#12	*CmDMC1a-1*	201>TYEHQYNLLLGLAAKMAEEPFRLLIVDSVIALFRVDFTGR>240
	CmDMC1a-2	201>TYEHQYNLLLGLAAKMAEEPFRLLIVDSVIALFRVDFTGR>240
YSb#13	*CmDMC1a-1*	201>TYEHQYNLLLGLAARWLKNLSDY*>223
	CmDMC1a-2	201>TYEHQYNLLLGLAADG*>216
YSc#27	*CmDMC1a-1*	201>TYEHQYNLLLGLAQRWLKNLSDY*>223
	CmDMC1a-2	201>TYEHQYNLLLGLAQRWLKNLSDY*>223
YSd#28	*CmDMC1a-1*	201>TYEHQYNLLLGLEQRWLKNLSDY*>223
	CmDMC1a-2	201>TYEHQYNLLLGLGKDG*>216
YSe#30	*CmDMC1a-1*	201>TYEHQYNLLLGLAARWLKNLSDY*>223
	CmDMC1a-2	201>TYEHQYNLLLGLAAKMAEGTFQTTDC*>226
YSf#32	*CmDMC1a-1*	201>TYEHQYNLLLGLEQRWLKNLSDY*>223
	CmDMC1a-2	201>TYEHQYNLLLGLEQRWLKNLSDY*>223
Yamate-shiro (Control: YS)	*CmDMC1b*	201>TYEHQYNLLLGLAAKMAEEPFRLLIVDSVIALFRVDFTGR>240
YS#16	*CmDMC1b-1*	201>TYEHQYNLLLGCSKDG*>216
	CmDMC1b-2	201>TYEHQYNLLLGCSKDG*>216
YSa#12	*CmDMC1b-1*	201>TYEHQYNLLLAGSKDG*>216
	CmDMC1b-2	201>TYEHQYNLLLAGSKDG*>216
YSb#13	*CmDMC1b-1*	201>TYEHQYNLLLGLAAKMAEEPFRLLIVDSVIALFRVDFTGR>240
	CmDMC1b-2	201>TYEHQYNLLLGLAAKMAEEPFRLLIVDSVIALFRVDFTGR>240
YSc#27	*CmDMC1b-1*	201>TYEHQYNLLLGLEQRWLKNLSDY*>223
	CmDMC1b-2	201>TYEHQYNLLLGLGKDG*>216
YSd#28	*CmDMC1b-1*	201>TYEHQYNLLLGLAKDG*>216
	CmDMC1b-2	201>TYEHQYNLLLGLDKDG*>216
YSe#30	*CmDMC1b-1*	201>TYEHQYNLLLGCSKDG*>216
	CmDMC1b-2	201>TYEHQYNLLLGCSKDG*>216
YSf#32	*CmDMC1b-1*	201>TYEHQYNLLLGWQQRWLKNLSDY*>223
	CmDMC1b-2	201>TYEHQYNLLLGCSKDG*>216
Yamate-shiro (Control: YS)	*CmDMC1c*	201>TYEHQYNLLLGLAAKMAEEPFRLLIVDSVIALFRVDFTGR>240
YS#16	*CmDMC1c-1*	201>TYEHQYNLLLGLGKDG*>216
	CmDMC1c-2	201>TYEHQYNLLLGLGKDG*>216
YSa#12	*CmDMC1c-1*	201>TYEHQYNLLLGLGKDG*>216
	CmDMC1c-2	201>TYEHQYNLLLGLGKDG*>216
YSb#13	*CmDMC1c-1*	201>TYEHQYNLLLGLEQRWLKNLSDY*>223
	CmDMC1c-2	201>TYEHQYNLLLGLEQRWLKNLSDY*>223
YSc#27	*CmDMC1c-1*	201>TYEHQYNLLLGLAAKMAEEPFRLLIVDSVIALFRVDFTGR>240
	CmDMC1c-2	201>TYEHQYNLLLGLAAKMAEEPFRLLIVDSVIALFRVDFTGR>240
YSd#28	*CmDMC1c-1*	201>TYEHQYNLLLGLGKDG*>216
	CmDMC1c-2	201>TYEHQYNLLLGLGKDG*>216
YSe#30	*CmDMC1c-1*	201>TYEHQYNLLLGLEQRWLKNLSDY*>223
	CmDMC1c-2	201>TYEHQYNLLLGLGKDG*>216
YSf#32	*CmDMC1c-1*	201>TYEHQYNLLLGLAQRWLKNLSDY*>223
	CmDMC1c-2	201>TYEHQYNLLLGLAKDG*>216
Yamate-shiro (Control: YS)	*CmDMC1d*	201>TYEHQYNLLLGLAAKMAEEPFRLLIVDSVIALFRVDFTGR>240
YS#16	*CmDMC1d-1*	201>TYEHQYNLLLGLAEDG*>216
	CmDMC1d-2	201>TYEHQYNLLLGLAEDG*>216
YSa#12	*CmDMC1d-1*	201>TYEHQYNLLLGLAGDG*>216
	CmDMC1d-2	201>TYEHQYNLLLGLARWLKNLSDY*>222
YSb#13	*CmDMC1d-1*	201>TYEHQYNLLLGLAQRWLKNLSDY*>223
	CmDMC1d-2	201>TYEHQYNLLLGLAQRWLKNLSDY*>223
YSc#27	*CmDMC1d-1*	201>TYEHQYNLLLGLAQRWLKNLSDY*>223
	CmDMC1d-2	201>TYEHQYNLLLGLAAKMAEGTFQTTDC*>226
YSd#28	*CmDMC1d-1*	201>TYEHQYNLLLGLAAKMAEEPFRLLIVDSVIALFRVDFTGR>240
	CmDMC1d-2	201>TYEHQYNLLLGLAAKMAEEPFRLLIVDSVIALFRVDFTGR>240
YSe#30	*CmDMC1d-1*	201>TYEHQYNLLLGLAKDG*>216
	CmDMC1d-2	201>TYEHQYNLLLGLAKDG*>216
YSf#32	*CmDMC1d-1*	201>TYEHQYNLLLGLGKDG*>216
	CmDMC1d-2	201>TYEHQYNLLLGLERWLKNLSDY*>222
Yamate-shiro (Control: YS)	*CmDMC1e*	201>TYEHQYNLLLGLAAKMAEEPFRLLIVDSVIALFRVDFTGR>240
YS#16	*CmDMC1e-1*	201>TYEHQYNLLLGLEQRWLKNLSDY*>223
	CmDMC1e-2	201>TYEHQYNLLLGLGKDG*>216
YSa#12	*CmDMC1e-1*	201>TYEHQYNLLLGLEQRWLKNLSDY*>223
	CmDMC1e-2	201>TYEHQYNLLLGLEQRWLKNLSDY*>223
YSb#13	*CmDMC1e-1*	201>TYEHQYNLLLGLQQRWLKNLSDY*>223
	CmDMC1e-2	201>TYEHQYNLLLGLSKDG*>216
YSc#27	*CmDMC1e-1*	201>TYEHQYNLLLGLGKDG*>216
	CmDMC1e-2	201>TYEHQYNLLLGLAA-MAEEPFRLLIVDSVIALFRVDFTGR>239
YSd#28	*CmDMC1e-1*	201>TYEHQYNLLLGLAQRWLKNLSDY*>223
	CmDMC1e-2	201>TYEHQYNLLLGLAKDG*>216
YSe#30	*CmDMC1e-1*	201>TYEHQYNLLLGLAAKMAEEPFRLLIVDSVIALFRVDFTGR>240
	CmDMC1e-2	201>TYEHQYNLLLGLAAKMAEEPFRLLIVDSVIALFRVDFTGR>240
YSf#32	*CmDMC1e-1*	201>TYEHQYNLLLGLAQRWLKNLSDY*>223
	CmDMC1e-2	201>TYEHQYNLLLGLAQRWLKNLSDY*>223
Yamate-shiro (Control: YS)	*CmDMC1f*	201>TYEHQYNLLLGLAAKMAEEPFRLLIVDSVIALFRVDFTGR>240
YS#16	*CmDMC1f-1*	201>TYEHQYNLLLGLGKDG*>216
	CmDMC1f-2	201>TYEHQYNLLLGLGKDG*>216
YSa#12	*CmDMC1f-1*	201>TYEHQYNLLLGLAQRWLKNLSDY*>223
	CmDMC1f-2	201>TYEHQYNLLLGLAKDG*>216
YSb#13	*CmDMC1f-1*	201>TYEHQYNLLLGLGKDG*>216
	CmDMC1f-2	201>TYEHQYNLLLGLGKDG*>216
YSc#27	*CmDMC1f-1*	201>TYEHQYNLLLGLAQRWLKNLSDY*>223
	CmDMC1f-2	201>TYEHQYNLLLGLGKDG*>216
YSd#28	*CmDMC1f-1*	201>TYEHQYNLLLGLEQRWLKNLSDY*>223
	CmDMC1f-2	201>TYEHQYNLLLGLEQRWLKNLSDY*>223
YSe#30	*CmDMC1f-1*	201>TYEHQYNLLLGLAQRWLKNLSDY*>223
	CmDMC1f-2	201>TYEHQYNLLLGLAQRWLKNLSDY*>223
YSf#32	*CmDMC1f-1*	201>TYEHQYNLLLGLAAKMAEEPFRLLIVDSVIALFRVDFTGR>240
	CmDMC1f-2	201>TYEHQYNLLLGLAAKMAEEPFRLLIVDSVIALFRVDFTGR>240

Orange-color letters indicate Walker-B motifs and red-color letters indicate mutated amino acid sequences compared to non-mutated *CmDMC1* gene.

* indicates stop codon.

Supplementary Table S7. Growth characteristics of *CmDMC1* -edited and non-transgenic chrysanthemums

Line	Stem length (cm)	Number of leaves[1)]	Leaf size[2)] (cm)		Flower color	Number of head flowers	Diameter of head flower (cm)
			length	width			
Shuho-no-chikara	32.5 ± 0.5	13.5 ± 0.2	3.5 ± 0.1	2.5 ± 0.2	white	3.0 ± 0.0	6.5 ± 0.2
SH#12	33.5 ± 0.2	13.5 ± 0.1	3.4 ± 0.0	2.4 ± 0.1	white	2.8 ± 0.1	6.6 ± 0.1
SHa#13	31.3 ± 0.1	14.0 ± 0.1	3.5 ± 0.0	2.3 ± 0.1	white	3.0 ± 0.1	6.6 ± 0.1
SHb#14	33.5 ± 0.2	12.5 ± 0.0	3.4 ± 0.0	2.4 ± 0.1	white	2.8 ± 0.1	6.5 ± 0.1
SHc#15	32.4 ± 0.3	13.0 ± 0.1	3.5 ± 0.0	2.4 ± 0.1	white	3.2 ± 0.1	6.6 ± 0.1
SHd#16	31.4 ± 0.1	13.5 ± 0.0	3.4 ± 0.0	2.4 ± 0.1	white	3.0 ± 0.1	6.4 ± 0.1
SHe#19	32.2 ± 0.2	14.0 ± 0.1	3.4 ± 0.0	2.3 ± 0.1	white	2.8 ± 0.1	6.5 ± 0.1
SHf#20	34.5 ± 0.2	14.0 ± 0.0	3.5 ± 0.0	2.4 ± 0.1	white	3.0 ± 0.1	6.6 ± 0.1
Yamate-shiro (Control: YS)	35.5 ± 0.6	25.0 ± 0.1	4.0 ± 0.1	3.0 ± 0.1	white	6.5 ± 0.1	3.2 ± 0.1
YS#16	36.5 ± 0.3	26.0 ± 0.1	4.2 ± 0.2	3.1 ± 0.1	white	6.4 ± 0.0	3.4 ± 0.1
YSa#12	34.5 ± 0.2	25.0 ± 0.1	4.0 ± 0.1	3.0 ± 0.1	white	7.0 ± 0.0	3.2 ± 0.1
YSb#13	35.0 ± 0.2	25.0 ± 0.1	4.1 ± 0.0	3.1 ± 0.0	white	7.0 ± 0.0	3.2 ± 0.0
YSc#27	35.0 ± 0.3	26.0 ± 0.1	4.0 ± 0.2	3.0 ± 0.0	white	6.5 ± 0.0	3.2 ± 0.1
YSd#28	35.5 ± 0.2	27.0 ± 0.1	4.0 ± 0.0	3.1 ± 0.1	white	6.4 ± 0.1	3.4 ± 0.0
YSe#30	36.5 ± 0.3	26.0 ± 0.1	4.1 ± 0.2	3.0 ± 0.0	white	7.0 ± 0.0	3.4 ± 0.1
YSf#32	35.0 ± 0.1	25.0 ± 0.1	4.2 ± 0.2	3.0 ± 0.1	white	7.0 ± 0.1	3.3 ± 0.1

Ten plants per line were used for the observation and measurements.

Each value represents the mean ± SE.

1) Includes the leaves which withered and died.

2) Measured the leaves on the intermediate part of each stem.

Supplementary Table S8. Evaluation of male sterility in *CmDMC1* -edited plants and non-transgenic controls by Alexander staining.

Line	No. of mutated alleles	Name of non-mutated gene(s)	10 °C			15 °C			20 °C			25 °C			30 °C		
			No. of VP (A)	No. of AP (B)	Ratio of VP (C in %)	No. of VP (A)	No. of AP (B)	Ratio of VP (C in %)	No. of VP (A)	No. of AP (B)	Ratio of VP (C in %)	No. of VP (A)	No. of AP (B)	Ratio of VP (C in %)	No. of VP (A)	No. of AP (B)	Ratio of VP (C in %)
Shuho-no-chikara	0	all	19.5 ± 1.6	49.5 ± 2.0	28.3 ± 1.7	154.2 ± 1.2	145.7 ± 4.2	51.4 ± 0.7	389.6 ± 2.2	89.6 ± 2.2	81.3 ± 1.8	191.5 ± 1.3	112.4 ± 2.2	63.0 ± 0.5	0.0 ± 0.0	0.0 ± 0.0	-
SH#12	12	none	0.0 ± 0.0	0.0 ± 0.0	-	0.0 ± 0.0	0.0 ± 0.0	-	0.0 ± 0.0	0.0 ± 0.0	-	0.0 ± 0.0	0.0 ± 0.0	-	0.0 ± 0.0	0.0 ± 0.0	-
SHa#13	10	*CmDMC1a*	0.0 ± 0.0	0.0 ± 0.0	-	11.2 ± 1.4	22.3 ± 2.2	33.4 ± 3.5 **	24.2 ± 1.6	36.3 ± 1.6	40.0 ± 1.3 **	9.2 ± 1.4	24.7 ± 2.2	27.1 ± 2.4 **	0.0 ± 0.0	0.0 ± 0.0	-
SHb#14	10	*CmDMC1b*	0.0 ± 0.0	0.0 ± 0.0	-	13.8 ± 1.5	25.5 ± 1.9	35.0 ± 2.4 **	21.8 ± 1.4	34.0 ± 2.0	39.1 ± 1.1 **	9.8 ± 1.2	28.9 ± 2.6	25.2 ± 1.4 **	0.0 ± 0.0	0.0 ± 0.0	-
SHc#15	10	*CmDMC1c*	0.0 ± 0.0	0.0 ± 0.0	-	0.0 ± 0.0	0.0 ± 0.0	-	0.0 ± 0.0	0.0 ± 0.0	-	0.0 ± 0.0	0.0 ± 0.0	-	0.0 ± 0.0	0.0 ± 0.0	-
SHd#16	10	*CmDMC1d*	0.0 ± 0.0	0.0 ± 0.0	-	0.0 ± 0.0	0.0 ± 0.0	-	0.0 ± 0.0	0.0 ± 0.0	-	0.0 ± 0.0	0.0 ± 0.0	-	0.0 ± 0.0	0.0 ± 0.0	-
SHe#19	10	*CmDMC1e*	0.0 ± 0.0	0.0 ± 0.0	-	0.0 ± 0.0	0.0 ± 0.0	-	0.0 ± 0.0	0.0 ± 0.0	-	0.0 ± 0.0	0.0 ± 0.0	-	0.0 ± 0.0	0.0 ± 0.0	-
SHf#20	10	*CmDMC1f*	0.0 ± 0.0	0.0 ± 0.0	-	0.0 ± 0.0	0.0 ± 0.0	-	0.0 ± 0.0	0.0 ± 0.0	-	0.0 ± 0.0	0.0 ± 0.0	-	0.0 ± 0.0	0.0 ± 0.0	-
Degrees of freedom (factor, error and total),<br>F-statistic and<br>p-value					-			2, 297, 299<br>1734.8<br><0.0001			2, 297, 299<br>75789.6<br><0.0001			2, 297, 299<br>19216.6<br><0.0001			-
Yamate-shiro (Control: YS)	0	all	21.3 ± 1.8	35.3 ± 1.2	37.6 ± 2.2	189.5 ± 2.4	181.6 ± 2.9	51.1 ± 0.5	378.6 ± 2.4	77.2 ± 2.6	83.1 ± 0.5	187.9 ± 1.6	124.6 ± 2.2	60.1 ± 0.5	0.0 ± 0.0	0.0 ± 0.0	-
YS#16	12	none	0.0 ± 0.0	0.0 ± 0.0	-	0.0 ± 0.0	0.0 ± 0.0	-	0.0 ± 0.0	0.0 ± 0.0	-	0.0 ± 0.0	0.0 ± 0.0	-	0.0 ± 0.0	0.0 ± 0.0	-
YSa#12	10	*CmDMC1a*	0.0 ± 0.0	0.0 ± 0.0	-	10.2 ± 1.34	21.0 ± 1.7	32.7 ± 3.2 **	22.2 ± 1.5	34.4±1.7	39.3 ± 1.5 **	9.0 ± 1.1	23.3 ± 1.7	27.8 ± 2.6 **	0.0 ± 0.0	0.0 ± 0.0	-
YSb#13	10	*CmDMC1b*	0.0 ± 0.0	0.0 ± 0.0	-	11.3 ± 1.2	23.3 ± 1.4	32.5 ± 2.4 **	19.2 ± 1.3	32.5±1.5	37.1 ± 1.5 **	9.5 ± 1.6	25.9 ± 2.2	26.8 ± 3.1 **	0.0 ± 0.0	0.0 ± 0.0	-
YSc#27	10	*CmDMC1c*	0.0 ± 0.0	0.0 ± 0.0	-	0.0 ± 0.0	0.0 ± 0.0	-	0.0 ± 0.0	0.0 ± 0.0	-	0.0 ± 0.0	0.0 ± 0.0	-	0.0 ± 0.0	0.0 ± 0.0	-
YSd#28	10	*CmDMC1d*	0.0 ± 0.0	0.0 ± 0.0	-	0.0 ± 0.0	0.0 ± 0.0	-	0.0 ± 0.0	0.0 ± 0.0	-	0.0 ± 0.0	0.0 ± 0.0	-	0.0 ± 0.0	0.0 ± 0.0	-
YSe#30	10	*CmDMC1e*	0.0 ± 0.0	0.0 ± 0.0	-	0.0 ± 0.0	0.0 ± 0.0	-	0.0 ± 0.0	0.0 ± 0.0	-	0.0 ± 0.0	0.0 ± 0.0	-	0.0 ± 0.0	0.0 ± 0.0	-
YSf#32	10	*CmDMC1f*	0.0 ± 0.0	0.0 ± 0.0	-	0.0 ± 0.0	0.0 ± 0.0	-	0.0 ± 0.0	0.0 ± 0.0	-	0.0 ± 0.0	0.0 ± 0.0	-	0.0 ± 0.0	0.0 ± 0.0	-
Degrees of freedom (factor, error and total),<br>F-statistic and<br>p-value					-			2, 297, 299<br>2193.1<br><0.0001			2, 297, 299<br>58547.5<br><0.0001			2, 297, 299<br>7352.2<br><0.0001			-

VP: viable pollen grains; AP: aborted pollen grains.

Red- and green-stained pollen grains were judged to be viable (A) and aborted (B), respectively, according to Alexander (1969).

Each value in (A) and (B) represents the mean of pollen grains per anther ± SE; in (C) a mean percentage of viable pollen grains [C = B / (A + B) x 100] per anther ± SE.

Each mean of percentages of viable or aborted pollen grains in each head flower in (C) and (D) was arcsine-transformed prior to analysis by ANOVA.

One hundred head flowers each and about 10 receptive tubular florets from each head flower of transgenic lines and non-transgenic control were used.

** significant at 1% level by ANOVA.

Supplementary Table S9. Evaluation of female sterility by crossing between transgenic lines/non-transformed controls and their wild relatives.

Line	No. of mutated alleles	Name of non-mutated gene(s)	Scientific name of pollen parent	10 °C			15 °C			20 °C			25 °C			30 °C		
				No. of VS (A)	No. of AS (B)	Ratio of VS (C in %)	No. of VS (A)	No. of AS (B)	Ratio of VS (C in %)	No. of VS (A)	No. of AS (B)	Ratio of VS (C in %)	No. of VS (A)	No. of AS (B)	Ratio of VS (C in %)	No. of VS (A)	No. of AS (B)	Ratio of VS (C in %)
Shuho-no-chikara (Control: SH)	0	all		0.0 ± 0.0	10.0 ± 0.0	0.0 ± 0.0	1.1 ± 0.2	8.9 ± 0.2	11.2 ± 1.8 ^{a}	7.0 ± 0.2	3.0 ± 0.2	70.2 ± 1.8 ^{a}	2.9 ± 0.2	7.0 ± 0.2	29.4 ± 2.2 ^{a}	2.2 ± 0.2	7.8 ± 0.2	22.2 ± 2.0 ^{a}
SH#12	12	none		0.0 ± 0.0	10.0 ± 0.0	0.0 ± 0.0	0.0 ± 0.0	10.0 ± 0.0	0.0 ± 0.0 ^{b}	0.0 ± 0.0	10.0 ± 0.0	0.0 ± 0.0 ^{b}	0.0 ± 0.0	10.0 ± 0.0	0.0 ± 0.0 ^{b}	0.0 ± 0.0	10.0 ± 0.0	0.0 ± 0.0 ^{b}
SHa#13	10	*CmDMC1a*		0.0 ± 0.0	10.0 ± 0.0	0.0 ± 0.0	0.2 ± 0.1	9.8 ± 0.1	1.5 ± 0.8 ^{c}	0.7 ± 0.1	9.3 ± 0.0	6.9 ± 1.3 ^{c}	0.0 ± 0.0	10.0 ± 0.0	0.0 ± 0.0 ^{b}	0.0 ± 0.0	10.0 ± 0.0	0.0 ± 0.0 ^{b}
SHb#14	10	*CmDMC1b*	*Ch. wakasaense*	0.0 ± 0.0	10.0 ± 0.0	0.0 ± 0.0	0.1 ± 0.1	9.9 ± 0.0	1.2 ± 0.7 ^{c}	0.6 ± 0.1	9.4 ± 0.1	6.1 ± 0.7 ^{d}	0.0 ± 0.0	10.0 ± 0.0	0.0 ± 0.0 ^{b}	0.0 ± 0.0	10.0 ± 0.0	0.0 ± 0.0 ^{b}
SHc#15	10	*CmDMC1c*		0.0 ± 0.0	10.0 ± 0.0	0.0 ± 0.0	0.0 ± 0.0	10.0 ± 0.1	0.0 ± 0.0 ^{b}	0.1 ± 0.1	9.9 ± 0.1	1.0 ± 0.8 ^{e}	0.0 ± 0.0	10.0 ± 0.0	0.0 ± 0.0 ^{b}	0.0 ± 0.0	10.0 ± 0.0	0.0 ± 0.0 ^{b}
SHd#16	10	*CmDMC1d*		0.0 ± 0.0	10.0 ± 0.0	0.0 ± 0.0	0.0 ± 0.0	10.0 ± 0.0	0.0 ± 0.0 ^{b}	0.1 ± 0.1	9.9 ± 0.1	1.1 ± 0.7 ^{e}	0.0 ± 0.0	10.0 ± 0.0	0.0 ± 0.0 ^{b}	0.0 ± 0.0	10.0 ± 0.0	0.0 ± 0.0 ^{b}
SHe#19	10	*CmDMC1e*		0.0 ± 0.0	10.0 ± 0.0	0.0 ± 0.0	0.0 ± 0.0	10.0 ± 0.0	0.0 ± 0.0 ^{b}	0.1 ± 0.1	9.9 ± 0.1	1.2 ± 0.8 ^{e}	0.0 ± 0.0	10.0 ± 0.0	0.0 ± 0.0 ^{b}	0.0 ± 0.0	10.0 ± 0.0	0.0 ± 0.0 ^{b}
SHf#20	10	*CmDMC1f*		0.0 ± 0.0	10.0 ± 0.0	0.0 ± 0.0	0.0 ± 0.0	10.0 ± 0.0	0.0 ± 0.0 ^{b}	0.1 ± 0.1	9.9 ± 0.1	1.2 ± 0.7 ^{e}	0.0 ± 0.0	10.0 ± 0.0	0.0 ± 0.0 ^{b}	0.0 ± 0.0	10.0 ± 0.0	0.0 ± 0.0 ^{b}
Degrees of freedom (factor, error and total), F-statistic and p-value						-			7, 792, 799<br>2763.9<br><0.0001			7, 792, 799<br>51591.9<br><0.0001			7, 792, 799<br>22719.3<br><0.0001			7, 792, 799<br>1545.6<br><0.0001
Shuho-no-chikara (Control: SH)	0	all		0.0 ± 0.0	10.0 ± 0.0	0.0 ± 0.0	1.0 ± 0.2	9.0 ± 0.2	10.2 ± 2.2 ^{a}	8.0 ± 0.2	2.0 ± 0.2	79.6 ± 2.4 ^{a}	3.5 ± 0.2	6.5 ± 0.2	34.6 ± 2.1 ^{a}	2.4 ± 0.2	7.6 ± 0.2	23.6 ± 2.1 ^{a}
SH#12	12	none		0.0 ± 0.0	10.0 ± 0.0	0.0 ± 0.0	0.0 ± 0.0	10.0 ± 0.0	0.0 ± 0.0 ^{b}	0.0 ± 0.0	10.0 ± 0.0	0.0 ± 0.0 ^{b}	0.0 ± 0.0	10.0 ± 0.0	0.0 ± 0.0 ^{b}	0.0 ± 0.0	10.0 ± 0.0	0.0 ± 0.0 ^{b}
SHa#13	10	*CmDMC1a*		0.0 ± 0.0	10.0 ± 0.0	0.0 ± 0.0	0.2 ± 0.1	9.8 ± 0.1	2.0 ± 1.0 ^{c}	0.6 ± 0.1	9.4 ± 0.1	6.2 ± 1.0 ^{c}	0.0 ± 0.0	10.0 ± 0.0	0.0 ± 0.0 ^{b}	0.0 ± 0.0	10.0 ± 0.0	0.0 ± 0.0 ^{b}
SHb#14	10	*CmDMC1b*	*Ch. japonense*	0.0 ± 0.0	10.0 ± 0.0	0.0 ± 0.0	0.2 ± 0.1	9.8 ± 0.1	1.5 ± 0.7 ^{d}	0.7 ± 0.1	9.3 ± 0.1	6.5 ± 0.7 ^{c}	0.0 ± 0.0	10.0 ± 0.0	0.0 ± 0.0 ^{b}	0.0 ± 0.0	10.0 ± 0.0	0.0 ± 0.0 ^{b}
SHc#15	10	*CmDMC1c*		0.0 ± 0.0	10.0 ± 0.0	0.0 ± 0.0	0.0 ± 0.0	10.0 ± 0.0	0.0 ± 0.0 ^{b}	0.1 ± 0.1	9.9 ± 0.1	1.2 ± 0.5 ^{d}	0.0 ± 0.0	10.0 ± 0.0	0.0 ± 0.0 ^{b}	0.0 ± 0.0	10.0 ± 0.0	0.0 ± 0.0 ^{b}
SHd#16	10	*CmDMC1d*		0.0 ± 0.0	10.0 ± 0.0	0.0 ± 0.0	0.0 ± 0.0	10.0 ± 0.0	0.0 ± 0.0 ^{b}	0.1 ± 0.1	9.9 ± 0.1	0.9 ± 0.7 ^{d}	0.0 ± 0.0	10.0 ± 0.0	0.0 ± 0.0 ^{b}	0.0 ± 0.0	10.0 ± 0.0	0.0 ± 0.0 ^{b}
SHe#19	10	*CmDMC1e*		0.0 ± 0.0	10.0 ± 0.0	0.0 ± 0.0	0.0 ± 0.0	10.0 ± 0.0	0.0 ± 0.0 ^{b}	0.2 ± 0.1	9.8 ± 0.1	1.5 ± 0.8 ^{d}	0.0 ± 0.0	10.0 ± 0.0	0.0 ± 0.0 ^{b}	0.0 ± 0.0	10.0 ± 0.0	0.0 ± 0.0 ^{b}
SHf#20	10	*CmDMC1f*		0.0 ± 0.0	10.0 ± 0.0	0.0 ± 0.0	0.0 ± 0.0	10.0 ± 0.0	0.0 ± 0.0 ^{b}	0.2 ± 0.1	9.8 ± 0.1	1.5 ± 0.8 ^{d}	0.0 ± 0.0	10.0 ± 0.0	0.0 ± 0.0 ^{b}	0.0 ± 0.0	10.0 ± 0.0	0.0 ± 0.0 ^{b}
Degrees of freedom (factor, error and total), F-statistic and p-value						-			7, 792, 799<br>1545.6<br><0.0001			7, 792, 799<br>38483.5<br><0.0001			7, 792, 799<br>25130.8<br><0.0001			7, 792, 799<br>12260.9<br><0.0001
Shuho-no-chikara (Control: SH)	0	all		0.0 ± 0.0	10.0 ± 0.0	0.0 ± 0.0	0.0 ± 0.0	10.0 ± 0.0	0.0 ± 0.0	0.0 ± 0.0	10.0 ± 0.0	0.0 ± 0.0	0.0 ± 0.0	10.0 ± 0.0	0.0 ± 0.0	0.0 ± 0.0	10.0 ± 0.0	0.0 ± 0.0
SH#12	12	none		0.0 ± 0.0	10.0 ± 0.0	0.0 ± 0.0	0.0 ± 0.0	10.0 ± 0.0	0.0 ± 0.0	0.0 ± 0.0	10.0 ± 0.0	0.0 ± 0.0	0.0 ± 0.0	10.0 ± 0.0	0.0 ± 0.0	0.0 ± 0.0	10.0 ± 0.0	0.0 ± 0.0
SHa#13	10	*CmDMC1a*		0.0 ± 0.0	10.0 ± 0.0	0.0 ± 0.0	0.0 ± 0.0	10.0 ± 0.0	0.0 ± 0.0	0.0 ± 0.0	10.0 ± 0.0	0.0 ± 0.0	0.0 ± 0.0	10.0 ± 0.0	0.0 ± 0.0	0.0 ± 0.0	10.0 ± 0.0	0.0 ± 0.0
SHb#14	10	*CmDMC1b*	self	0.0 ± 0.0	10.0 ± 0.0	0.0 ± 0.0	0.0 ± 0.0	10.0 ± 0.0	0.0 ± 0.0	0.0 ± 0.0	10.0 ± 0.0	0.0 ± 0.0	0.0 ± 0.0	10.0 ± 0.0	0.0 ± 0.0	0.0 ± 0.0	10.0 ± 0.0	0.0 ± 0.0
SHc#15	10	*CmDMC1c*		0.0 ± 0.0	10.0 ± 0.0	0.0 ± 0.0	0.0 ± 0.0	10.0 ± 0.0	0.0 ± 0.0	0.0 ± 0.0	10.0 ± 0.0	0.0 ± 0.0	0.0 ± 0.0	10.0 ± 0.0	0.0 ± 0.0	0.0 ± 0.0	10.0 ± 0.0	0.0 ± 0.0
SHd#16	10	*CmDMC1d*		0.0 ± 0.0	10.0 ± 0.0	0.0 ± 0.0	0.0 ± 0.0	10.0 ± 0.0	0.0 ± 0.0	0.0 ± 0.0	10.0 ± 0.0	0.0 ± 0.0	0.0 ± 0.0	10.0 ± 0.0	0.0 ± 0.0	0.0 ± 0.0	10.0 ± 0.0	0.0 ± 0.0
SHe#19	10	*CmDMC1e*		0.0 ± 0.0	10.0 ± 0.0	0.0 ± 0.0	0.0 ± 0.0	10.0 ± 0.0	0.0 ± 0.0	0.0 ± 0.0	10.0 ± 0.0	0.0 ± 0.0	0.0 ± 0.0	10.0 ± 0.0	0.0 ± 0.0	0.0 ± 0.0	10.0 ± 0.0	0.0 ± 0.0
SHf#20	10	*CmDMC1f*		0.0 ± 0.0	10.0 ± 0.0	0.0 ± 0.0	0.0 ± 0.0	10.0 ± 0.0	0.0 ± 0.0	0.0 ± 0.0	10.0 ± 0.0	0.0 ± 0.0	0.0 ± 0.0	10.0 ± 0.0	0.0 ± 0.0	0.0 ± 0.0	10.0 ± 0.0	0.0 ± 0.0
Yamate-shiro (Control: YS)	0	all		0.0 ± 0.0	10.0 ± 0.0	0.0 ± 0.0	1.2 ± 0.2	8.8 ± 0.2	12.2 ± 1.8 ^{a}	6.8 ± 0.2	3.2 ± 0.2	68.4 ± 1.9 ^{a}	3.1 ± 0.2	6.9 ± 0.2	31.2 ± 2.1 ^{a}	1.9 ± 0.2	8.1 ± 0.2	19.2 ± 2.1 ^{a}
YS#16	12	none		0.0 ± 0.0	10.0 ± 0.0	0.0 ± 0.0	0.0 ± 0.0	10.0 ± 0.0	0.0 ± 0.0 ^{b}	0.0 ± 0.0	10.0 ± 0.0	0.0 ± 0.0 ^{b}	0.0 ± 0.0	10.0 ± 0.0	0.0 ± 0.0 ^{b}	0.0 ± 0.0	10.0 ± 0.0	0.0 ± 0.0 ^{b}
YSa#12	10	*CmDMC1a*		0.0 ± 0.0	10.0 ± 0.0	0.0 ± 0.0	0.1 ± 0.1	9.9 ± 0.1	1.2 ± 0.7 ^{c}	0.6 ± 0.1	9.4 ± 0.1	6.2 ± 0.9 ^{c}	0.0 ± 0.0	10.0 ± 0.0	0.0 ± 0.0 ^{b}	0.0 ± 0.0	10.0 ± 0.0	0.0 ± 0.0 ^{b}
YSb#13	10	*CmDMC1b*	*Ch. wakasaense*	0.0 ± 0.0	10.0 ± 0.0	0.0 ± 0.0	0.1 ± 0.1	9.9 ± 0.1	1.0 ± 0.8 ^{c}	0.7 ± 0.1	9.3 ± 0.1	6.9 ± 1.2 ^{d}	0.0 ± 0.0	10.0 ± 0.0	0.0 ± 0.0 ^{b}	0.0 ± 0.0	10.0 ± 0.0	0.0 ± 0.0 ^{b}
YSc#27	10	*CmDMC1c*		0.0 ± 0.0	10.0 ± 0.0	0.0 ± 0.0	0.0 ± 0.0	0.0 ± 0.0	0.0 ± 0.0 ^{b}	0.1 ± 0.1	9.9 ± 0.1	1.1 ± 0.7 ^{e}	0.0 ± 0.0	10.0 ± 0.0	0.0 ± 0.0 ^{b}	0.0 ± 0.0	10.0 ± 0.0	0.0 ± 0.0 ^{b}
YSd#30	10	*CmDMC1d*		0.0 ± 0.0	10.0 ± 0.0	0.0 ± 0.0	0.0 ± 0.0	0.0 ± 0.0	0.0 ± 0.0 ^{b}	0.1 ± 0.1	9.9 ± 0.1	1.0 ± 0.6 ^{e}	0.0 ± 0.0	10.0 ± 0.0	0.0 ± 0.0 ^{b}	0.0 ± 0.0	10.0 ± 0.0	0.0 ± 0.0 ^{b}
YSe#28	10	*CmDMC1e*		0.0 ± 0.0	10.0 ± 0.0	0.0 ± 0.0	0.0 ± 0.0	0.0 ± 0.0	0.0 ± 0.0 ^{b}	0.1 ± 0.1	9.9 ± 0.1	0.9 ± 0.7 ^{e}	0.0 ± 0.0	10.0 ± 0.0	0.0 ± 0.0 ^{b}	0.0 ± 0.0	10.0 ± 0.0	0.0 ± 0.0 ^{b}
YSf#32	10	*CmDMC1f*		0.0 ± 0.0	10.0 ± 0.0	0.0 ± 0.0	0.0 ± 0.0	0.0 ± 0.0	0.0 ± 0.0 ^{b}	0.1 ± 0.1	9.9 ± 0.1	0.8 ± 0.5 ^{e}	0.0 ± 0.0	10.0 ± 0.0	0.0 ± 0.0 ^{b}	0.0 ± 0.0	10.0 ± 0.0	0.0 ± 0.0 ^{b}
Degrees of freedom (factor, error and total), F-statistic and p-value						-			7, 792, 799<br>3259.76<br><0.0001			7, 792, 799<br>48463.8<br><0.0001			7, 792, 799<br>24258.1<br><0.0001			7, 792, 799<br>8272.2<br><0.0001
Yamate-shiro (Control: YS)	0	all		0.0 ± 0.0	10.0 ± 0.0	0.0 ± 0.0	1.2 ± 0.1	8.8 ± 0.1	12.3 ± 1.2 ^{a}	7.7 ± 0.2	2.3 ± 0.2	76.6 ± 2.4 ^{a}	3.3 ± 2.1	6.7 ± 2.3	33.2 ± 2.1 ^{a}	2.1 ± 2.3	7.9 ± 3.2	21.3 ± 2.3 ^{a}
YS#16	12	none		0.0 ± 0.0	10.0 ± 0.0	0.0 ± 0.0	0.0 ± 0.0	10.0 ± 0.0	0.0 ± 0.0 ^{b}	0.0 ± 0.0	10.0 ± 0.0	0.0 ± 0.0 ^{b}	0.0 ± 0.0	10.0 ± 0.0	0.0 ± 0.0 ^{b}	0.0 ± 0.0	10.0 ± 0.0	0.0 ± 0.0 ^{b}
YSa#12	10	*CmDMC1a*		0.0 ± 0.0	10.0 ± 0.0	0.0 ± 0.0	0.2 ± 0.1	9.8 ± 0.1	1.6 ± 1.1 ^{c}	0.7 ± 0.1	9.3 ± 0.1	7.0 ± 1.1 ^{c}	0.0 ± 0.0	10.0 ± 0.0	0.0 ± 0.0 ^{b}	0.0 ± 0.0	10.0 ± 0.0	0.0 ± 0.0 ^{b}
YSb#13	10	*CmDMC1b*	*Ch. japonense*	0.0 ± 0.0	10.0 ± 0.0	0.0 ± 0.0	0.1 ± 0.1	9.9 ± 0.1	1.2 ± 0.7 ^{d}	0.7 ± 0.1	9.3 ± 0.1	7.1 ± 1.0 ^{c}	0.0 ± 0.0	10.0 ± 0.0	0.0 ± 0.0 ^{b}	0.0 ± 0.0	10.0 ± 0.0	0.0 ± 0.0 ^{b}
YSc#27	10	*CmDMC1c*		0.0 ± 0.0	10.0 ± 0.0	0.0 ± 0.0	0.0 ± 0.0	10.0 ± 0.0	0.0 ± 0.0 ^{b}	0.1 ± 0.1	9.9 ± 0.1	0.8 ± 0.6 ^{d}	0.0 ± 0.0	10.0 ± 0.0	0.0 ± 0.0 ^{b}	0.0 ± 0.0	10.0 ± 0.0	0.0 ± 0.0 ^{b}
YSd#30	10	*CmDMC1d*		0.0 ± 0.0	10.0 ± 0.0	0.0 ± 0.0	0.0 ± 0.0	10.0 ± 0.0	0.0 ± 0.0 ^{b}	0.1 ± 0.1	9.9 ± 0.1	1.0 ± 0.7 ^{d}	0.0 ± 0.0	10.0 ± 0.0	0.0 ± 0.0 ^{b}	0.0 ± 0.0	10.0 ± 0.0	0.0 ± 0.0 ^{b}
YSe#28	10	*CmDMC1e*		0.0 ± 0.0	10.0 ± 0.0	0.0 ± 0.0	0.0 ± 0.0	10.0 ± 0.0	0.0 ± 0.0 ^{b}	0.1 ± 0.1	9.9 ± 0.1	1.2 ± 0.9 ^{d}	0.0 ± 0.0	10.0 ± 0.0	0.0 ± 0.0 ^{b}	0.0 ± 0.0	10.0 ± 0.0	0.0 ± 0.0 ^{b}
YSf#32	10	*CmDMC1f*		0.0 ± 0.0	10.0 ± 0.0	0.0 ± 0.0	0.0 ± 0.0	10.0 ± 0.0	0.0 ± 0.0 ^{b}	0.1 ± 0.1	9.9 ± 0.1	0.9 ± 0.8 ^{d}	0.0 ± 0.0	10.0 ± 0.0	0.0 ± 0.0 ^{b}	0.0 ± 0.0	10.0 ± 0.0	0.0 ± 0.0 ^{b}
Degrees of freedom (factor, error and total), F-statistic and p-value				-	-	-	-	-	7, 792, 799<br>4522.7<br><0.0001	-	-	7, 792, 799<br>36785.8<br><0.0001	-	-	7, 792, 799<br>23077.1<br><0.0001	-	-	7, 792, 799<br>8113.8<br><0.0001
Yamate-shiro (Control: YS)	0	all		0.0 ± 0.0	10.0 ± 0.0	0.0 ± 0.0	0.0 ± 0.0	10.0 ± 0.0	0.0 ± 0.0	0.0 ± 0.0	10.0 ± 0.0	0.0 ± 0.0	0.0 ± 0.0	10.0 ± 0.0	0.0 ± 0.0	0.0 ± 0.0	10.0 ± 0.0	0.0 ± 0.0
YS#16	12	none		0.0 ± 0.0	10.0 ± 0.0	0.0 ± 0.0	0.0 ± 0.0	10.0 ± 0.0	0.0 ± 0.0	0.0 ± 0.0	10.0 ± 0.0	0.0 ± 0.0	0.0 ± 0.0	10.0 ± 0.0	0.0 ± 0.0	0.0 ± 0.0	10.0 ± 0.0	0.0 ± 0.0
YSa#12	10	*CmDMC1a*		0.0 ± 0.0	10.0 ± 0.0	0.0 ± 0.0	0.0 ± 0.0	10.0 ± 0.0	0.0 ± 0.0	0.0 ± 0.0	10.0 ± 0.0	0.0 ± 0.0	0.0 ± 0.0	10.0 ± 0.0	0.0 ± 0.0	0.0 ± 0.0	10.0 ± 0.0	0.0 ± 0.0
YSb#13	10	*CmDMC1b*	self	0.0 ± 0.0	10.0 ± 0.0	0.0 ± 0.0	0.0 ± 0.0	10.0 ± 0.0	0.0 ± 0.0	0.0 ± 0.0	10.0 ± 0.0	0.0 ± 0.0	0.0 ± 0.0	10.0 ± 0.0	0.0 ± 0.0	0.0 ± 0.0	10.0 ± 0.0	0.0 ± 0.0
YSc#27	10	*CmDMC1c*		0.0 ± 0.0	10.0 ± 0.0	0.0 ± 0.0	0.0 ± 0.0	10.0 ± 0.0	0.0 ± 0.0	0.0 ± 0.0	10.0 ± 0.0	0.0 ± 0.0	0.0 ± 0.0	10.0 ± 0.0	0.0 ± 0.0	0.0 ± 0.0	10.0 ± 0.0	0.0 ± 0.0
YSd#30	10	*CmDMC1d*		0.0 ± 0.0	10.0 ± 0.0	0.0 ± 0.0	0.0 ± 0.0	10.0 ± 0.0	0.0 ± 0.0	0.0 ± 0.0	10.0 ± 0.0	0.0 ± 0.0	0.0 ± 0.0	10.0 ± 0.0	0.0 ± 0.0	0.0 ± 0.0	10.0 ± 0.0	0.0 ± 0.0
YSe#28	10	*CmDMC1e*		0.0 ± 0.0	10.0 ± 0.0	0.0 ± 0.0	0.0 ± 0.0	10.0 ± 0.0	0.0 ± 0.0	0.0 ± 0.0	10.0 ± 0.0	0.0 ± 0.0	0.0 ± 0.0	10.0 ± 0.0	0.0 ± 0.0	0.0 ± 0.0	10.0 ± 0.0	0.0 ± 0.0
YSf#32	10	*CmDMC1f*		0.0 ± 0.0	10.0 ± 0.0	0.0 ± 0.0	0.0 ± 0.0	10.0 ± 0.0	0.0 ± 0.0	0.0 ± 0.0	10.0 ± 0.0	0.0 ± 0.0	0.0 ± 0.0	10.0 ± 0.0	0.0 ± 0.0	0.0 ± 0.0	10.0 ± 0.0	0.0 ± 0.0

VS: viable F_1 seeds; AS: aborted F_1 seeds.

The F_1 seeds with germination ability were judged to be viable (A) and ones with no germination ability were aborted (B), respectively.

Each value in (A) and (B) represents the mean of F1 seeds per head flower ± SE; in (C) a mean percentage of viable F_1 seeds [C = A / (A + B) x 100] per head flower ± SE.

One head flowers each and about 10 receptive stigmas from each tubular flower of transgenic lines and non-transgenic control were used.

The mean percentage of viable F_1 seeds were arcsine-transformed prior to Tukey-Kramer's HSD.

Values followed by the same letter are not significantly different at 1% level by Tukey-Kramer's HSD.

```
#a     1 ATGCAAGCACTCAAATCTGAAGATATGAGTCAATTACAGCTCGTTGAACGTGAAGAGATCGATGAGGAAGAAGATTTGTTTGAAGCGATTGATAAATTGA  100
#b     1 ATGCAAGCACTCAAATCTGAAGATATGAGTCAATTACAGCTCGTTGAACGTGAAGAGATCGATGAGGAAGAAGATTTGTTTGAAGCGATTGATAAATTGA  100
#c     1 ATGCAAGCACTCAAATCTGAAGATATGAGTCAATTACAGCTCGTTGAACGTGAAGAGATCGATGAGGAAGAAGATTTGCTTGAAGCGATTGATAAATTGA  100
#d     1 ATGCAAGCACTCAAATCTGAAGAAATGAGTCGATTACAGCTCGTTGAACGTGAAGAGATCGATGAGGAAGAAGATTTGTTTGAAGCGATTGATAAATTGA  100
#e     1 ATGCAAGCACTCAAATCTGAAGAAATGAGTCGATTACAGCTCGTTGAACGTGAAGAGATCGATGAGGAAGAAGATTTGTTTGAAGCGATTGATAAATTGA  100
#f     1 ATGCAAGCACTCAAATCTGAAGATATGAGTCAATTACAGCTCGTTGAACGTGAAGAGATCGATGAGGAAGAAGATTTGTTTGAAGCGATTGATAAATTGA  100

#a   101 CTGCTCATGGAATAAATGCTGGCGATGTGAAGAAGCTACAAGATGCAGGAATATATACCTGCAATGGCTTGATGATGCATACTAAGAAGAACTTGACTGG  200
#b   101 CTGCTCATGGAATAAATGCTGGCGATGTGAAGAAGCTACAAGATGCAGGAATATATACCTGCAATGGCTTGATGATGCACACTAAGAAGAACTTGACTGG  200
#c   101 CTGCTCATGGAATAAATGCTGGCGATGTGAAGAAGCTACAAGATGCAGGAATATATACCTGCAATGGCTTGATGATGCATACTAAGAAGAACTTGACTGG  200
#d   101 CTGCTCATGGAATAAATGCTGGCGATGTGAAGAAGCTACAAGATGCAGGAATATATACCTGCAATGGCTTGATGATGCACACTAAGAAGAACTTGACTGG  200
#e   101 CTGCTCATGGAATAAATGCTGGCGATGTGAAGAAGCTACAAGATGCAGGAATATATACCTGCAATGGCTTGATGATGCACACTAAGAAGAACTTGACTGG  200
#f   101 CTGCTCATGGAATAAATGCTGGCGATGTGAAGAAGCTACAAGATGCAGGAATATATACCTGCAATGGCTTGATGATGCACACTAAGAAGAACTTGACTGG  200

#a   201 CATAAAAGGTTTATCTGAGGCTAAAGTTGATAAAATCTGTGAAGCTGCTGAGAAGCTAGTGAATTATGGTTACATTACTGGAAGCGATGCTCTGCTCAAA  300
#b   201 CATAAAAGGACTATCTGAGGCTAAAGTTGATAAAATCTGTGAAGCTGCTGAGAAGCTAGTGAATTATGGTTACATTACTGGAAGCGATGCTCTGCTCAAA  300
#c   201 CATAAAAGGTTTATCTGAGGCTAAAGTTGATAAAATCTGTGAAGCTGCTGAGAAGCTAGTGAATTATGGTTACATTACTGGAAGCGATGCTCTGCTCAAA  300
#d   201 CATAAAAGGTTTATCTGAGGCTAAAGTTGATAAAATCTGTGAAGCTGCTGAGAAGCTAGTGAATTATGGCTACATTACTGGAAGCGATGCTCTGCTCAAA  300
#e   201 CATAAAAGGTTTATCTGAGGCTAAAGTTGATAAAATCTGTGAAGCTGCTGAGAAGCTAGTGAATTTTGGCTACATTACTGGAAGCGATGCTCTGCTCAAA  300
#f   201 CATAAAAGGACTATCTGAGGCTAAAGTTGATAAAATCTGTGAAGCTGCTGAGAAGCTAGTGAATTATGGCTACATTACTGGAAGCGATGCTCTGCTCAAA  300
                                                   DMC1-RNAi F1

#a   301 AGAAAGGCTGTTGTCAAAATCACTACTGGAAGTCAAGCTCTTGATGAGCTTTTGGGAGGTGGTATTGAAACTCTGCAAATCACGGAAGCTTTTGGCGAGT  400
#b   301 AGAAAGGCTGTTGTCAAAATCACTACTGGAAGTCAAGCTCTTGATGAGCTGTTGGGAGGTGGTATTGAAACTCTGCAAATCACCGAAGCTTTTGGCGAGT  400
#c   301 AGAAAGGCTGTTGTCAAAATCACTACTGGAAGTCAAGCTCTTGATGAGCTTTTGGGAGGTGGTATTGAAACTCTGCAAATCACGGAAGCTTTTGGCGAGT  400
#d   301 AGAAAGGCTGTTGTCAAAATCACTACTGGAAGTCAAGCTCTTGATGAGCTGTTGGGAGGTGGTATTGAAACTATGCAAATCACCGAAGCTTTTGGCGAGT  400
#e   301 AGAAAGGCTGTTGTCAAAATCACTACTGGAAGTCAAGCTCTTGATGAGCTGTTGGGAGGTGGTATTGAAACTATGCAAATCACCGAAGCTTTTGGCGAGT  400
#f   301 AGAAAGGCTGTTGTCAAAATCACTACTGGAAGTCAAGCTCTTGATGAGCTGTTGGGAGGTGGTATTGAAACTATGCAAATCACCGAAGCTTTTGGTGAGT  400

#a   401 TTAGGTCTGGAAAGACACAGCTGGCTCATACTCTTTGCGTTTCAACACAGCTTCCTACTAGCATGAAAGGTGGGAATGGAAGGTTGCTTATATTGATAC  500
#b   401 TTAGGTCTGGAAAGACACAGCTGGCTCATACTCTTTGCGTTTCAACACAGCTTCCTACTAGCATGAAAAGTGGAAATGGAAGGTTGCTTATATTGATAC  500
#c   401 TTAGGTCTGGAAAGACACAGCTGGCTCATACTCTTTGCGTTTCAACACAGCTTCCTACTAGCATGAAAGGTGGAAATGGAAGGTTGCTTATATTGATAC  500
#d   401 TTAGGTGTGGAAAGACACAGCTGGCTCATACTCTTTGCGTTTCAACACAGCTTCCTACTAGCATGAAAGGTGGGAATGGAAGGTTGCTTATATTGATAC  500
#e   401 TTAGGTGTGGAAAGACACAGCTGGCTCATACTCTTTGCGTTTCAACACAGCTTCCTACTAGCATGAAAGGTGGGAATGGAAGGTTGCTTATATTGATAC  500
#f   401 TTAGGTCTGGAAAGACACAGCTGGCTCATACTCTTTGCGTTTCAACACAGCTTCCTACTAGCATGAAAGGTGGGAATGGAAGGTTGCTTATATTGATAC  500

#a   501 TGTGGGAACTTTCCGACCTGATCGTATTGTACCCATTGCTGAAAGGTTTGGCATGGATCCTGGAGCTGTACTTGATAATATTATCTATGCACGTGCTTAC  600
#b   501 TGAGGGAACTTTCCGACCTGATCGCATTGTACCCATTGCTGAAAGGTTTGGCATGGATCCTGGAGCTGTACTTGATAATATTATCTATGCACGTGCTTAC  600
#c   501 TGAGGGAACTTTCCGACCTGATCGTATTGTACCCATTGCTGAAAGGTTTGGCATGGATCCTGGAGCTGTACTTGATAATATTATCTATGCACGTGCTTAC  600
#d   501 TGAGGGAACTTTCCGACCTGATCGTATTGTACCCATTGCTGAAAGGTTTGGCATGGATCCTGGAGCTGTACTTGATAATATTATCTATGCACGTGCTTAC  600
#e   501 TGAGGGAACTTTCCGACCTGATCGTATTGTACCCATTGCTGAAAGGTTTGGCATGGATCCTGGAGCTGTACTTGATAATATTATCTATGCACGTGCTTAC  600
#f   501 TGAGGGAACTTTCCGACCTGATCGTATTGTACCCATTGCTGAAAGGTTTGGCATGGATCCTGGAGCTGTACTTGATAATATTATCTATGCACGTGCTTAC  600
                                                                  DMC1-gF1
                                                                                  Walker B
#a   601 ACATACGAGCATCAATACAACCTGCTCCTTGGTTTGGCAGCAAAGATGGCTGAAGAACCTTTCAGACTACTGATTGTTGATTCTGTCATTGCTCTGTTCC  700
#b   601 ACATACGAGCATCAATACAACCTGCTCCTTGGTTTGGCAGCAAAGATGGCTGAAGAACCTTTCAGACTACTGATTGTTGATTCTGTCATTGCTCTGTTCC  700
#c   601 ACATACGAGCATCAATACAACCTGCTCCTTGGTTTGGCAGCAAAGATGGCTGAAGAACCTTTCAGACTACTGATTGTTGATTCTGTCATTGCTCTGTTCC  700
#d   601 ACATACGAGCATCAATACAACCTGCTCCTTGGTTTGGCAGCAAAGATGGCTGAAGAACCTTTCAGACTACTGATTGTTGATTCTGTCATTGCTCTGTTCC  700
#e   601 ACATACGAGCATCAATACAACCTGCTCCTTGGTTTGGCAGCAAAGATGGCTGAAGAACCTTTCAGACTACTGATTGTTGATTCTGTCATTGCTCTGTTCC  700
#f   601 ACATACGAGCATCAATACAACCTGCTCCTTGGTTTGGCAGCAAAGATGGCTGAAGAACCTTTCAGACTACTGATTGTTGATTCTGTCATTGCTCTGTTCC  700
                        TAL-L                                 TAL-R

#a   701 GAGTTGACTTCACTGGAAGAGGAGAACTTGCAGAGCGCCAGCAAAAGCTGGCTCAGATGCTCTCCCGATTGACCAAGATTGCTGAAGAATTTAACGTTGC  800
#b   701 GAGTTGACTTCACTGGAAGAGGAGAACTTGCAGAGCGCCAGCAAAAGCTGGCTCAGATGCTCTCCCGATTGACCAAGATTGCTGAAGAATTTAACGTTGC  800
#c   701 GAGTTGACTTCACTGGAAGGGGAGAACTTGCAGAGCGCCAGCAAAAGCTGGCTCAGATGCTCTCCCGATTGACCAAGATTGCTGAAGAATTTAACGTTGC  800
#d   701 GAGTTGACTTCACTGGAAGAGGAGAACTTGCAGATCGCCAGCAAAAGCTGGCTCAGATGCTCTCCCGATTGACCAAGATTGCTGAAGAATTTAACGTTGC  800
#e   701 GAGTTGACTTCACTGGAAGAGGAGAACTTGCAGATCGCCAGCAAAAGCTGGCTCAGATGCTCTCCCGATTGACCAAGATTGCTGAAGAATTTAACGTTGC  800
#f   701 GAGTTGACTTCACTGGAAGAGGAGAACTTGCAGAGCGCCAGCAAAAGCTGGCTCAGATGCTCTCCCGATTGACCAAGATTGCTGAAGAATTTAACGTTGC  800
                        DMC1-gR1

#a   801 AGTGTATATGACAAACCAAGTGATAGCTGATCCAGGTGGTGGAGTATTCATCTCAGATCCAAAGAAACCAGCAGGAGGGCATGTTCTTGCTCATGCAGCC  900
#b   801 AGTGTATATGACAAACCAAGTGATAGCTGATCCAGGTGGTGGAGTATTCATCTCAGATCCAAAGAAACCAGCAGGAGGGCATGTTCTTGCTCATGCAGCC  900
#c   801 AGTGTATATGACAAACCAAGTGATAGCTGATCCAGGTGGTGGAGTATTCATCTCAGATCCAAAGAAACCAGCAGGAGGGCATGTTCTTGCTCATGCAGCC  900
#d   801 AGTGTATATGACAAACCAAGTGATAGCTGATCCAGGTGGTGGAGTATTCATCTCAGATCCAAAGAAACCAGCAGGAGGGCATGTTCTTGCTCATGCAGCC  900
#e   801 AGTGTATATGACAAACCAAGTGATAGCTGATCCAGGTGGTGGAGTATTCATCTCAGATCCAAAGAAACCAGCAGGAGGGCATGTTCTTGCTCATGCAGCC  900
#f   801 AGTGCATATGACAAACCAAGTGATAGCTGATCCAGGTGGTGGAGTATTCATCTCAGATCCAAAGAAACCAGCAGGAGGGCATGTTCTTGCTCATGCAGCC  900
          DMC1-RNAi R1

#a   901 ACTGTGAGATTGATGTTTAGGAAGGGCAAGGGTGAACAGCGTGTTTGCAAAGTGTTTGATGCACCAAATCTCCCAGAGTCTGAAGCAATTTTTCAGATTA 1000
#b   901 ACTGTGAGATTGATGTTTAGGAAGGGCAAGGGTGAACAGCGTGTTTGCAAAGTGTTTGATGCACCAAATCTCCCAGAGTCTGAAGCAATTTTTCAGACTA 1000
#c   901 ACTGTGAGATTGATGTTTAGGAAGGGCGAGGGTGAACAGCGTGTTTGCAAAGTGTTTGATGCACCAAATCTCCCAGAGTCTGAAGCAATTTTTCAGATTA 1000
#d   901 ACTGTGAGATTGATGTTTAGGAAGGGCAAGGGTGAACAGCGTGTTTGCAAAGTGTTTGATGCACCAAATCTCCCAGAGTCTGAAGCAATTTTTCAGATTA 1000
#e   901 ACTGTGAGATTGATGTTTAGGAAGGGCAAGGGTGAACAGCGTGTTTGCAAAGTGTTTGATGCACCAAATCTCCCAGAGTCTGAAGCAATTTTTCAGATTA 1000
#f   901 ACTGTGAGATTGATGTTTAGGAAGGGCAAGGGTGAACAGCGTGTTTGCAAAGTGTTTGATGCACCAAATCTCCCAGAGTCTGAAGCAATTTTTCAGATTA 1000

#a  1001  CAGGTGGTGGAATCGCAGATGCAAAGGACTGA  1032
#b  1001  CAGGTGGTGGAATCGCAGATGCAAAGGACTAA  1032
#c  1001  CAGGTGGTGGAATCGCAGATGCAAAGGACTGA  1032
#d  1001  CAGGTGGTGGAATCGCAGATGCAAAGGACTAA  1032
#e  1001  CAGGTGGTGGAATCGCAGATGCAAAGGACTAA  1032
#f  1001  CAGGTGGTGGAATCGCAGATGCAAAGGACTGA  1032
```

Supplementary Figure S1. Alignment of cDNA sequences for the six *CmDMC1* genes of *Chrysanthemum morifolium* cultivar 'Shuho-no-chikara'.

CmDMC1 cDNAs were isolated using rapid amplification of cDNA ends (RACE) and a cDNA library prepared from 'Shuho-no-chikara'. See Fig. 1 for explanations on the two primer pairs (DMC1-RNAi F1 and DMC1-RNAi R1, and DMC1-gF1 and DMC1-gR1).

```
#a    1 MQALKSEDMSQLQLVEREEIDEEEDLFEAIDKLTAHGINAGDVKKLQDAGIYTCNGLMMHTKKNLTGIKGLSEAKVDKICEAAEKLVNYGYITGSDALLK 100
#b    1 MQALKSEDMSQLQLVEREEIDEEEDLFEAIDKLTAHGINAGDVKKLQDAGIYTCNGLMMHTKKNLTGIKGLSEAKVDKICEAAEKLVNYGYITGSDALLK 100
#c    1 MQALKSEDMSQLQLVEREEIDEEEDLLEAIDKLTAHGINAGDVKKLQDAGIYTCNGLMMHTKKNLTGIKGLSEAKVDKICEAAEKLVNYGYITGSDALLK 100
#d    1 MQALKSEEMSRLQLVEREEIDEEEDLFEAIDKLTAHGINAGDVKKLQDAGIYTCNGLMMHTKKNLTGIKGLSEAKVDKICEAAEKLVNYGYITGSDALLK 100
#e    1 MQALKSEEMSRLQLVEREEIDEEEDLFEAIDKLTAHGINAGDVKKLQDAGIYTCNGLMMHTKKNLTGIKGLSEAKVDKICEAAEKLVNFGYITGSDALLK 100
#f    1 MQALKSEDMSQLQLVEREEIDEEEDLFEAIDKLTAHGINAGDVKKLQDAGIYTCNGLMMHTKKNLTGIKGLSEAKVDKICEAAEKLVNYGYITGSDALLK 100

#a  101 RKAVVKITTGSQALDELLGGGIETLQITEAFGEFRSGKTQLAHTLCVSTQLPTSMKGGNGKVAYIDTVGTFRPDRIVPIAERFGMDPGAVLDNIIYARAY 200
#b  101 RKAVVKITTGSQALDELLGGGIETLQITEAFGEFRSGKTQLAHTLCVSTQLPTSMKSGNGKVAYIDTEGTFRPDRIVPIAERFGMDPGAVLDNIIYARAY 200
#c  101 RKAVVKITTGSQALDELLGGGIETLQITEAFGEFRSGKTQLAHTLCVSTQLPTSMKGGNGKVAYIDTEGTFRPDRIVPIAERFGMDPGAVLDNIIYARAY 200
#d  101 RKAVVKITTGSQALDELLGGGIETMQITEAFGEFRCGKTQLAHTLCVSTQLPTSMKGGNGKVAYIDTEGTFRPDRIVPIAERFGMDPGAVLDNIIYARAY 200
#e  101 RKAVVKITTGSQALDELLGGGIETMQITEAFGEFRCGKTQLAHTLCVSTQLPTSMKGGNGKVAYIDTEGTFRPDRIVPIAERFGMDPGAVLDNIIYARAY 200
#f  101 RKAVVKITTGSQALDELLGGGIETMQITEAFGEFRSGKTQLAHTLCVSTQLPTSMKGGNGKVAYIDTEGTFRPDRIVPIAERFGMDPGAVLDNIIYARAY 200

                TAL-L        TAL-R
#a  201 TYEHQYNLLLGLAAKMAEEPFRLLIVDSVIALFRVDFTGRGELAERQQKLAQMLSRLTKIAEEFNVAVYMTNQVIADPGGGVFISDPKKPAGGHVLAHAA 300
#b  201 TYEHQYNLLLGLAAKMAEEPFRLLIVDSVIALFRVDFTGRGELAERQQKLAQMLSRLTKIAEEFNVAVYMTNQVIADPGGGVFISDPKKPAGGHVLAHAA 300
#c  201 TYEHQYNLLLGLAAKMAEEPFRLLIVDSVIALFRVDFTGRGELAERQQKLAQMLSRLTKIAEEFNVAVYMTNQVIADPGGGVFISDPKKPAGGHVLAHAA 300
#d  201 TYEHQYNLLLGLAAKMAEEPFRLLIVDSVIALFRVDFTGRGELADRQQKLAQMLSRLTKIAEEFNVAVYMTNQVIADPGGGVFISDPKKPAGGHVLAHAA 300
#e  201 TYEHQYNLLLGLAAKMAEEPFRLLIVDSVIALFRVDFTGRGELADRQQKLAQMLSRLTKIAEEFNVAVYMTNQVIADPGGGVFISDPKKPAGGHVLAHAA 300
#f  201 TYEHQYNLLLGLAAKMAEEPFRLLIVDSVIALFRVDFTGRGELAERQQKLAQMLSRLTKIAEEFNVAVHMTNQVIADPGGGVFISDPKKPAGGHVLAHAA 300
                                  Walker B

#a  301 TVRLMFRKGKGEQRVCKVFDAPNLPESEAIFQITGGGIADAKD* 343
#b  301 TVRLMFRKGKGEQRVCKVFDAPNLPESEAIFQTTGGGIADAKD* 343
#c  301 TVRLMFRKGEGEQRVCKVFDAPNLPESEAIFQITGGGIADAKD* 343
#d  301 TVRLMFRKGKGEQRVCKVFDAPNLPESEAIFQITGGGIADAKD* 343
#e  301 TVRLMFRKGKGEQRVCKVFDAPNLPESEAIFQITGGGIADAKD* 343
#f  301 TVRLMFRKGKGEQRVCKVFDAPNLPESEAIFQITGGGIADAKD* 343
```

Supplementary Figure S2. Amino acid sequences for the six *CmDMC1* gene products of *Chrysanthemum morifolium* cultivar ‘Shuho-no-chikara’.
Sequences were aligned with the DNASIS software as in Supplementary Fig. S1.

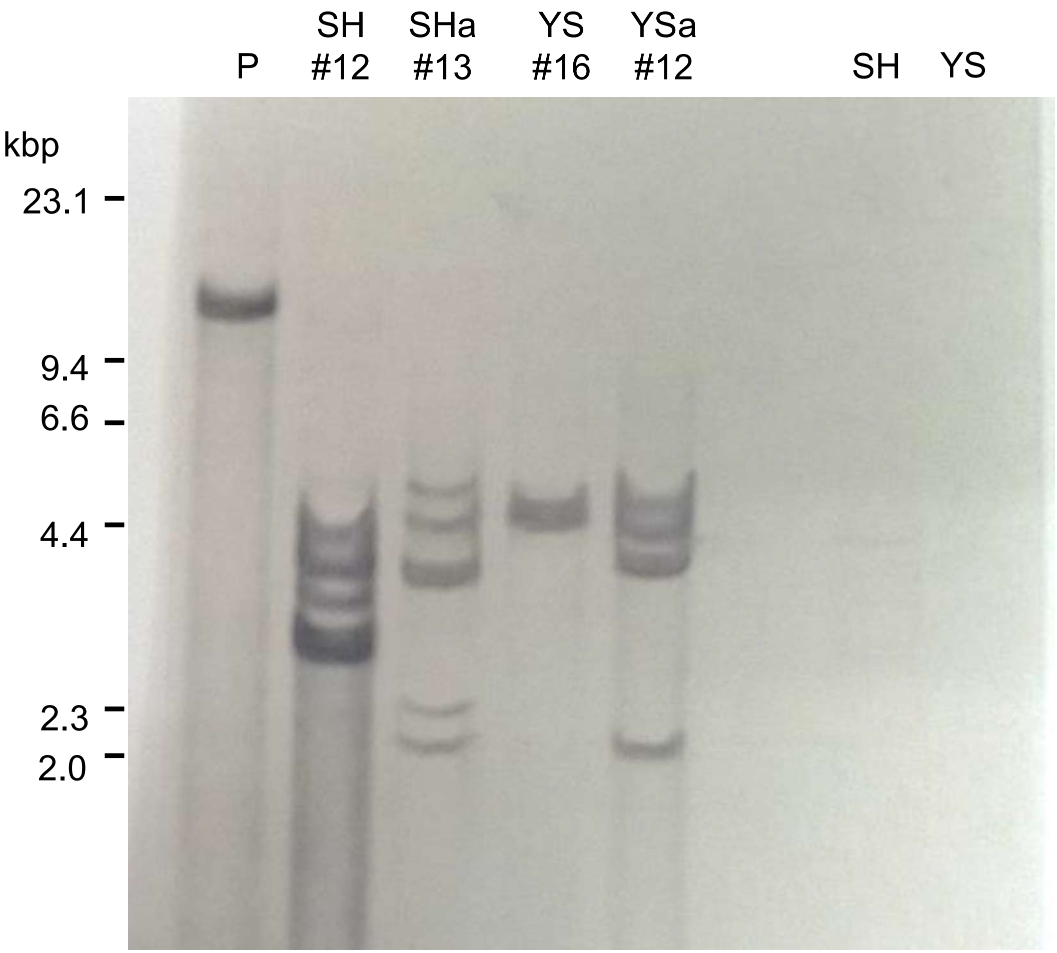

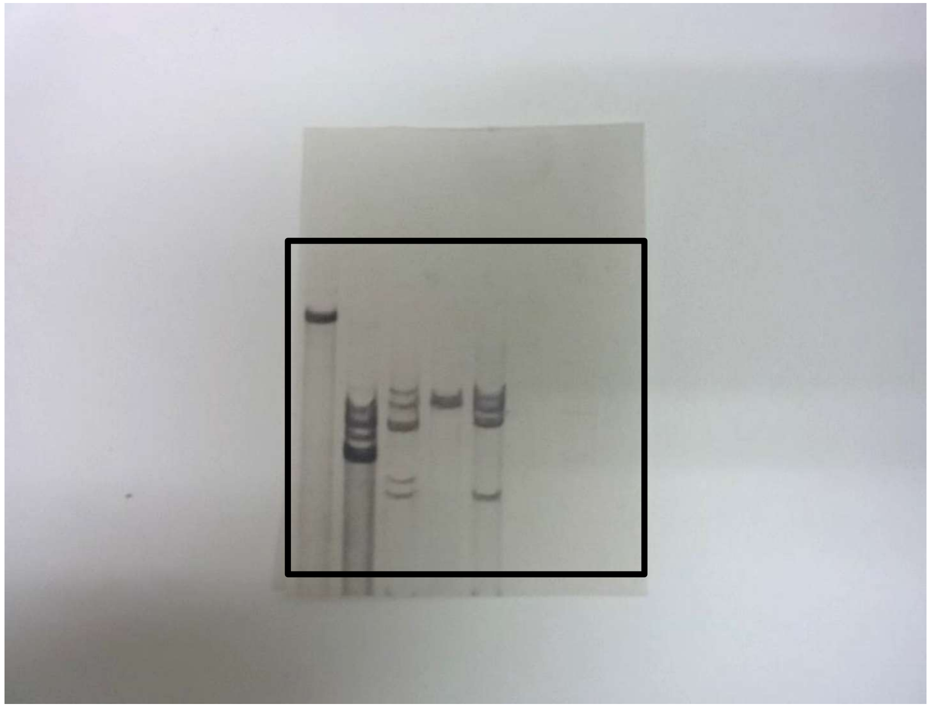

Supplementary Figure S3. Southern blot analysis for detecting transgene integration into the genomes of *CmDMC1*-TALEN chrysanthemum plants.

Genomic DNA was digested with *Xba*I, and hybridized with the *nptII*-specific probe.

Sizes of the molecular markers (λ DNA/*Hin*dIII digest) are indicated on the left.

The section cropped for the figure is indicated as a black rectangle in the original picture (right).

P: plasmid pBIK201DMC-TAL as positive control; SH and YS: DNAs from the non-transgenic control 'Shuho-no-chikara' and 'Yamate-shiro', respectively. SH#12 and SHa#13: DNAs from *CmDMC1*-TALEN lines from 'Shuho-no-chikara'. YS#16 and YSa#12: DNAs from *CmDMC1*-TALEN lines from 'Yamate-shiro', respectively.

```
          DMC1-RNAi F1
Cont.      1 AATCTGTGAAGCTGCTGAGAAGCTAGTGgttagtcctctgctgtttagtacagacttggtttatacgatcttataatgtggcatgtaagtgtttttgctc  100
SH#12      1 ****************************************************************************************************  100
SHa#13     1 ****************************************************************************************************  100
SHb#14     1 ****************************************************************************************************  100
SHc#15     1 ****************************************************************************************************  100
SHc#15     1 ****************************************************************************************************  100
SHd#16     1 ****************************************************************************************************  100
SHe#19     1 ****************************************************************************************************  100
SHf#20     1 ****************************************************************************************************  100
YS#16      1 ****************************************************************************************************  100
YSa#12     1 ****************************************************************************************************  100
YSb#13     1 ****************************************************************************************************  100
YSb#13     1 ****************************************************************************************************  100
YSc#27     1 ****************************************************************************************************  100
YSd#28     1 ****************************************************************************************************  100
YSd#28     1 ****************************************************************************************************  100
YSe#30     1 ****************************************************************************************************  100
YSe#30     1 ****************************************************************************************************  100
YSf#32     1 ****************************************************************************************************  100

Cont.    101 gttactgtttcagAATTATGGTTACATTACTGGAAGCGATGCTCTGCTCAAAgtaagatgctcattgtttaacttctatttgcaaaatgcttagaccgaa  200
SH#12    101 ****************************************************************************************************  200
SHa#13   101 ****************************************************************************************************  200
SHb#14   101 ****************************************************************************************************  200
SHc#15   101 ****************************************************************************************************  200
SHc#15   101 ****************************************************************************************************  200
SHd#16   101 ****************************************************************************************************  200
SHe#19   101 ****************************************************************************************************  200
SHf#20   101 ****************************************************************************************************  200
YS#16    101 ****************************************************************************************************  200
YSa#12   101 ****************************************************************************************************  200
YSb#13   101 ****************************************************************************************************  200
YSb#13   101 ****************************************************************************************************  200
YSc#27   101 ****************************************************************************************************  200
YSd#28   101 ****************************************************************************************************  200
YSd#28   101 ****************************************************************************************************  200
YSe#30   101 ****************************************************************************************************  200
YSe#30   101 ****************************************************************************************************  200
YSf#32   101 ****************************************************************************************************  200

Cont.    201 ttgtgctaacatactaactgaatacctgaatttcacttcttaatgacttgacattctgtcatcctttgttaaacagAGAAAGGCTGTTGTCAAAATCACT  300
SH#12    201 ****************************************************************************************************  300
SHa#13   201 ****************************************************************************************************  300
SHb#14   201 ****************************************************************************************************  300
SHc#15   201 ****************************************************************************************************  300
SHc#15   201 ****************************************************************************************************  300
SHd#16   201 ****************************************************************************************************  300
SHe#19   201 ****************************************************************************************************  300
SHf#20   201 ****************************************************************************************************  300
YS#16    201 ****************************************************************************************************  300
YSa#12   201 ****************************************************************************************************  300
YSb#13   201 ****************************************************************************************************  300
YSb#13   201 ****************************************************************************************************  300
YSc#27   201 ****************************************************************************************************  300
YSd#28   201 ****************************************************************************************************  300
YSd#28   201 ****************************************************************************************************  300
YSe#30   201 ****************************************************************************************************  300
YSe#30   201 ****************************************************************************************************  300
YSf#32   201 ****************************************************************************************************  300

Cont.    301 ACTGGAAGTCAAGCTCTTGATGAGCTTTTGGGAGgtgaaaaacttaacttctgaagttctgatattatctgcattaactaagttgatttgtgcatgacag  400
SH#12    301 ****************************************************************************************************  400
SHa#13   301 ****************************************************************************************************  400
SHb#14   301 ****************************************************************************************************  400
SHc#15   301 ****************************************************************************************************  400
SHc#15   301 ****************************************************************************************************  400
SHd#16   301 ****************************************************************************************************  400
SHe#19   301 ****************************************************************************************************  400
SHf#20   301 ****************************************************************************************************  400
YS#16    301 ****************************************************************************************************  400
YSa#12   301 ****************************************************************************************************  400
YSb#13   301 ****************************************************************************************************  400
YSb#13   301 ****************************************************************************************************  400
YSc#27   301 ****************************************************************************************************  400
YSd#28   301 ****************************************************************************************************  400
YSd#28   301 ****************************************************************************************************  400
YSe#30   301 ****************************************************************************************************  400
YSe#30   301 ****************************************************************************************************  400
YSf#32   301 ****************************************************************************************************  400
```

```
Cont.    401 tataaagttgtagagaattaactaattatcatatatttgtcaagGTGGTATTGAAACTCTGCAAATCACGGAAGCTTTTGGCGAGTTTAGgtgagttcaa  500
SH#12    401 ****************************************************************************************************  500
SHa#13   401 ****************************************************************************************************  500
SHb#14   401 ****************************************************************************************************  500
SHc#15   401 ****************************************************************************************************  500
SHc#15   401 ****************************************************************************************************  500
SHd#16   401 ****************************************************************************************************  500
SHe#19   401 ****************************************************************************************************  500
SHf#20   401 ****************************************************************************************************  500
YS#16    401 ****************************************************************************************************  500
YSa#12   401 ****************************************************************************************************  500
YSb#13   401 ****************************************************************************************************  500
YSb#13   401 ****************************************************************************************************  500
YSc#27   401 ****************************************************************************************************  500
YSd#28   401 ****************************************************************************************************  500
YSd#28   401 ****************************************************************************************************  500
YSe#30   401 ****************************************************************************************************  500
YSe#30   401 ****************************************************************************************************  500
YSf#32   401 ****************************************************************************************************  500

Cont.    501 tcatattttatactggaatctgtatgaatctttatgatctgtgactctgtgtcttactcccataccctgcctaggcgggattgggtctgttgttgttgtt  600
SH#12    501 ****************************************************************************************************  600
SHa#13   501 ****************************************************************************************************  600
SHb#14   501 ****************************************************************************************************  600
SHc#15   501 ****************************************************************************************************  600
SHc#15   501 ****************************************************************************************************  600
SHd#16   501 ****************************************************************************************************  600
SHe#19   501 ****************************************************************************************************  600
SHf#20   501 ****************************************************************************************************  600
YS#16    501 ****************************************************************************************************  600
YSa#12   501 ****************************************************************************************************  600
YSb#13   501 ****************************************************************************************************  600
YSb#13   501 ****************************************************************************************************  600
YSc#27   501 ****************************************************************************************************  600
YSd#28   501 ****************************************************************************************************  600
YSd#28   501 ****************************************************************************************************  600
YSe#30   501 ****************************************************************************************************  600
YSe#30   501 ****************************************************************************************************  600
YSf#32   501 ****************************************************************************************************  600

Cont.    601 gttgttgactctgtgtcttacagcgccccatcaatgtaattgcctaattttcagGTCTGGAAAGACACAGCTGGCTCATACTCTTTGCGTTTCAACACAG  700
SH#12    601 ****************************************************************************************************  700
SHa#13   601 ****************************************************************************************************  700
SHb#14   601 ****************************************************************************************************  700
SHc#15   601 ****************************************************************************************************  700
SHc#15   601 ****************************************************************************************************  700
SHd#16   601 ****************************************************************************************************  700
SHe#19   601 ****************************************************************************************************  700
SHf#20   601 ****************************************************************************************************  700
YS#16    601 ****************************************************************************************************  700
YSa#12   601 ****************************************************************************************************  700
YSb#13   601 ****************************************************************************************************  700
YSb#13   601 ****************************************************************************************************  700
YSc#27   601 ****************************************************************************************************  700
YSd#28   601 ****************************************************************************************************  700
YSd#28   601 ****************************************************************************************************  700
YSe#30   601 ****************************************************************************************************  700
YSe#30   601 ****************************************************************************************************  700
YSf#32   601 ****************************************************************************************************  700

Cont.    701 gtactctcttctatgagacttcttgtggtcacatgagttagtttgactttaatgttccaacttccaattgatgggttatccagattggcatgttgctgct  800
SH#12    701 ****************************************************************************************************  800
SHa#13   701 ****************************************************************************************************  800
SHb#14   701 ****************************************************************************************************  800
SHc#15   701 ****************************************************************************************************  800
SHc#15   701 ****************************************************************************************************  800
SHd#16   701 ****************************************************************************************************  800
SHe#19   701 ****************************************************************************************************  800
SHf#20   701 ****************************************************************************************************  800
YS#16    701 ****************************************************************************************************  800
YSa#12   701 ****************************************************************************************************  800
YSb#13   701 ****************************************************************************************************  800
YSb#13   701 ****************************************************************************************************  800
YSc#27   701 ****************************************************************************************************  800
YSd#28   701 ****************************************************************************************************  800
YSd#28   701 ****************************************************************************************************  800
YSe#30   701 ****************************************************************************************************  800
YSe#30   701 ****************************************************************************************************  800
YSf#32   701 ****************************************************************************************************  800
```

```
Cont.    801 tgtggttttcctttttactatctaatattgttggtttttcttgaagtgctgtcttaaatttcactatacatcacttaacaactttcaaattattgcaag  900
SH#12    801 ****************************************************************************************************  900
SHa#13   801 ****************************************************************************************************  900
SHb#14   801 ****************************************************************************************************  900
SHc#15   801 ****************************************************************************************************  900
SHc#15   801 ****************************************************************************************************  900
SHd#16   801 ****************************************************************************************************  900
SHe#19   801 ****************************************************************************************************  900
SHf#20   801 ****************************************************************************************************  900
YS#16    801 ****************************************************************************************************  900
YSa#12   801 ****************************************************************************************************  900
YSb#13   801 ****************************************************************************************************  900
YSb#13   801 ****************************************************************************************************  900
YSc#27   801 ****************************************************************************************************  900
YSd#28   801 ****************************************************************************************************  900
YSd#28   801 ****************************************************************************************************  900
YSe#30   801 ****************************************************************************************************  900
YSe#30   801 ****************************************************************************************************  900
YSf#32   801 ****************************************************************************************************  900

Cont.    901 ggacactcgctatgatatattgatgttaaccagttatgacttatgatttatgacgtggaactagtatctcttattcatttaatgctgaccattcttctta 1000
SH#12    901 **************************************************************************************************** 1000
SHa#13   901 **************************************************************************************************** 1000
SHb#14   901 **************************************************************************************************** 1000
SHc#15   901 **************************************************************************************************** 1000
SHc#15   901 **************************************************************************************************** 1000
SHd#16   901 **************************************************************************************************** 1000
SHe#19   901 **************************************************************************************************** 1000
SHf#20   901 **************************************************************************************************** 1000
YS#16    901 **************************************************************************************************** 1000
YSa#12   901 **************************************************************************************************** 1000
YSb#13   901 **************************************************************************************************** 1000
YSb#13   901 **************************************************************************************************** 1000
YSc#27   901 **************************************************************************************************** 1000
YSd#28   901 **************************************************************************************************** 1000
YSd#28   901 **************************************************************************************************** 1000
YSe#30   901 **************************************************************************************************** 1000
YSe#30   901 **************************************************************************************************** 1000
YSf#32   901 **************************************************************************************************** 1000

Cont.   1001 atccatgcagCTTCCTACTAGCATGAAAGGTGGGAATGGGAAGGTTGCTTATATTGATACTGTGGGAACTTTCgtatccttgattatagtttgatcacat 1100
SH#12   1001 **************************************************************************************************** 1100
SHa#13  1001 **************************************************************************************************** 1100
SHb#14  1001 **************************************************************************************************** 1100
SHc#15  1001 **************************************************************************************************** 1100
SHc#15  1001 **************************************************************************************************** 1100
SHd#16  1001 **************************************************************************************************** 1100
SHe#19  1001 **************************************************************************************************** 1100
SHf#20  1001 **************************************************************************************************** 1100
YS#16   1001 **************************************************************************************************** 1100
YSa#12  1001 **************************************************************************************************** 1100
YSb#13  1001 **************************************************************************************************** 1100
YSb#13  1001 **************************************************************************************************** 1100
YSc#27  1001 **************************************************************************************************** 1100
YSd#28  1001 **************************************************************************************************** 1100
YSd#28  1001 **************************************************************************************************** 1100
YSe#30  1001 **************************************************************************************************** 1100
YSe#30  1001 **************************************************************************************************** 1100
YSf#32  1001 **************************************************************************************************** 1100

Cont.   1101 ctaattcaatccagttcattttacctctgtgactttcgctttggtgatgacacttgcgaataagaaaaaaataaaatgatagcctttgcgtgtcattcct 1200
SH#12   1101 **************************************************************************************************** 1200
SHa#13  1101 **************************************************************************************************** 1200
SHb#14  1101 **************************************************************************************************** 1200
SHc#15  1101 **************************************************************************************************** 1200
SHc#15  1101 **************************************************************************************************** 1200
SHd#16  1101 **************************************************************************************************** 1200
SHe#19  1101 **************************************************************************************************** 1200
SHf#20  1101 **************************************************************************************************** 1200
YS#16   1101 **************************************************************************************************** 1200
YSa#12  1101 **************************************************************************************************** 1200
YSb#13  1101 **************************************************************************************************** 1200
YSb#13  1101 **************************************************************************************************** 1200
YSc#27  1101 **************************************************************************************************** 1200
YSd#28  1101 **************************************************************************************************** 1200
YSd#28  1101 **************************************************************************************************** 1200
YSe#30  1101 **************************************************************************************************** 1200
YSe#30  1101 **************************************************************************************************** 1200
YSf#32  1101 **************************************************************************************************** 1200
```

```
                                                                                                     DMC1-gF1
Cont.  1201 taatataatgtgcttgcagCGACCTGATCGTATTGTACCCATTGCTGAAAGGTTTGGCATGGATCCTGGAGCTGTACTTGATAATgtaacgatgcttctt 1300
SH#12  1201 **************************************************************************************************** 1300
SHa#13 1201 **************************************************************************************************** 1300
SHb#14 1201 **************************************************************************************************** 1300
SHc#15 1201 **************************************************************************************************** 1300
SHc#15 1201 **************************************************************************************************** 1300
SHd#16 1201 **************************************************************************************************** 1300
SHe#19 1201 **************************************************************************************************** 1300
SHf#20 1201 **************************************************************************************************** 1300
YS#16  1201 **************************************************************************************************** 1300
YSa#12 1201 **************************************************************************************************** 1300
YSb#13 1201 **************************************************************************************************** 1300
YSb#13 1201 **************************************************************************************************** 1300
YSc#27 1201 **************************************************************************************************** 1300
YSd#28 1201 **************************************************************************************************** 1300
YSd#28 1201 **************************************************************************************************** 1300
YSe#30 1201 **************************************************************************************************** 1300
YSe#30 1201 **************************************************************************************************** 1300
YSf#32 1201 **************************************************************************************************** 1300

Cont.  1301 tctcactctatattgctggtttgtatggtaactaattgcttcttctgtcacatattctgaagATTATCTATGCACGTGCTTACACATACGAGCATCAATA 1400
SH#12  1301 **************************************************************************************************** 1400
SHa#13 1301 **************************************************************************************************** 1400
SHb#14 1301 **************************************************************************************************** 1400
SHc#15 1301 **************************************************************************************************** 1400
SHc#15 1301 **************************************************************************************************** 1400
SHd#16 1301 **************************************************************************************************** 1400
SHe#19 1301 **************************************************************************************************** 1400
SHf#20 1301 **************************************************************************************************** 1400
YS#16  1301 **************************************************************************************************** 1400
YSa#12 1301 **************************************************************************************************** 1400
YSb#13 1301 **************************************************************************************************** 1400
YSb#13 1301 **************************************************************************************************** 1400
YSc#27 1301 **************************************************************************************************** 1400
YSd#28 1301 **************************************************************************************************** 1400
YSd#28 1301 **************************************************************************************************** 1400
YSe#30 1301 **************************************************************************************************** 1400
YSe#30 1301 **************************************************************************************************** 1400
YSf#32 1301 **************************************************************************************************** 1400

        TALEN recognition sequence
            5′ upstream (TAL-L)                3′ downstream (TAL-R)
Cont.  1401 CAACCTGCTCCTTGGTTTGGCAGCAAAGATGGCTGAAG-AACCTTTCAGACTACTGgtgggtctttcttgagttaactgttcctaacaagtcaaacctgta 1500
SH#12  1401 ***********************-**************-************************************************************** 1499
SHa#13 1401 **************************************-************************************************************** 1500
SHb#14 1401 ***********************--*************-************************************************************** 1498
SHc#15 1401 ***********************-**************-************************************************************** 1499
SHc#15 1401 **************************************G************************************************************** 1501
SHd#16 1401 **********************-***************-************************************************************** 1499
SHe#19 1401 *************************--***********-************************************************************** 1498
SHf#20 1401 ***********************-**************-************************************************************** 1499
YS#16  1401 **********************-***************-************************************************************** 1499
YSa#12 1401 **************************************-************************************************************** 1500
YSb#13 1401 *************************-************-************************************************************** 1499
YSb#13 1401 *************************--***********-************************************************************** 1498
YSc#27 1401 *********************-****************-************************************************************** 1499
YSd#28 1401 ********************-*****************-************************************************************** 1499
YSd#28 1401 ********************--****************-************************************************************** 1498
YSe#30 1401 *************************-************-************************************************************** 1499
YSe#30 1401 **************************************G************************************************************** 1501
YSf#32 1401 ********************--****************-************************************************************** 1498
                                                              Walker B

                                                                                                    DMC1-gR1
Cont.  1501 cttttttgtatactgattcacactccgaatgtaattatttcccagATTGTTGATTCTGTCATTGCTCTGTTCCGAGTTGACTTCACTGGAAGAGGAGAAC 1600
SH#12  1500 **************************************************************************************************** 1599
SHa#13 1501 **************************************************************************************************** 1600
SHb#14 1499 **************************************************************************************************** 1598
SHc#15 1500 **************************************************************************************************** 1598
SHc#15 1502 **************************************************************************************************** 1601
SHe#19 1499 **************************************************************************************************** 1598
SHf#20 1500 **************************************************************************************************** 1599
YS#16  1500 **************************************************************************************************** 1599
YSa#12 1501 **************************************************************************************************** 1600
YSb#13 1500 **************************************************************************************************** 1599
YSb#13 1499 **************************************************************************************************** 1598
YSc#27 1500 **************************************************************************************************** 1599
YSd#28 1500 **************************************************************************************************** 1599
YSd#28 1499 **************************************************************************************************** 1598
YSe#30 1500 **************************************************************************************************** 1599
YSe#30 1502 **************************************************************************************************** 1601
YSf#32 1499 **************************************************************************************************** 1598
                                                              Walker B
```

```
Cont.   1601 TTGCAGAGCGCCAGgtttatagcttctgcttttgcttttagatcttctttatgcttctatgtgtttgatcataaatgacatcatttgtgcatataatact 1700
SH#12   1600 **************************************************************************************************** 1699
SHa#13  1601 **************************************************************************************************** 1700
SHb#14  1599 **************************************************************************************************** 1698
SHc#15  1600 **************************************************************************************************** 1998
SHc#15  1602 **************************************************************************************************** 1701
SHd#16  1600 **************************************************************************************************** 1699
SHe#19  1599 **************************************************************************************************** 1698
SHf#20  1600 **************************************************************************************************** 1699
YS#16   1600 **************************************************************************************************** 1699
YSa#12  1601 **************************************************************************************************** 1700
YSb#13  1600 **************************************************************************************************** 1699
YSb#13  1599 **************************************************************************************************** 1698
YSc#27  1600 **************************************************************************************************** 1699
YSd#28  1600 **************************************************************************************************** 1699
YSd#28  1599 **************************************************************************************************** 1698
YSe#30  1600 **************************************************************************************************** 1699
YSe#30  1602 **************************************************************************************************** 1701
YSf#32  1599 **************************************************************************************************** 1698

                                                                                            DMC1-RNAi R1
Cont.   1701 tattgcagCAAAAGCTGGCTCAGATGCTCTCCCGATTGACCAAGATTGCTGAAGAATTTAACGTTGCAGTGTATATGACAAA 1782 (leaves + roots)
SH#12   1700 ********************************************************************************** 1781 (13 + 16)
SHa#13  1701 ********************************************************************************** 1782 (15 + 14)
SHb#14  1700 ********************************************************************************** 1780 (14 + 13)
SHc#15  1700 ********************************************************************************** 1781 ( 9 + 10)
SHc#15  1702 ********************************************************************************** 1783 ( 7 +  7)
SHd#16  1699 ********************************************************************************** 1781 (16 + 15)
SHe#19  1700 ********************************************************************************** 1780 (15 + 13)
SHf#20  1700 ********************************************************************************** 1781 (17 + 16)
YS#16   1700 ********************************************************************************** 1781 (15 + 16)
YSa#12  1701 ********************************************************************************** 1782 (16 + 17)
YSb#13  1700 ********************************************************************************** 1781 ( 8 +  9)
YSb#13  1699 ********************************************************************************** 1780 ( 8 +  7)
YSc#27  1700 ********************************************************************************** 1781 (16 + 15)
YSd#28  1700 ********************************************************************************** 1781 ( 9 +  8)
YSd#28  1699 ********************************************************************************** 1780 ( 9 +  8)
YSe#30  1700 ********************************************************************************** 1781 ( 7 +  8)
YSe#30  1702 ********************************************************************************** 1783 ( 9 +  9)
YSf#32  1699 ********************************************************************************** 1781 (17 + 14)
```

Supplementary Figure S4. Mutation patterns in the *CmDMC1a* gene from 14 *CmDMC1*-TALEN lines.
DNA sequences were aligned with the DNASIS software (version 3.7, Hitachi Software Engineering). Total DNA was prepared from leaves and roots from each *CmDMC1*-TALEN line according to Shinoyama et al.[61]. About 1.8-kbp fragments containing the TALEN recognition sequences (TAL-L and TAL-R) were amplified by PCR using a primer pair DMC1-RNAi F1 (5′-aatctgtgaagctgct-3′) and DMC1-RNAi R1 (5′-tttgtcatatacac-3′) and the above-mentioned DNAs as templates. Each amplicon was cloned and sequenced as described in **METHODS**. Sequences of over 13 clones for the *CmDMC1a* locus (more than 7 clones for each mutation pattern) were analyzed for each *CmDMC1*-TALEN line. The lines shown in magenta and orange carried two different mutation patterns in the *CmDMC1a* locus (Table 1 and Supplementary Table S5 online). Numbers in parentheses indicate those of sequenced clone inserts from leaves + roots.

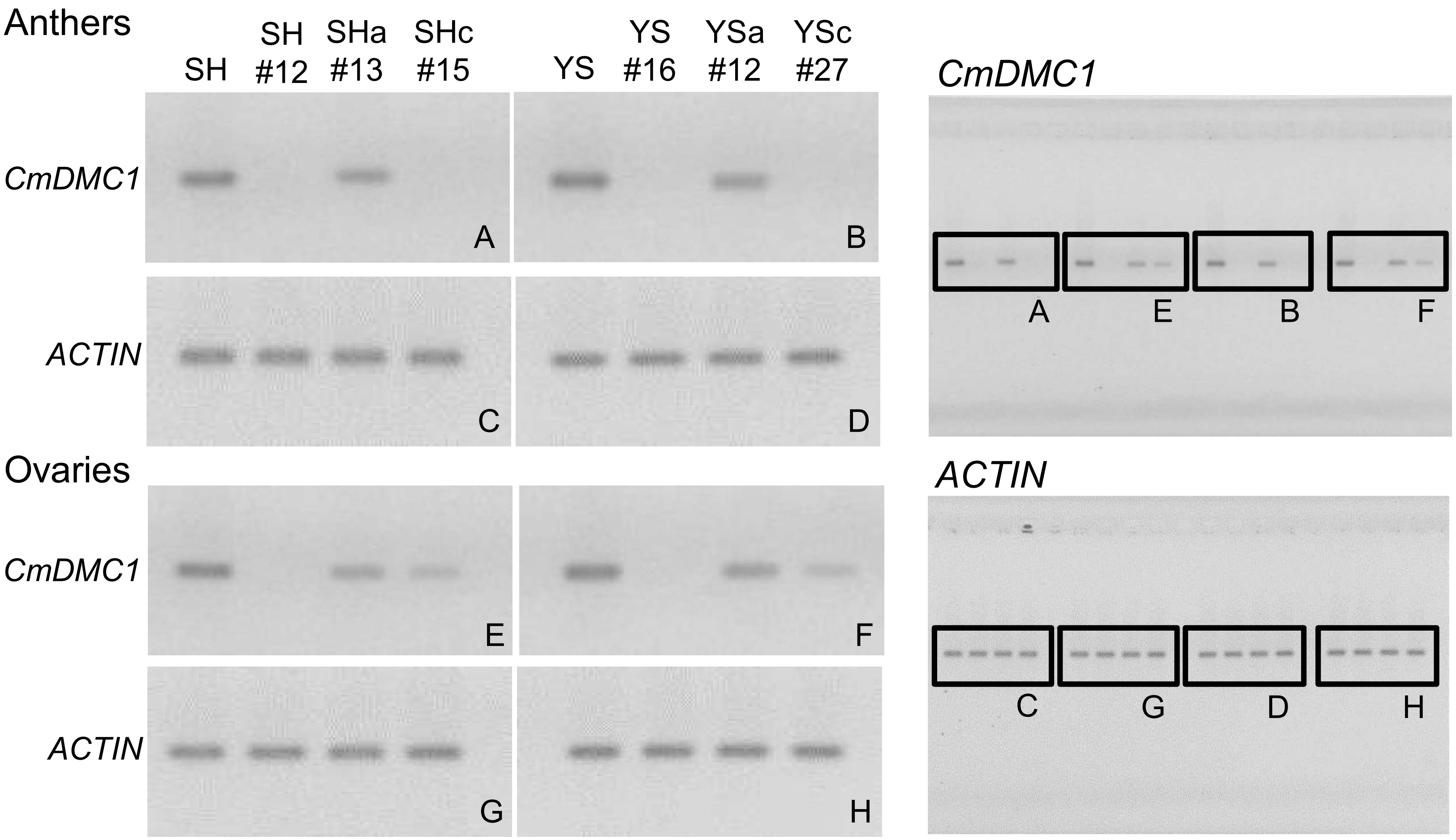

Supplementary Figure S5. Detection of *CmDMC1* transcript in anthers and ovaries of *CmDMC1*-TALEN chrysanthemums by northern blot analysis.

Total RNAs were isolated from anthers (at early meiotic division stage before tetrad formation) and ovaries of *CmDMC1*-TALEN lines and non-transgenic control grown at 20 ˚C. Twenty µg of total RNA was applied to each lane. The RNA blots were probed with a 1,032-bp fragment of *CmDMC1a* gene, and 1,134-bp of the *ACTIN* gene of *Ch. morifolium*.

SH and YS: Non-transgenic controls 'Shuho-no-chikara' and 'Yamate-shiro', respectively. SH#12, SHa#13 and SHc#15 in panels A, C, E and G: *CmDMC1*-TALEN lines from 'Shuho-no-chikara'; YS#16, YSa#12 and YSc#27 in panels B, D, F and H: *CmDMC1*-TALEN lines from 'Yamate-shiro'. Panels A, B, C and D show RNA blots of anthers and panels E, F, G and H show RNA blots of ovaries.

The sections cropped for panels A to H are indicated as black rectangles in the corresponding original pictures on the right. The RNA blots in panels A to H are identical to the respective panels in Fig. 3.

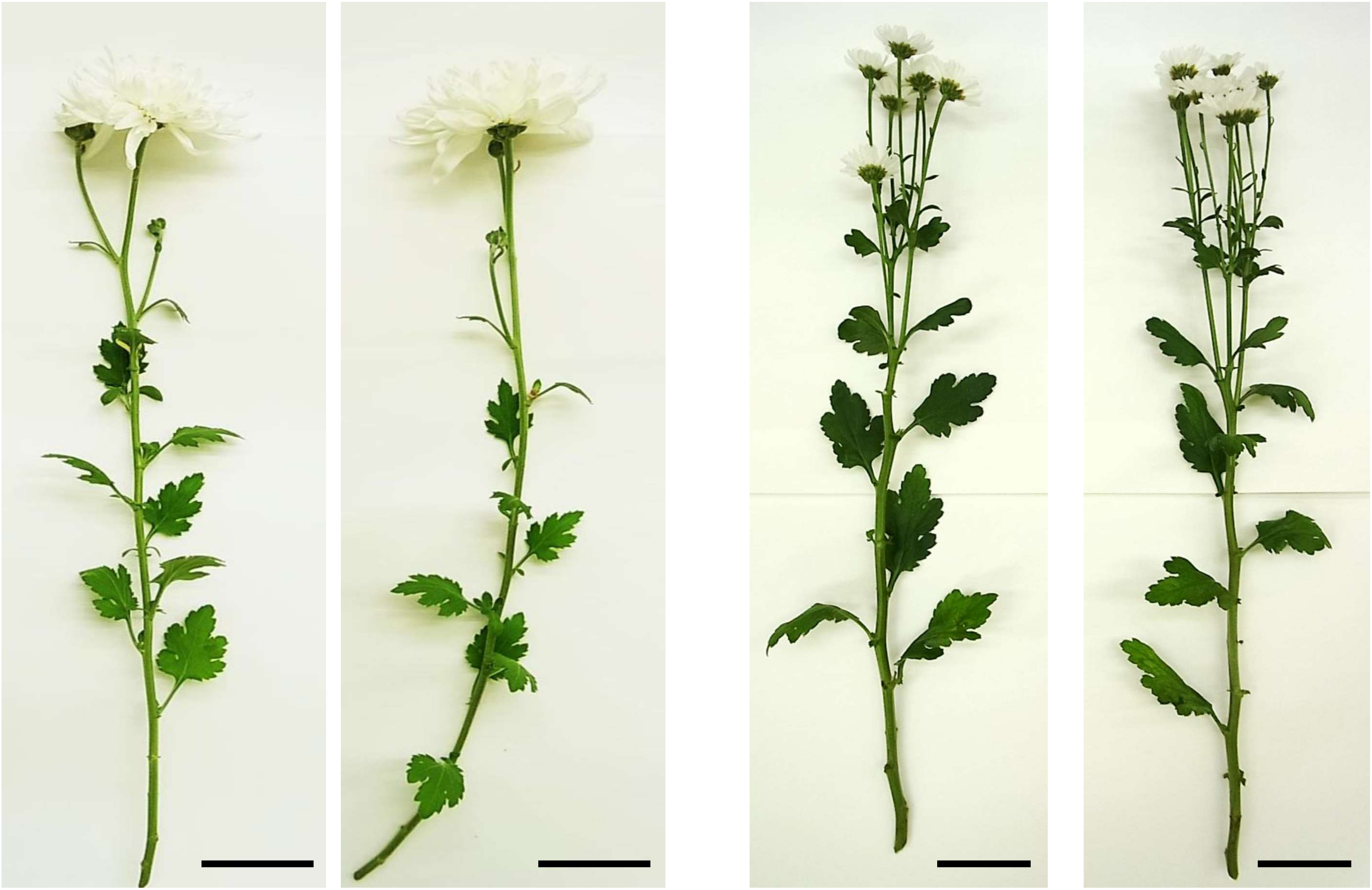

Non-transgenic control 'Shuho-no-chikara' | *CmDMC1*-edited line SH#12 | Non-transgenic control 'Yamate-shiro' | *CmDMC1*-edited line YS#16

Supplementary Figure S6. Morphology of mature plants from *CmDMC1*-edited lines and non-transgenic controls.
These plants were acclimatized and cultivated in a bio-safety containment greenhouse at 20 ˚C under natural daylength. Photographs were taken at Fukui Agricultural Experiment Station by H. Shinoyama.
Scale bars = 5 cm.

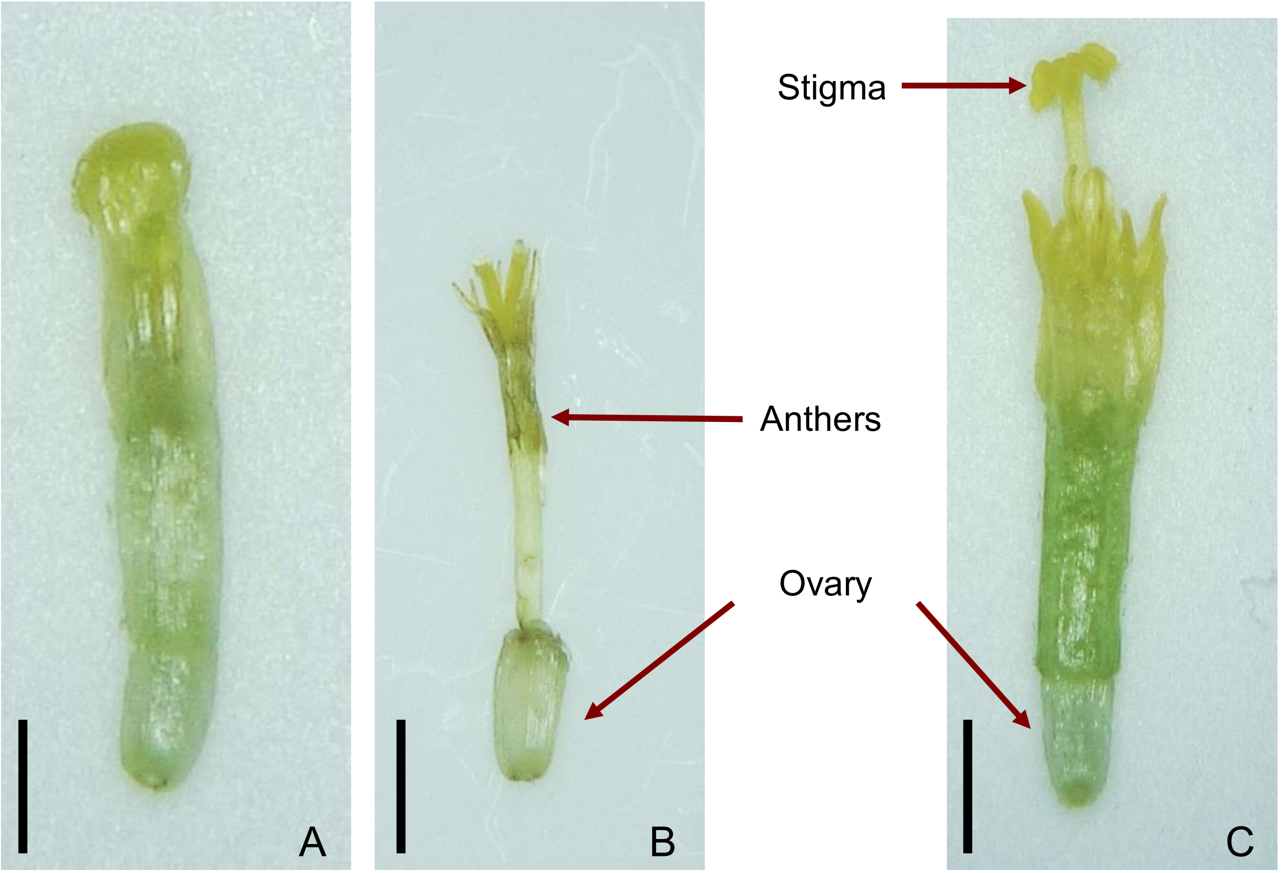

Supplementary Figure S7. Morphology of tubular flowers of chrysanthemum.
(A) A tubular flower 1 day before flowering.
(B) A tubular flower after removal of corolla (five petals were fused together) from the tubular flower (A).
(C) A tubular flower 2–3 days after flowering.
Scale bars = 1 mm.

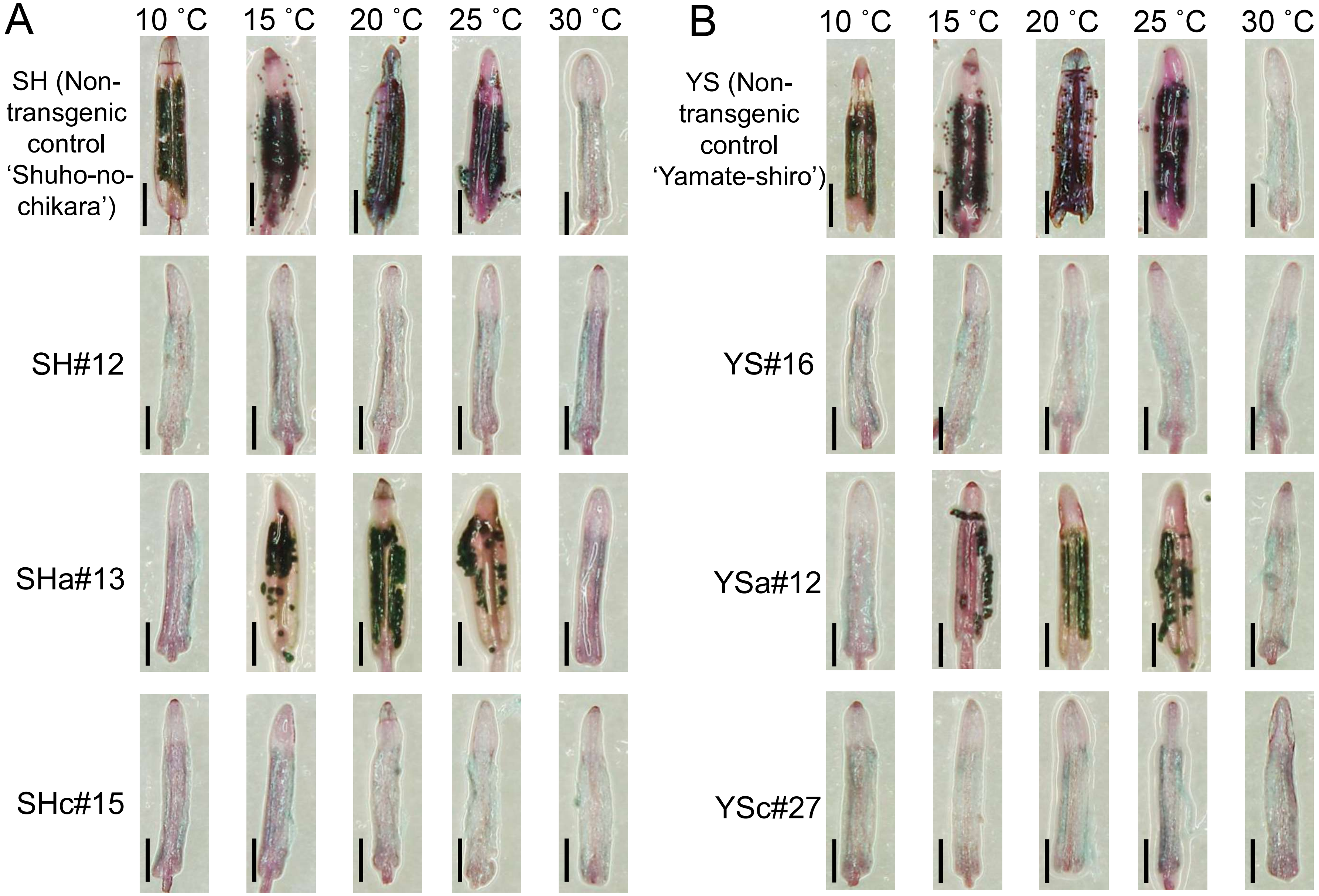

Supplementary Figure S8. Viable stain of pollen grains from *CmDMC1*-TALEN chrysanthemum plants grown under different temperatures. One day before flowering, anthers were stained with Alexander staining solution[22].

A: Non-transgenic control ‘Shuho-no-chikara’ and the *CmDMC1*-TALEN lines, SH#12, SHa#13 and SHc#15.

B: Non-transgenic control ‘Yamate-shiro’ and the *CmDMC1*-TALEN lines, YS#16, YSa#12 and YSc#27.

Scale bars = 0.2 mm.

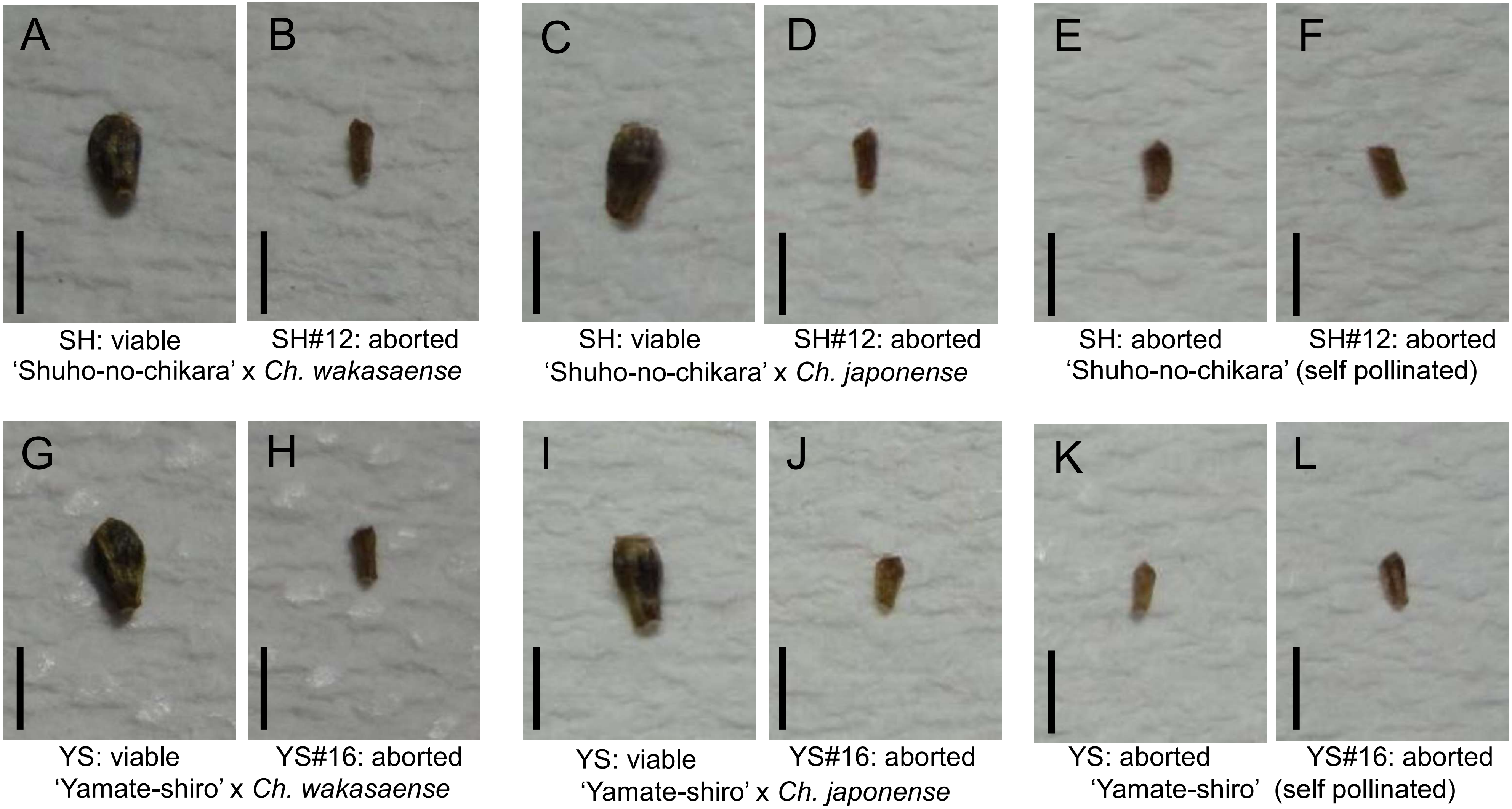

Supplementary Figure S9. Morphology of F_1 seeds from crossing between *CmDMC1*-TALEN lines and their wild relatives.

A, C, G and I: Viable F_1 seeds 2 months after crossing. B, D, H and J: Aborted F_1 seeds 2 months after crossing. E, F, K and L: Aborted seeds 2 months after self pollination. Scale bars = 1 mm.
